# A reproducible extended ex-vivo normothermic machine liver perfusion protocol utilising improved nutrition and targeted vascular flows

**DOI:** 10.1038/s43856-024-00636-2

**Published:** 2024-10-24

**Authors:** George Clarke, Jingwen Mao, Angus Hann, Yiyu Fan, Amita Gupta, Anisa Nutu, Erwin Buckel Schaffner, Kayani Kayani, Nicholas Murphy, Mansoor N. Bangash, Anna L. Casey, Isla Wootton, Alexander J. Lawson, Bobby V. M. Dasari, M. Thamara P. R. Perera, Hynek Mergental, Simon C. Afford

**Affiliations:** 1 Liver Unit, Queen Elizabeth Hospital Birmingham, Birmingham, B15 2TH UK; 2 Birmingham Biomedical Research Centre, National Institute for Health Research (NIHR), University of Birmingham and University Hospitals Birmingham NHS Foundation Trust, Birmingham, B15 2TH UK; 3 Centre for Liver and Gastrointestinal Research, Institute of Immunology and Immunotherapy, University of Birmingham, Birmingham, B15 2TH UK; 4 Ochre-Bio Ltd, Oxford, UK; 5 Queen Elizabeth Hospital Birmingham, Birmingham, B15 2TH UK; 6 Intensive Care Unit, Queen Elizabeth Hospital Birmingham, Birmingham, B15 2TH UK; 7 Birmingham Acute Care Research Group, Institute of Inflammation and Ageing, University of Birmingham, Birmingham, B15 2TH UK; 8 Microbiology Department, Queen Elizabeth Hospital Birmingham, Birmingham, B15 2TH UK; 9 Clinical Biochemistry, Queen Elizabeth Hospital Birmingham, Birmingham, B15 2TH UK

**Keywords:** Physiology, Hepatology

## Abstract

**Background:**

Normothermic machine perfusion of donor livers has become standard practice in the field of transplantation, allowing the assessment of organs and safe extension of preservation times. Alongside its clinical uses, there has been expanding interest in extended normothermic machine perfusion (eNMP) of livers as a potential vehicle for medical research. Reproducible extended normothermic machine perfusion has remained elusive due to its increased complexity and monitoring requirements. We set out to develop a reproducible protocol for the extended normothermic machine perfusion of whole human livers.

**Methods:**

Human livers declined for transplantation were perfused using a blood-based perfusate at 36 °C using the Liver Assist device (XVIVO, Sweden), with continuous veno-venous haemofiltration in-parallel. We developed the protocol in a stepwise fashion.

**Results:**

Perfusion techniques utilised included: targeted physiological vascular flows, phosphate replacement (to prevent hypophosphataemia), N-acetylcysteine (to prevent methaemoglobin accumulation), and the utilisation of sodium lactate as both a nutritional source and real-time measure of hepatocyte function. All five human livers perfused with the developed protocol showed preserved function with a median perfusion time of 168 h (range 120–184 h), with preserved viability throughout.

**Conclusions:**

Livers can be reproducibly perfused in excess of 120 (range 121–184) hours with evidence of preserved hepatocyte and cholangiocyte function.

## Abstract (web summary)

Clarke et al. present a reproducible protocol for the extended normothermic machine perfusion of human livers. Function and are preserved in five human livers perfused between 121–184 h.

## Plain Language Summary

Circulating blood through human livers at normal body temperature allows transplant surgeons to assess the function of the liver and safely extend the time it is out of the body prior to transplantation. Extending this perfusion of livers beyond 24 h has proven difficult. We evaluated improved techniques to circulate blood through the liver. We found the improved techniques could enable a machine to be used to reliably perfuse livers for more than 24 h, whilst preserving the function of the liver. Our improved method included varying the blood flow according to liver size and removing waste products from the circulating blood. Using our method could enable more livers to be used successfully in transplant operations, reducing the waiting times for people requiring liver transplantation and improving their quality of life.

## Introduction

Extended normothermic machine perfusion (eNMP) (defined as perfusion ≥ 24 h) is an important evolution of normothermic machine perfusion (NMP) technology, offering significant potential in both the clinical setting and for the development of research models of liver injury and regeneration. In the United Kingdom, machines used for NMP in the clinical setting (Liver Assist & Organox Metra device) are licensed for 6 h and 24 h, respectively^1–4^. Currently, there is little need for extended perfusions in the transplant setting, however it can be used to facilitate important research. This may include utilisation of the liver as an exploratory vehicle for reconditioning and resuscitative therapies, investigating pharmacokinetics and drug efficacy, immune modulation, as well as metabolic and regenerative pathways.

The extended preservation of livers using NMP (up to 72 h) has been shown using porcine livers by Butler et al. and Vogel et al.^5,6^. However, the Zurich research group were the first to publish on their experiences with eNMP of human livers^7,8^. Using their integrated, automated perfusion protocol they were able to perfuse 6 human livers rejected for transplantation for 7 days (60% of perfusions attempted), with preserved metabolic markers and histology^7^. They achieved this using a custom-made liver perfusion device, with technology allowing the automated correction of glucose, osmolality, and vascular flows. This remains one of the most advanced perfusion set-ups published and provided a significant contribution to the NMP field. Eshmuminov et al. highlighted some of the key problems faced when designing their protocol, as well as some of key considerations to extend machine perfusion, which included the need for haemofiltration of the perfusate, using continuous veno-venous haemofiltration (CVVH)^7^. Furthermore, careful hormonal manipulation is required to achieve physiological perfusate glucose levels, and vasopressor infusion to achieve target vascular flows^7,9,10^.

Following this landmark publication, the Zurich research group successfully transplanted a liver from a 29-year-old donor with a 4 cm segment 1 lesion, on a background of abdominal, multi-resistant, bacterial sepsis, following 68 h of eNMP^11^. The transplanted liver had an uneventful reperfusion, and the patient was alive 12 months later, without any liver or other health sequelae. This work highlighted how liver function can be preserved successfully using machine perfusion, and that extended perfusion time was not detrimental to the allograft. Crucially, despite 68 h of ex-situ preservation, the use of eNMP meant that the allograft did not suffer any significant reperfusion injury following implantation. This was demonstrated by the recipient haemodynamic stability during reperfusion and low peak ALT post-operatively (138 U/L)^11^. Thus, eNMP has great potential to impact clinical practice, allowing the opportunity to rescue, recondition and resuscitate marginal organs, making them feasible for transplantation as they fulfil strict viability criteria, allowing further expansion of the available donor pool.

Lau et al. an Australian research group, are the only other centre that have published their experiences with eNMP^12–14^. They differ from the Zurich group by perfusing at ‘true’ normothermic temperatures (36 °C, compared to the Zurich group who perfuse at 34–36 °C) using split-liver grafts, split during NMP^12,13^. The team successfully perfused a split graft for 13 days, the longest documented, with biomarkers consistent with good function.

In this report, we present our contributions to the eNMP field. We develop a protocol that reliably and reproducibly supports extended liver perfusion for over 120 h, comparing favourably to similar previously published protocols (40–60% success). Utilising this protocol, we are able to resuscitate marginal and discarded organs, achieving extended periods of NMP with evidence of preserved function and histology. This article describes the established protocol and the lessons learned during its development.

## Methods

### Procurement of human discarded cadaveric donor livers & ethical approval

All packed red cells and donor livers were sourced from the National Health Service Blood and Transplant (NHSBT). Packed red cells were used within 35 days of venesection. Discarded donor livers were initially retrieved with the intention of transplantation as per NHSBT guidelines. The organs were then transported to our centre following decline for transplantation by all UK centres. Informed consent for use in research was obtained from donor families and next-of-kin by the specialist nurse in organ donation in accordance with NHSBT guidelines. Study approval was obtained from the London-Surrey Borders National Research Ethics Service (ref. 18/WA/0214) and the NHSBT ethics committee, reference 06/Q702/61. All methods were performed in accordance with the relevant national guidelines and regulations, as well as those from the University of Birmingham. No organs or tissues were procured from prisoners.

### Liver preparation

Preparation of the liver was analogous to clinical transplantation. The liver was bathed in University of Wisconsin solution at 4 °C with crushed ice. The inferior vena cava was dissected and cleaned with both infra-hepatic and supra-hepatic ends left open. The portal vein (PV) was cleaned to its bifurcation and the common hepatic artery (HA) dissected to the gastroduodenal artery. If present, accessory or replaced hepatic arteries were anastomosed to a suitable stump (e.g., gastroduodenal artery stump or splenic artery stump). Following removal of redundant tissue, the liver was weighed.

The coeliac trunk was cannulated using either a straight 25 French cannula (XVIVO, Sweden) or 16 French graduated suction catheter (Arygle^TM^, Cardinal Health, United Kingdom) and secured with ligatures. When using a 25 French arterial cannula, it is necessary to anastomose an iliac artery interposition graft to the aortic patch, to allow the graft to be cannulated. The portal vein was cannulated using a curved 25 French cannula (XVIVO, Sweden) and secured with ligatures. The cystic duct was ligated, and common hepatic duct cannulated with a 12 French T-tube. The defect in the gallbladder body was closed using either heavy (0/0) ligatures or a continuous 3/0 prolene suture (Ethicon, Johnson & Johnson). Prior to commencing perfusion, the livers were flushed with 2 litres of 5% dextrose solution at room temperature. The cannulae primed with the perfusion fluid and grafts were then placed into the machine reservoir and connected to the perfusion circuit.

### Perfusion circuit set-up

#### Normothermic machine perfusion device

eNMP was conducted using the Liver Assist device and single-use custom consumable set (XVIVO, Sweden). Two centrifugal pumps propelled dual oxygenated perfusate, providing pulsatile (60 bpm) and continuous flow to the hepatic artery and portal vein respectively. Euroset paediatric, 0-polymethylpentene, microporous, hollow fiber membrane oxygenators (validated for 14 days) provided oxygenation and perfusion pressures were limited to 30–110 mmHg in the arterial circuit and 5–13 mmHg in the portal venous circuit. This device is commercially available and used clinically for both normothermic and hypothermic machine perfusion. It is an open effluent system and has the advantage of allowing targeted vascular flows, temperature control, and permits the incorporation of supplementary devices (i.e. continuous veno-venous haemofiltration).

### Perfusate constitution

A blood based perfusate was prepared using 5 units of research-grade O negative packed red cells, 100 ml of 20% human albumin solution (CSL Behring, USA), and 1000 ml of 5% human albumin solution (CSL Behring, USA). Anticoagulation was achieved by initial bolus of 10,000 units heparin (Wockhardt, UK), followed by a continuous infusion of 833 units/hour. Piperacillin with tazobactam (Fresenius, Germany) was administered for antibiotic prophylaxis with 2.25 g given as a bolus during liver connection followed by 2.25 g/24 hr continuous infusion. To support cholangiocyte and hepatocyte function respectively, sodium taurocholic acid (VWR Chemicals, USA) was infused at 140 mg/hr, and a 500 mg methylprednisolone (Pfizer, UK) bolus was administered during liver connection, followed by a 21 mg/hour infusion. Further details can be found in Supplementary Table and Supplementary Method.

### Perfusion parameters

#### Initiation of perfusion

Temperature was initially set to 25 °C and increased incrementally to 37 °C within 30 minutes of starting NMP. Vascular pressures were initially set to 60 mmHg and 8 mmHg in the HA and PV circuit respectively and subsequently titrated incrementally to achieve target flow rates (hepatic artery pressure, 50–100 mmHg; portal vein pressure, 5–10 mmHg). The fraction of inspired oxygen (FiO_2_) was set at 0.4 with 2 L/min of flow.

#### Oxygen delivery

Oxygen and air were mixed using a Sechrist air/oxygen blender (S3500CP-G, Inspiration Healthcare Ltd., Leicester, UK). Hepatic artery FiO_2_ was manually titrated to achieve a pO_2_ value between 12 and 20 kPa. Sweep rate was manually titrated to increase or decrease pCO_2_ and correct pH (target 7.35–7.45).

#### Vascular Flows

In each perfusion, we set out to replicate normal liver physiology aiming for 25–30 ml/100 g/min in the arterial system, and 75–80 ml/100 g/min in the venous system^15^. Our combined total flow target was 100–110 ml/100 g/min. Through manipulation of vascular pressures and titration of vasoconstrictors (norepinephrine and vasopressin) and vasodilators (epoprostenol) we were able to achieve these targets.

#### Methaemoglobin, Carboxyhaemoglobin, and Oxyhaemoglobin

The concentration of methaemoglobin (metHb), carboxyhaemoglobin (COHb), and oxyhaemoglobin (O_2_Hb) were monitored using hourly blood gas analysis. Our targeted minimum oxyhaemoglobin levels were 80%, with falls below this value leading to partial replacement of perfusion fluids (Supplementary Method). N-acetylcysteine (NAC) was included in the perfusion protocol (200 mg bolus following liver establishment, with continuous 200 mg/hour infusion) to prevent the accumulation of methaemoglobin during eNMP.

### Liver nutrition

The perfusate glucose levels were maintained between 4 and 8 mmol/L. This was achieved using a variable rate insulin infusion (1 IU/ml) and 50% dextrose solution (0.5 g/ml). Amino acids (B. Braun, Germany) were continuously infused into the circuit (10 ml/hr), with added vitamins (Cernevit & phytomenadione). Fifty percent lactate solution (VWR Chemicals, USA) was employed as a primary energy substrate for the liver to utilise, titrating lactate infusion to achieve perfusate lactate concentrations of 2–2.5 mmol/L.

### Assessment of liver physiology and function

Perfusate samples from the HA oxygenator, PV oxygenator, post-hepatic pre-dialysis venous port, bile, and dialysis waste fluid were assessed immediately using a Cobas b 221 point of care system (Roche Diagnostics, USA) blood gas analyser.

#### Lactate challenge

Boluses of exogenous lactate (50% sodium lactate) were added to the perfusate every 24 h aiming for circulating lactate concentrations of 10–15 mmol/L (volume administered varied between organs). During this period, both lactate infusion and CVVH was paused.

Persistently elevated lactate concentrations following ‘lactate challenge’ (defined as ≥ hours) were treated with an infusion of 0.1 IU/hour of glucagon, and subsequent lactate challenges was performed in the presence of a concurrent glucagon infusion (0.1 IU/hour). Time taken to clear lactate below 2.5 mmol/L was recorded, allowing a quantitative measurement of hepatocyte function and assessment of variation in hepatocyte function.

### Continuous Veno-Venous Haemofiltration Device (CVVH)

**Fig. 1:**
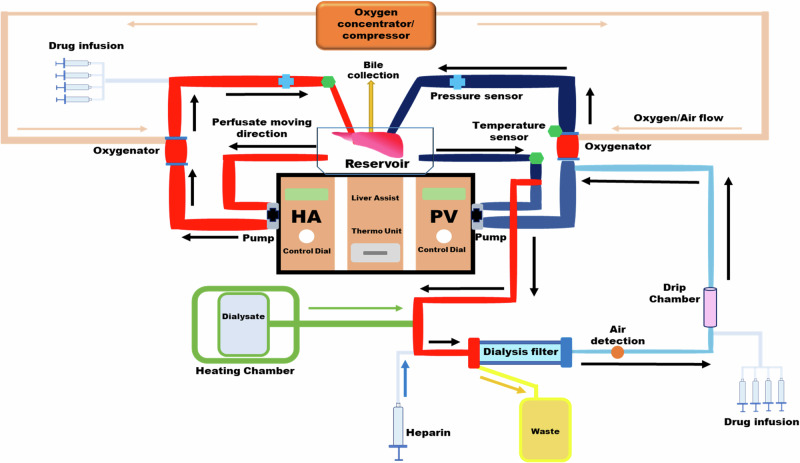
Schematic of the final liver assist set-up. The Liver Assist perfusion device consists of two separate circuits, with a centrifugal pump for both portal vein (PV) and hepatic artery (HA) circuits. The hepatic veins drain into a reservoir (where the liver sits) which is then drains back into the HA and PV circuits. Our continuous veno-venous haemofiltration (CVVH) unit was added in parallel to the portal vein circuit, with afferent blood taken off prior to the PV oxygenator and efferent blood entering back into the PV oxygenator. Heparin was infused into the CVVH circuit. Drugs were infused at two separate points in the circuit: the HA circuit and post-CVVH PV circuit. Figure created in BioRender.

Continuous veno-venous haemofiltration (CVVH) was performed using the Aquarius System (Nikkiso Medical, Europe), a set of single-use adult lines and the HF19 Aquamax filter (Nikkiso Medical, Europe). The set was primed using 5% Human Albumin Solution and attached to the portal circuit of the NMP consumable set. The afferent line was attached to the post-hepatic pre-oxygenator port and the efferent line attached to the portal circuit oxygenator (Fig. 1). CVVH settings are described in detail in Supplementary Method.

Accusol 35 containing 4 mmol/L potassium dialysate solution was used with a physiological, crystalloid, bicarbonate-based buffer (Nikkiso Medical, Europe) (Supplementary Method). Waste fluid was drained into single-use waste bags (Nikkiso Medical, Europe). The single-use adult line set and HF19 Aquamax filter were changed every 72 h as per manufacturer instructions.

### Sample and data collection protocol

Pressure, resistance, and vascular flows were recorded every 60 min. Perfusate samples from the HA oxygenator, PV oxygenator, and post-hepatic pre-dialysis port were taken for blood gas and clinical biochemistry analysis prior to connection of the liver to the perfusion circuit, and every 60 min after initiation of eNMP. Samples for clinical biochemistry were pipetted into a 1.5 ml Eppendorf, whereupon they were centrifuged at 8459 rcf (relative centrifugal force) for 5 min. Supernatant formed was pipetted out and transferred into labelled cryovials which were snap frozen in liquid nitrogen and stored at −80 °C for later analysis. Experiments were not performed under aseptic conditions, and as such, data on micro-organisms grown have not been included.

Bile production was recorded and collected every 3−6 h once perfusion had commenced. Bile was centrifuged at 8459 rcf for 5 min. Bile was then stored in cryovials and snap frozen in liquid nitrogen and stored at −80 °C.

Sixteen-gauge Menghini biopsies were taken pre-perfusion and at set intervals during perfusion. These biopsies were taken from both right and left lobe and snap frozen in liquid nitrogen and stored at −80°C.

Following completion and cessation of perfusion, additional biopsies were taken including peripheral wedge biopsies, cubed core tissue biopsies, hilar hepatic artery biopsies, hilar portal vein biopsies, and hilar bile duct biopsies. These were formalin fixated for paraffin embedding. Cubed core tissue from both left and right lobe were snap frozen in liquid nitrogen and stored at −80 °C.

### Structural and functional histological assessment

Tissue biopsies were formalin fixed and embedded in paraffin blocks for histological evaluation. Slide mounted sections were obtained and prepared for Haematoxylin & Eosin (H&E) and Periodic-acid Schiff (PAS) staining. Quantitative assessment of macrovesicular steatosis and polysaccharides were then performed.

### Statistics and reproducibility

Statistical analysis was performed using GraphPad Prism 9 (GraphPad Software, USA). Continuous variables were compared at each grouped timepoint using an unpaired t-test or a Mann-Whitney U Test as appropriate. Results were considered significant if *p* < 0.05. Due to the logistic and financial demands of the project, we set out a target for five human livers perfused in excess of 120 h using a shared perfusion protocol with evidence of good function throughout.

### Reporting summary

Further information on research design is available in the Nature Portfolio Reporting Summary linked to this article.

## Results

### Donor Demographics and graft details

A total of nine livers, 3 of which were DCD, were included in this study (details in Table 1). The median donor age was 60 (range 42–77) years and the body mass index 30.2 (range 21.46–36.8) kg/m^2^. The median cold ischaemia time (CIT) was 638 (range 540–867) minutes. Of the DCD livers, the mean warm ischaemia time (WIT) was 22 (range 12–38) minutes. Five livers came from male donors (56%). The median pre-perfusion liver weight was 1.86 (range 1.02–3.57) kg. Details of the donor demographics and reasons for discard are provided in Table 1. Livers A-D represent livers perfused during protocol developments, while livers 1–5 were perfused according to the final established protocol.

**Table 1:** Donor demographics for all livers involved in the project (A–D & 1–5)

Perfusion Number	A	B	C	D	1	2	3	4	5
Donor age (years)	50–55	55–59	75–79	60–65	70–75	40–45	70–75	60–65	50–55
Donor Gender	Female	Male	Female	Male	Female	Male	Male	Female	Male
BMI (kg/m^2^)	32.79	28.68	21.46	30.96	36.79	23.77	28.7	31.22	30.15
Cause of death	ICH	HBI	ICH	ICH	ICH	ICH	ICH	CVA	CVA
Donor Type	DBD	DBD	DCD	DBD	DBD	DCD	DBD	DBD	DCD
Donor WIT (mins)	-	-	12	-	-	34	-	-	15
CIT (mins)	638	659	540	680	867	626	622	750	586
Liver weight (g)	1732	1936	1245	1900	1024	2515	1864	1688	3568
Steatosis assessment	Mild/mod	Mild/mod	Nil	Moderate	Moderate	Nil	Mild	Moderate	Severe
Donor Risk Index	1.567	1.505	3.228	1.931	2.260	2.168	2.144	2.447	2.262
Reason for discard	Size/CIT	Biopsy findings	Surgical findings	Steatosis	Congenital defect	WIT	PMH	Steatosis/injury	Steatosis
Total NMP time (hours)	102	100	87	90	121	156	184	168	168

*ICH* Intracranial haemorrhage, *HBI* Hypoxic Brain Injury, *CVA* Cerebral Vascular Accident, *PMH* Past Medical History.

### Perfusion outcomes

**Fig. 2:**
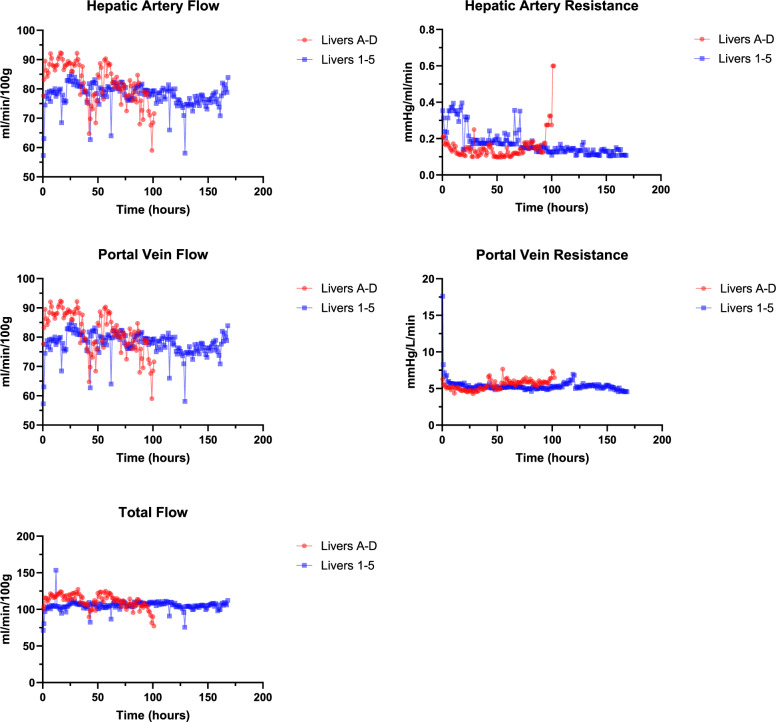
Figure showing median vascular flow and resistance in both artery and vein in the two cohorts: Livers A–D (*n* = 4) displayed in red, and Livers 1–5 (*n* = 5) displayed in blue. The source data for Fig. 2 can be found in Supplementary Data document.

All livers cleared lactate (defined as lactate <2.5 mmol/L) following establishment of eNMP, and achieved target vascular flows, with homogenous perfusion, and evidence of good bile production. The median time to clear lactate was 3 h (range 2–8). Eight out of nine (89%) livers achieved viability as defined by the Birmingham viability criteria^1,16,17^. The median total perfusion time for Livers 1ICH = Intracranial haemorrhage, HBI = Hypoxic Brain Injury, CVA = Cerebral Vascular Accident, PMH = Past Medical History 5 was 168 h (range 121–184) with cessation oonce the targeted perfusion time (initially 120 h, later extended to 168 h) was reached. All livers demonstrated viability at time of perfusion cessation, defined as demonstrating good bile production, a perfusate lactate < 10 mmol/L and achieving target perfusate flows (Fig. 2).

### Methaemoglobin & N-Acetylcysteine

**Fig. 3:**
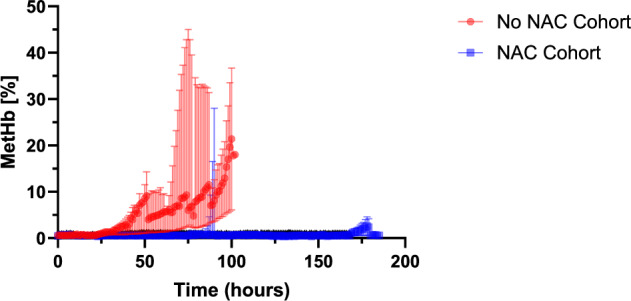
Figure showing median methaemoglobin (%) and range in no NAC cohort (Livers A-C, red, *n* = 3) and NAC cohort (Livers D & 1-5, blue, *n* = 6). The source data for Fig. 3 can be found in Supplementary Data document.

Livers A-C developed methaemoglobinaemia ( > 2%), with the median time to develop being 34 h (range 31–70). The highest methaemoglobin concentration recorded was 43%. In comparison, the NAC group had a median peak MetHb accumulation of 1.3%. In the first 12 h there was no significant difference between the NAC cohort and the control cohort (*p* = 0.909), however there was a significant difference between both groups over 24 h, 48 h, 72 h, and 84 h (*p* = 0.00015, *p* < 0.00001, *p* < 0.00001, *p* < 0.00001, respectively, Fig. 3)^18^.

### Assessment of liver function and viability

#### Lactate challenge

**Fig. 4:**
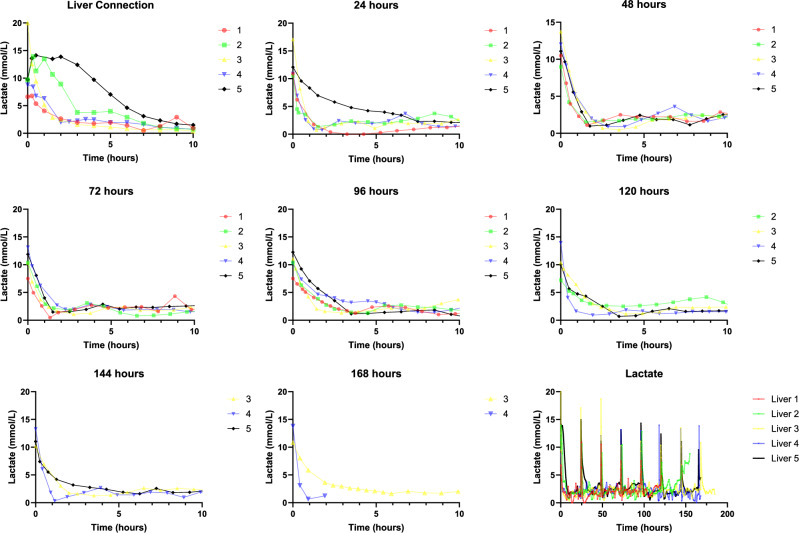
Graphs showing lactate challenges performed at 24 h intervals in Livers 1-5. The source data for Fig. 4 can be found in Supplementary Data document.

Lactate challenges were completed in Livers 1-5 every 24 h (Fig. 4). The median time to clear lactate <2.5 mmol/L following liver connection was 3 h^2–8^, with livers 2 and 5 taking 7 and 8 h, respectively. This is notable, as both Liver 2 and 5 were DCD grafts. Following this, median time to clear exogenous lactate was 1.25 (0.92–7.75), 1.75 (1.1–2.25), 2.75 (1.92–4.33), 2.59 (0.83–11.75), 2.33 (0.83–4.25), and 2.25 (0.58–3.91) hours at 24, 48, 72, 96, 120, 144, and 169 h, respectively.

The rate of lactate clearance each hour was taken as a measure of lactate clearing capacity of each liver. The median rate of lactate clearance following connection is 1.73 (1.47–5.08) mmol/hour. Following this, lactate clearance was 7.98 (1.63–16.03), 5.78 (4.98–7.63), 6.35 (4.97–6.66), 3.51 (2.46–4.77), 3.46 (0.44–14.81), 5.03 (2.03–13.70), and 12.40 (2.13–22.66) mmol/hour at 24, 48, 72, 96, 120, 144, and 168 h, respectively.

### Perfusate stability

**Fig. 5:**
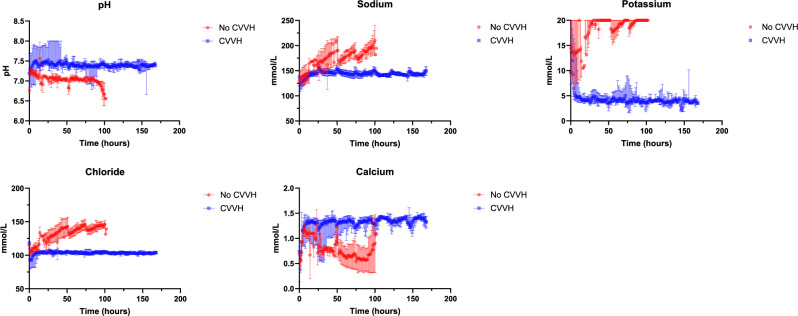
Figure showing median and range of electrolytes in Livers A & B (no CVVH cohort, red, *n* = 2) and Livers C-D & Livers 1-5 (CVVH cohort, blue, *n* = 5). The source data for Fig. 5 can be found in Supplementary Data document.

Two livers were perfused without CVVH in-circuit (Livers A and B), with seven livers perfused with CVVH in-circuit (Livers C, D, and 1-5). CVVH was added as a parallel circuit, with post-hepatic blood diverted into the CVVH circuit before being returned to the portal vein oxygenator (Fig. 1). Addition of CVVH enabled correction of electrolytes (Na^+^, K^+^, Cl^−^, Ca^2+^), maintaining these in their respective physiological ranges. Prior to the addition of CVVH to the circuit, over time the perfusate would develop hypernatremia, hyperkalaemia, hypocalcaemia, and acidosis (Fig. 5, *p* < 0.00001).

#### Phosphate replacement

We measured the perfusate phosphate concentrations in Livers D, 1-5. The dialysate replacement fluid does not include phosphate and as such there is a slow removal from the circuit. The median starting phosphate concentration in the perfusate at Time 0 (before liver connection) is 1.2 mmol/L, which rises rapidly following liver connection to 5.3 mmol/L (at hour one). The median time taken for the phosphate concentration to fall below 1 mmol/L in the circuit was 12 h, in the cohort without phosphate replacement. Within 96 h, the median phosphate concentration drops below measurable levels (<0.2 mmol/L).

Liver 4 and Liver 5 received phosphate replacement at 0.5 mmol/hr and 1.0 mmol/hr, respectively. Time for phosphate concentrations to drop below 1.0 mmol/L was 24 h and 84 h in Liver 4 and 5, respectively. Final phosphate concentrations at hour 168 were 0.86 mmol/L and 1.24 mmol/L at 168 h, respectively.

### Liver injury and integrity assessment

We measured biochemical markers of hepatocyte and cholangiocyte cellular injury to assess the severity of the ischaemia reperfusion injury and the quality of perfusion achieved (Table 2**)**. We observed that the livers perfused with our final eNMP protocol had reduced evidence of hepatocyte injury with lower peak ALT and AST values (*p*=0.0065 and p=0.0000016, respectively), occurring at a median time of 30 h. Markers of cholangiocyte injury showed mixed results with GGT levels showing significant reduction in Livers 1-5, however there was no significant difference between the two cohorts in ALP.

**Table 2:** Table displaying the peak value for each biochemistry test in Livers A-D & Livers 1-5

	A	B	C	D	A-D Median	1	2	3	4	5	1-5 Median	*p*-value
**Peak ALT U/L (hr)**	7915 (66)	9671 (28)	9014 (87)	5335 (90)	8465 (77)	2532 (5)	3731 (30)	3519 (42)	1644 (48)	11577 (1)	3519 (30)	***0.0065***
**Peak AST U/L (h)**	15365 (66)	53418 (28)	18338 (78)	7455 (30)	16852 (48)	4154 (11)	5464 (30)	8235 (30)	4526 (30)	29223 (18)	5464 (30)	***0.0000016***
**Peak ALP U/L (h)**	-	-	4658 (87)	392 (90)	-	211 (120)	300 (156)	632 (72)	152 (48)	1111 (120)	300 (120)	*0.255*
**Peak GGT U/L (h)**	-	714 (28)	568 (36)	430 (90)	568 (36)	196 (120)	188 (23)	479 (72)	118 (18)	704 (90)	196 (72)	***0.0018***
**Peak CRP mg/L (h)**	240 (42)	200 (24)	214 (36)	276 (90)	227 (39)	155 (120)	348 (132)	228 (180)	59 (24)	198 (168)	198 (132)	***0.0067***
**Direct bilirubin (mmol/L)**	165 (42)	-	130 (54)	217 (90)	165 (54)	95 (120)	429 (154)	120 (184)	13 (144)	234 (120)	120 (154)	*0.2128*
**Total bilirubin (mmol/L)**	270 (42)	-	239 (36)	266 (90)	(266 (42)	127 (120)	591 (154)	148 (184)	21 (150)	341 (132)	148 (150)	*0.5384*

The time this occurred for each liver is displayed in hours within the brackets. The source data for Table 2 can be found in Supplementary Data document. Significant *p*-values are noted in bold.

### Cholangiocyte function

Median cumulative bile production was 1996 (723–2537) ml, with Liver 2 producing the most bile, 2537 ml over 154 h. If divided by total perfusion time, this equated to a median bile production of 11.2 (4.3–16.5) ml/hr. If divided further to equate for liver weight, the median bile production per hour per 100 g of liver tissue was 0.7 (0.1–1.0) ml/hr/100 g. When differentiated by donation type, the median cumulative bile production for DBDs tended to be higher than that produced by DCDs, 1996 (1189–2039) ml versus 1630 (723–2537) ml, respectively (*p* = 0.28).

**Fig. 6:**
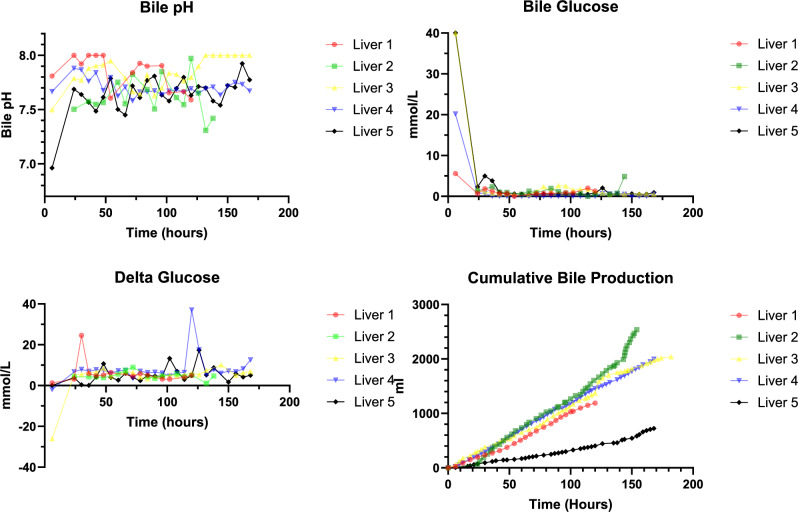
Figure displaying bile pH, bile glucose, delta glucose (perfusate glucose – bile glucose), and cumulative bile production during Livers 1-5. Each Liver is represented by a different colour (Liver 1, red; Liver 2, green; Liver 3, yellow; Liver 4, blue; Liver 5, black). The source data for Fig. 6 can be found in Supplementary Data document.

The bile produced maintained its alkalosis throughout the perfusion time, with hypoglycaemia consistent with preserved cholangiocyte integrity (Fig. 6).

### Histological assessment of sequential biopsies sampled during NMLP

**Fig. 7:**
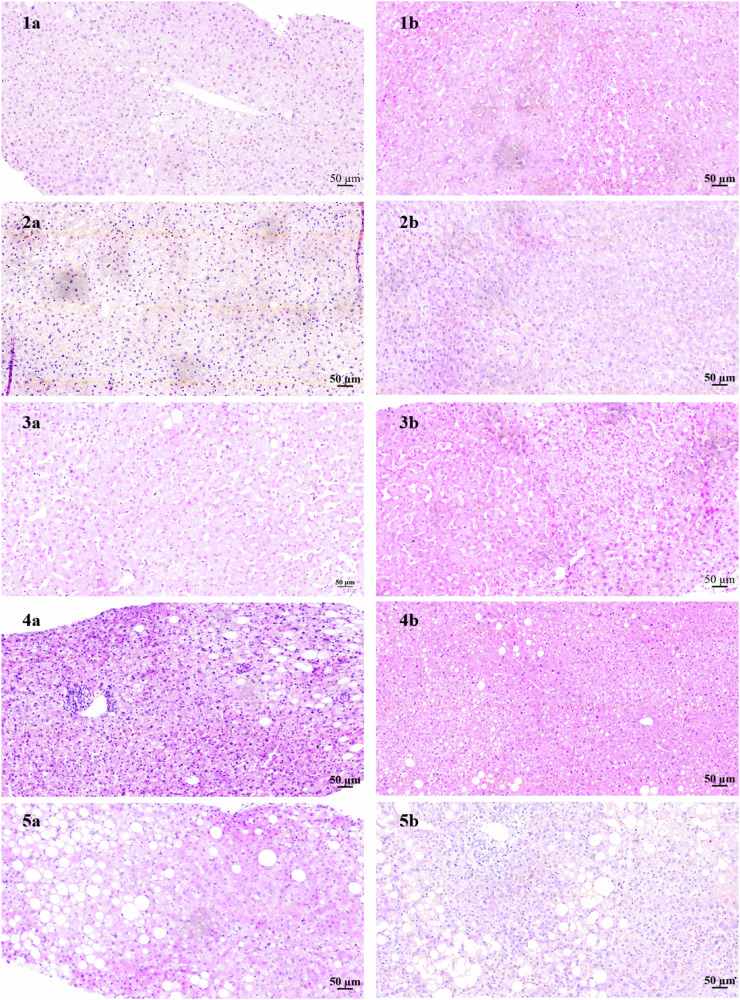
H&E Staining for Livers 1-5. From top to bottom, figures show H&E staining for Livers 1-5 before perfusion (**a**) on the left side, and at the end of perfusion (**b**) on the right side. A 50 *μ*m scale bar is included in the bottom right of each figure.

Menghini biopsies taken at time intervals throughout the course of each perfusion showed features ranging from normal through to mild cellular pathology, including mild macrovesicular steatosis (Fig. 7). There was no association between histological appearance of the parenchyma and individual liver functional performance. At commencement of perfusion all cellular and architectural features were generally normal, occasionally showing positive but negligible cellular features conventionally associated with ischaemia reperfusion injury (IRI), consequence of organ retrieval. The most notable change was the slow progressive appearance of ballooned hepatocytes emanating from zone 3. However even in the most marked cases, this was not associated with poor functional outputs including lactate clearance or bile production. Periodic Acid Schiff staining was used to assess hepatocyte glycogen sequestration which had a somewhat patchy appearance ranging from weak to strongly positive throughout the perfusion, but with no correlative changes. Intrahepatic bile ducts and vessels (sinusoids, portal and central collecting veins) were well preserved throughout with good morphological appearance. A modest mixed mononuclear cell infiltrate remained present but with no change throughout.

## Discussion

In this study, we were able to achieve successful perfusion up to and in excess of five days in all livers utilised, using marginal livers rejected for transplantation by all UK centres. This study to our knowledge represents the first to demonstrate successful extended normothermic machine perfusion whilst retaining graft functional integrity and viability in all livers perfused.

The physiological requirement of a human liver is approximately 100 ml/100 g/min of total blood flow, with 75−80% via the portal vein and the remainder through the hepatic artery. During our perfusions, we were able to achieve our target physiological flows in all livers, without significant difficulty. Titration of vascular pressures and vasodilators enabled this. Liver 2 has been identified as an outlier in the first 17 h, however this was due to technical and anatomical factors rather than perfusion factors.

A significant range of arterial flows were observed during the initial hours of perfusion. This is likely secondary to our protocol of cold-to-warm perfusion, with the perfusion starting at 25 °C before gradual rewarming to meet our temperature targets. Time-to-warm and arterial flows are closely related, with poor flows leading to an extended re-warming period and cold organs displaying very high initial vascular resistance which improve as the organ approaches normothermia. There is evidence to support the controlled oxygenated rewarming (COR) of organs (both kidney and liver) with a reduction in ischaemia reperfusion injury observed when employing this technique^19^. To further optimise our protocol, and improve the likelihood of success, COR with hypothermic perfusion prior to normothermic perfusion may be beneficial, however this is associated with increased costs and the requirement of an initial acellular oxygen carrier.

Hyperkalaemia, hypernatraemia, hyperchloraemia, and acidosis are a seemingly inevitable consequence of extended NMP. Haemolysis, cell rupture, and infusion of crystalloids are all likely major contributors to these electrolyte and acid-base imbalances. In a closed circuit, without haemofiltration, these electrolytes accumulate within a short period of time and likely influence fluids shifts within the organ which may have a detrimental impact on its function. Additionally, electrolyte imbalances lead to protein denaturation and the failure of enzymatic processes and essential cellular proteins.

Electrolyte and haematocrit changes within the circuit are most notable during the first 24 h of perfusion, especially during the period following CVVH initiation. Perfusate sodium begins at a sub-physiological level and is gradually corrected by the dialysate fluid until reaching the physiological concentrations. During this period there is also a reduction in haematocrit, possibly reflecting fluid movements from the liver into the perfusate. This providing a possible explanation for the reduction in weight observed over the course of the perfusion in the majority of livers perfused.

In vivo, the liver consumes lactate produced in peripheral organs and produces glucose as recognised in the Cori cycle. This shuttling of energy precursors is the normal physiological process^20^. This project is the first, to the best of our knowledge, to utilise exogenous lactate in the assessment of hepatocyte function, using two different methods. The first, a continuous infusion of sodium lactate solution allows a real-time quantitative measure of a liver’s lactate clearing capacity and acts as a physiologic energy substrate. This is an evolution of the current transplant viability criteria utilised and allows quantitative assessment of the organ beyond the initial 6 h. Livers 1–5 had a peak lactate clearing capacity between 20 and 35 h (median of 23 h) suggesting that this may be the optimum period for NMP allowing the liver to return to its pre-retrieval condition. No organs perfused in this study were subsequently transplanted and it is unclear whether an increased lactate clearing capacity correlates with good liver function following implantation.

The second, a lactate challenge aiming to replicate the lactate clearance observed during establishment of NMP using a bolus of sodium lactate solution. Again, this provides a quantitative assessment of the organ’s function at a specific timepoint and allows comparison between livers. During eNMP, a successful lactate challenge and clearance provides reassurance that the organ is continuing to function. The perfusate volume during the perfusion was not recorded and is a potential limitation of the study. Changes in volume may yield small but significant changes in total lactate and may impact time for lactate clearance. Future work should measure perfusate volume and will allow more accurate assessment of lactate metabolism.

With lactate metabolism and clearance forming a key component in the majority of NMP based viability criteria employed, the evidence supporting lactate clearance correlating with good hepatocyte function continues to grow as more livers are transplanted following NMP^1,4,21^. A rise in lactate has been observed in a minority of livers perfused with NMP, but even these grafts have gone on to be transplanted with good allograft function in recipients^16^. However, there is little evidence on the significance of delayed rises in lactate on liver function and allograft outcomes. Hyperlactataemia is usually attributed to hypoxia or tissue hypoperfusion (Cohen-Woods Type A lactic acidosis) but it can also reflect poor graft function (in liver transplantation), vitamin or enzyme deficiencies, as well as poisoning (Cohen-Woods Type B lactic acidosis).

Our initial extended liver perfusion attempts (A-D) all showed a progressive rise in perfusate lactate which often exceeded the upper limit of our blood gas analyser’s range ( > 20 mmol/L). This was associated with a predictable fall in pH, increase in hepatic artery resistance (with increased pressures required to maintain target flows), and a rise in perfusate transaminase levels.

During our lactate challenges (described above), we observed a prolongation in the time taken to clear lactate to <2.5 mmol/L. To combat this, we initiated a continuous infusion of glucagon at 0.1 IU/hr of glucagon via the portal circuit to inhibit glucose uptake by hepatocytes and promote glycogen mobilisation to stimulate lactate uptake and metabolism.

When examining the lactate clearing capacity of Livers 1-5 (excluding challenges augmented by glucagon infusion), we observed a low initial clearance rate which improved to a peak at 24 h suggesting improving hepatocyte metabolic function as eNMP progressed. However, lactate clearance dropped with each subsequent challenge. In Livers 4 & 5, following prolonged lactate challenges ( > 6 h), glucagon was infused in parallel to the lactate challenge. In doing so, we demonstrate that hepatocytes remain sensitive to hormones and theoretically continue to maintain normal physiological function and response, as observed in the dramatically increased lactate clearing capacity of livers upon glucagon infusion.

In Livers 4 & 5, following prolonged lactate challenges ( > 6 h), glucagon was infused in parallel to the lactate challenge. In doing so, we demonstrate that hepatocytes remain sensitive to hormones and theoretically continue to maintain normal physiological function and response, as observed in the dramatically increased lactate clearing capacity of livers upon glucagon infusion.

We observed an inverse relationship between the infusion of lactate and glucose during eNMP, with lactate infusion rates reducing over time (Spearman’s rank −0.3741, *p* < 0.0001) and glucose infusion increasing over time (Spearman’s rank 0.7445, *p* < 0.0001). On the basis of the inverse relationship between lactate and glucose infusion rates we hypothesise that a shift in metabolism occurs during machine perfusion, with livers altering their preferred energy substrate from lactate to glucose over time. It is unclear what was driving this, but glucagon infusion reversed this, suggesting that the apparatus for lactate processing remains intact.

Methylprednisolone was added to our perfusion protocol during Liver 1 following the successes of both the Zurich and Sydney groups^7,13^. We followed the guidance of the Zurich team, adding 500 mg methylprednisolone during liver connection and infusing 500 mg/24 h thereafter.

Following its inclusion, all five livers perfused were able to continue NMP in excess of 120 h with evidence of preserved hepatocyte and cholangiocyte function. We cannot identify the specific mechanism(s) by which glucocorticoids aid in liver preservation and function from these experiments, but it is likely multifactorial impacting liver function, potentially dampening ischaemia reperfusion injury, and impacting on hepatic metabolism. There have been several case reports documenting liver enzyme derangement and acute liver failure in patients with steroid insufficiency demonstrating the importance of glucocorticoids to hepatic physiology and function^22–27^.

One unexpected benefit of the addition of methylprednisolone was its vasoconstrictive benefits (affecting both arterial and portal systems). In previous attempts at eNMP, our group had experimented with noradrenaline and vasopressin infusions in an attempt to increase vascular resistance and reduce vascular flows. Noradrenaline, as expected, had a preponderance towards affecting the arterial system, with minimal impact on the venous system seemingly untouched. Vasopressin, conversely, was non-prejudiced and affected both systems equally. Both vasoconstrictive agents however were profoundly unpredictable, impacting their effective titration. Additionally, each graft demonstrated differing sensitivities to these agents, further hampering attempts at titration of these vasoactive agents with profound reduction in either arterial, portal, or both systems flow. The delay between initiation of infusion and observation of effect was hours rather than the seconds expected, and the drastic effects seen may be due to accumulation within the circuit.

Following addition of methylprednisolone, there was no requirement for vasoconstrictive agents, with this agent providing a reproducible, consistent increase in vascular resistance, facilitating the achievement of target flows using physiological pressures with relative ease.

Cholangiocyte health is essential to long-term graft function and survival. As such, there has been enormous research, predominantly in DCD grafts, focusing on differentiating grafts during machine perfusion to predict those at increased risk of ischaemic type biliary lesions (ITBL). Features predictive of ITBL include pH <7.5, low biliary bicarbonate, and biliary glucose <3 mmol/L or >10 mmol/L difference between perfusate and bile glucose^28,29^. In our study, three of the five livers perfused with the final successful eNMP protocol were donated as a consequence of brainstem death and thus at very low risk of developing ITBL if transplanted.

One inevitable consequence of perfusing donor livers in research is the need for regular biopsies to enable observation of cellular integrity. Unfortunately, this increases the risk of developing haemobilia which can disrupt and manipulate biliary blood gas results as invariably the pH is lower, and the glucose much higher in the perfusate than the bile.

Bile produced during the first 12 h of perfusion varied the most in pH, with a range between 6.96–7.81. The significance of this on long-term outcomes is unclear. Liver 5 had the lowest bile pH of 6.96 at hour 6. This liver was a severely steatotic DCD graft with a prolonged CIT (9 h 46 min), making it high risk of developing ITBL. Despite this early bile gas reading, the subsequent bile analysis over the next 160 h shows readings consistently > 7.5, with bile glucose <3 mmol/L, suggestive of preserved cholangiocyte integrity. Liver 5 also had the lowest bile production, only 723 ml in 168 h, and highlights the difficultly in assessing these organs and predicting their function and outcomes if they were to be transplanted.

Four of five livers (Livers 1-4) had ALT peaks <6000 U/L consistent with limited hepatocellular damage and potential suitability for transplantation^29^. Interestingly, ALT and AST peak at very similar times, 30 h and 24 h, respectively, and it has been suggested in the literature that this may represent the peak of the ischaemia reperfusion injury and the start of its resolution, which matches our findings of peak metabolic activity (lactate clearance) too. This hypothesis is by the Zurich team’s observation of minimal instability on reperfusion in the recipient upon implantation of a graft after 68 h of machine perfusion^11^.

A peak ALT & AST with IRI falling over time, reassures that these livers are not experiencing further injury during the extended perfusion period, and supports the notion that these livers are functioning up until the point perfusion is ceased. Liver 5 experienced the most hepatocyte damage during the initial 24–48 h of perfusion, highlighted by its peak ALT and AST values compared to the rest. This was also the only liver with a peak ALT outside the 6000 U/L cut-off designated by many transplant centres. This is not unsurprisingly given the severe steatosis, donation type, and prolonged CIT all predicting a severe IRI.

The perfusion protocol used during clinical transplantation has remained unchanged for the past five years and has been performed thousands of times with great success and safety across many centres internationally. The exploration of extended NMP has highlighted key adjustments that can be made with the perfusion protocol, without detriment to the organ, which allows extension of the perfusion time.

Several recent manuscripts have been published reporting their successes in extended NMP of human and pig livers^7,12,13^. Perfusion success varies between 40–60% and highlights the difficulty in extended perfusion and the problems that still exist in the field^7,13,14^. Our work offers the first consecutive liver perfusion series with reproducible success in extended perfusion attempts. There are several differences between protocols between our protocol, and that already published. These include the focus on physiological vascular flows (and aggressive measures to correct this), N-acetylcysteine, phosphate replacement, and sodium lactate infusion. Building on this further, we have effectively explored alternative measures of liver function, allowing the real-time assessment of hepatocyte functioning, which has become increasingly important when determining function following several days of perfusion.

Using commercially available consumables and machine perfusion devices, we have demonstrated how obstacles to running and monitoring extended NMP can be overcome. Such obstacles overcome include high levels of MetHb, adverse acid-base and electrolyte balance, suboptimal vascular tone, and frequent non-invasive metabolic and viability assessment of the liver. Our methodologies are reproducible in both DBD and DCD perfused livers and displayed evidence of preserved hepatocyte and cholangiocyte function, for up to 168 h. Our results demonstrate a reliable extended NMP methodology for utilisation in research, offering the opportunity to evaluate various disease models and expand our understanding of hepatic injury, regeneration, and repair. As a research tool its value enormous, allowing researchers to explore the pharmacology and pharmacokinetics of various drugs, develop various models of liver injury, and explore hepatic immune modulation. It also provides a platform to potentially recondition and resuscitate marginal organs, the benefit of which to a global donor shortage cannot be understated.

## Supplementary information

Supplementary Information

Description of Additional Supplementary Files

Supplementary Data

Reporting Summary

## Data Availability

The source data for Figs. 2–6 and Table 2 can be found in Supplementary Data 1. Other data generated during the current study is available from the corresponding author on reasonable request.
