# Author Correction: A reproducible extended ex-vivo normothermic machine liver perfusion protocol utilising improved nutrition and targeted vascular flows

**DOI:** 10.1038/s43856-025-01320-9

**Published:** 2025-12-30

**Authors:** George Clarke, Jingwen Mao, Angus Hann, Yiyu Fan, Amita Gupta, Anisa Nutu, Erwin Buckel Schaffner, Kayani Kayani, Nicholas Murphy, Mansoor N. Bangash, Anna L. Casey, Isla Wootton, Alexander J. Lawson, Bobby V. M. Dasari, M. Thamara P. R. Perera, Hynek Mergental, Simon C. Afford

**Affiliations:** 1 Liver Unit, Queen Elizabeth Hospital Birmingham, Birmingham, B15 2TH UK; 2 Birmingham Biomedical Research Centre, National Institute for Health Research (NIHR), University of Birmingham and University Hospitals Birmingham NHS Foundation Trust, Birmingham, B15 2TH UK; 3 Centre for Liver and Gastrointestinal Research, Institute of Immunology and Immunotherapy, University of Birmingham, Birmingham, B15 2TH UK; 4 Ochre-Bio Ltd, Oxford, UK; 5 Queen Elizabeth Hospital Birmingham, Birmingham, B15 2TH UK; 6 Intensive Care Unit, Queen Elizabeth Hospital Birmingham, Birmingham, B15 2TH UK; 7 Birmingham Acute Care Research Group, Institute of Inflammation and Ageing, University of Birmingham, Birmingham, B15 2TH UK; 8 Microbiology Department, Queen Elizabeth Hospital Birmingham, Birmingham, B15 2TH UK; 9 Clinical Biochemistry, Queen Elizabeth Hospital Birmingham, Birmingham, B15 2TH UK

**Keywords:** Physiology, Hepatology

Correction to: *Communications Medicine* 10.1038/s43856-024-00636-2, published online 24 October 2024

In the version of the article initially published, in the “Perfusate constitution” section of the Methods, the text “sodium taurocholic acid (Merck Millipore) was infused at 7.7 mg/h” should have read “sodium taurocholic acid (VWR Chemicals, USA) was infused at 140 mg/h” and has now been corrected in the HTML and PDF versions of the article.

