# Supplementary material for: A reproducible extended ex-vivo normothermic machine liver perfusion protocol utilising improved nutrition and targeted vascular flows
Source: Commun Med (Lond). 2024 Oct 24;4:214. doi: 10.1038/s43856-024-00636-2 (PMC11502869; doi:10.1038/s43856-024-00636-2)
Supplement: Supplementary file 1 — Supplementary Information [file 43856_2024_636_MOESM1_ESM.pdf]

Supplementary Table

**REAGENT or RESOURCE**	**SOURCE**
Aminoplasmal 10%	B. Braun, Germany
Actrapid	Novo Nordisk, USA
Calcium gluconate 10%	B. Braun, Germany
5% Human Albumin Solution	CSL Behring GmbH, Germany
20% Human Albumin Solution	CSL Behring GmbH, Germany
Epoprostenol 0.5 mg	GSK plc, UK
Sodium Lactate 50% in aqueous solution	VWR Chemicals, USA
Piperacillin with Tazobactam	Fresenius Kabi, Germany
Phytomenadione	Neon Healthcare Limited, UK
Acetylcysteine	Aurum Pharmaceuticals
Cernevit	Baxter, UK
Heparin	Wockhardt, UK
Methylprednisolone	Pfizer, USA
0.9% Sodium Chloride	Fresenius Kabi, Germany
Sodium Taurocholic Acid	VWR Chemicals, USA
Phosphate Polyfusor	Fresenius Kabi, Germany

Supplementary Method

MACHINE SETTINGS

Liver Assist Settings Prior to Liver Connection:

Set up Liver Assist as per Manufacturer's instruction. Starting settings as below:

FiO2 40%

Sweep Rate 2 L/min

Perfusion temperature 25 degrees Celsius

Hepatic artery pressure 50 mmHg

Portal vein pressure 8 mmHg

CVVH Settings Prior to Liver Connection:

Set up Aquarius CVVH as per Manufacturers instruction. Starting settings as below:

- Adult treatment
- Blood flow rate: 100 ml/min
- Body weight: 35 kg
- Pre-dilution: 500 ml/hour
- Post-dilution: 1000 ml/hour
- Temperature: 37 degrees Celsius

LIVER PREPARATION

1. Liver bathed in cold University of Wisconsin solution at 4℃ with crushed ice

2. Inferior vena cava dissected and cleaned, leaving both infra-hepatic and supra-hepatic ends open

3. Portal vein dissected and cleaned up to bifurcation

4. Common hepatic artery dissected and cleaned up to gastroduodenal artery

5. If present, accessory or replaced hepatic arteries anastomosed to a suitable stump.

6. Liver weight recorded (use to set target flows)

7. Coeliac trunk cannulated with a 16 French graduated suction catheter and secured with ligatures

8. Portal vein cannulated with a curved 25 French cannula (XVIVO, Sweden) and secured with ligatures

9. Gall bladder defect closed with heavy ligatures or sutured with 3/0 prolene, and cystic duct ligated

10. Common hepatic duct cannulated with 12–15 French T-tube.

11. Liver flushed with 2 litres of 5% dextrose solution (room temperature [20℃])

Liver Connection:

12. Place CVVH on ‘recirculation mode’

13. Liver transferred into the machine reservoir

14. Both hepatic artery and portal vein cannula primed with perfusion fluid and connected to the perfusion circuit

Liver Warming:

15. Incrementally increase perfusion temperature (portal vein dial) to 37℃ within 30 minutes of initiating NMLP. Aim to increase by 2-3℃ every 5 minutes.

RECOMMENDED BLOOD GAS SAMPLING

During the first hour of perfusion, please monitor blood flows carefully and take blood gas samples for analysis from hepatic artery oxygenator, portal vein oxygenator, and post-liver (pre-CVVH) ports.

PERFUSATE COMPOSITION

Initial Perfusate Composition:

5 x O-negative research-grade packed red cells (~1500 ml)

2 x 5% Human Albumin Solution 500 ml (1000 ml total)

1 x 20% Human Albumin Solution 100 ml

30 ml 4.8% sodium bicarbonate

10 000 units heparin

10 ml 10% calcium gluconate

Initially, 500 ml 5% Human Albumin Solution is infused through each oxygenator port to wet the circuit.

CVVH INITIATION:

Following set-up of Liver Assist device and addition of perfusate components, please connect Aquarius CVVH to Liver Assist.

CVVH inflow: Please attach CVVH inflow tubing to post-liver portal vein tubing.

CVVH outflow: Please attach CVVH outflow tubing to port at bottom of portal vein oxygenator.

The following drugs are added to the reservoir following CVVH cessation but prior to liver connection.

2.25 g piperacillin with tazobactam [24 ml]

500 mg methylprednisolone [24 ml]

200 mg N-acetylcysteine [1 ml]

CONTINUOUS INFUSIONS

Sodium Taurocholic Acid:

5.6 grams sodium taurocholic acid dissolved in 40 ml 0.9% sodium chloride.

Concentration = 140 mg/ml

Infusion rate = 1 ml/hour (140 mg/hour)

Aminoplasmal 10%

Add 5 ml (Aminoplasmal 10%) to a vial of Cernevit & add 2 ml to Aminoplasmal 10% 500 ml bottle

Add 1 mg phytomenadione (0.1 ml) to same Aminoplasmal 10% 500 ml bottle

Draw up into 50 ml syringe

Infusion rate = 10 ml/hour

Heparin:

25 000 units of heparin (25 ml) + 5 ml 0.9% sodium chloride in 50 ml BD syringe

Concentration = 833.3 units / ml

Rate = 1 ml / hour (833.3 IU/hour)

Infuse into circuit via Aquarius heparin insulin port.

Methylprednisolone:

Reconstitute 1 gram in 48 ml of 0.9% sodium chloride

Concentration = 20.83 mg/ml

Infusion rate = 1 ml/hour [20.83 mg/hour)

Piperacillin with Tazobactam:

Reconstitute 4.5 grams in 48 ml of 0.9% sodium chloride

Concentration = 93.75 mg/ml

Infusion rate = 1 ml / hour (93.75 mg/hour)

N-acetylcysteine:

Formulation = 2 gram/10 ml

Concentration = 200 mg/ml

Infusion rate = 1 ml / hour (200 mg/hour)

Phosphate Polyfusor:

Formulation = 50 mmol in 500 ml

Concentration = 0.1 mmol in 1 ml

Infusion rate = 10 ml/hour (1 mmol/hour)

VARIABLE INFUSIONS

Insulin:

Reconstitute 10 ml (100 units/ml) of Actrapid in 1000 ml of 0.9% Sodium Chloride

Concentration = 1 unit/ml

Initial infusion rate = 3 ml/hour (3 units/hour)

See troubleshooting for titration

50% Sodium Lactate Solution:

Concentration = 4.4 mmol/ml

Initial infusion rate = 0 ml/hour

See troubleshooting for titration

50% Dextrose:

Concentration = 0.5 grams/ml

Initial infusion rate = 0 ml/hour

See troubleshooting for titration

Epoprostenol:

10 ml solvent added to 500 mcg vial; shake gently.

Withdraw all 10 ml & add to remaining solvent (total 50 ml).

Take 10 ml & make up to 50 ml with sodium chloride 0.9% in syringe.

Concentration = 2 mcg/ml

Initial rate = 4 ml/hour (8 mcg/hour)

See troubleshooting for titration

TARGET VALUES

Hepatic Artery Flow: 0.25–0.30 ml/min /100 g

Portal Vein Flow: 0.70 –0.80 ml/min/100 g

Temperature: 36-38 degrees Celsius

pCO_2: Titrate to pH

pO_2: 12–20 kPa

pH: 7.35–7.45

Haematocrit: 20–25%

Ca^{2+}: 1.0–1.3 mmol/L

K^+: 3.0–6.0 mmol/L

Na^+: 135–160 mmol/L

Cl^-: 100 mmol/L

tHb: 7.0 g/dL

SO_2: 96–100 %

O_2Hb: 80–100 %

MetHb: ≤2%

Glucose: 4.0–8.0 mmol/L

Lactate: 2.0–2.5 mmol/L

TROUBLESHOOTING:

pO_2:

Target pO2 is between 12–20 kPa.

If pO_2 <12 kPa, increase FiO2 by 5% and repeat arterial blood gas in 15 minutes.

If pO_2 >20 kPa, decrease FiO2 by 5% and repeat arterial blood gas in 15 minutes

pH (prior to starting CVVH):

Prior to starting CVVH the target pH is >7.0.

If pH <7.0, then add 10 ml 8.4% sodium bicarbonate to the circuit and repeat arterial blood gas in 1 hour.

Maximum correction = 10 ml of sodium bicarbonate per hour

pH (CVVH in circuit):

Target pH is 7.3–7.5.

If pH <7.3, increase sweep rate by 0.5 L/min and repeat arterial blood gas in 1 hour

If pH <7.5, decrease sweep rate by 0.5 L/min and repeat arterial blood gas in 1 hour

Target Flows:

Target flows are based on liver weight and are determined prior to liver connection.

Target hepatic arterial flow is 25–30 ml/100g/min

Target portal vein flow is 75–80 ml/100g/min

Target total flow is 100–110 ml/100g/min

Target hepatic artery pressure is 50–100 mmHg

Target portal vein pressure is 5–12 mmHg

If portal vein flow is less than target flows, then increase pressure by 1 mmHg and reassess.

If portal vein flow is more than target flows, then decrease pressure by 1 mmHg and reassess.

If hepatic artery flow is less than target, then increase pressure by 5– 0 mmHg and reassess.

If hepatic artery pressure 100 mmHg and flows are less than target range, then you can assess response to epoprostenol by providing a 0.5 ml bolus, if responsive then titrate epoprostenol infusion to achieve target flows (increase in 1 ml increments, no more than 1 ml every 30 minutes).

If hepatic artery flow is more than target, reduce pressure by 5–10 mmHg and reassess.

If hepatic artery flow is more than target, and pressure is 5 mmHg, then reduce epoprostenol infusion in 1 ml increments and assess.

Achieving target flows can be difficult and may require titration of both pressures and epoprostenol infusion rates to achieve.

Haematocrit:

Target haematocrit is 20–25%

If haematocrit is <20%, add 1 unit of O-negative packed red cells to reservoir (warm in incubator at 37℃). It is recommended to remove the volume of the packed red cells from the reservoir prior to addition, but this is not essential.

O_2Hb (Oxygenated Haemoglobin):

Target is ≥80%

If <80%, ensure that SO_2 is within normal range (96–100%), if low this can be corrected by increasing the FiO2 (increase by 5%, and then reassess in 15 minutes)

If <80% and SO2 within normal range, then a perfusate exchange will need to be performed.

Replacement fluid:

3 x O-negative research-grade packed red cells

1 x 5% Human Albumin Solution (500 ml)

1 x 20% Human Albumin Solution (100 ml)

Leave replacement fluid in incubator at 37 degrees Celsius to warm prior to perfusate exchange.

Drain volume from reservoir until volume alarm starts.

Pour replacement fluid into reservoir.

Reassess arterial blood gas in an hour.

Glucose:

Target glucose is 4–8 mmol/L

If glucose <4 mmol/L, initiate a 50% dextrose infusion at 1 gram (2 ml) / hour and titrate as required.

If glucose >8 mmol/L, stop dextrose infusion

Insulin:

Insulin is titrated according to the arterial perfusate glucose following the below sliding scale

**Perfusate glucose (mmol/L)**	**Insulin Rate**
<4.0	0 IU/ hour
4.0 to <7.1	0.5 IU / hour
7.1 to <10.1	1 IU / hour
10.1 to <14	2 IU / hour
>14	3 IU / hour

Lactate:

Target lactate is 2–2.5 mmol/L

Titrate 50% sodium lactate solution to achieve these targets, starting at 1 mmol/hour and increasing.

If lactate in excess of 2.5 mmol/L then stop or reduce the rate of infusion.

Calcium:

Target is >1.0 mmol/L

If calcium <1.0 mmol/L, then add 5 ml of 10% calcium gluconate into the circuit and reassess on subsequent blood gas analysis.
