# Supplementary material for: A reproducible extended ex-vivo normothermic machine liver perfusion protocol utilising improved nutrition and targeted vascular flows
Source: Commun Med (Lond). 2024 Oct 24;4:214. doi: 10.1038/s43856-024-00636-2 (PMC11502869; doi:10.1038/s43856-024-00636-2)
Supplement: Supplementary file 2 — Description of Additional Supplementary Files [file 43856_2024_636_MOESM2_ESM.pdf]

Description of Additional Supplementary Files

File name: Supplementary data

File description: Source data for tables and graphs.
