# Supplementary material for: A reproducible extended ex-vivo normothermic machine liver perfusion protocol utilising improved nutrition and targeted vascular flows
Source: Commun Med (Lond). 2024 Oct 24;4:214. doi: 10.1038/s43856-024-00636-2 (PMC11502869; doi:10.1038/s43856-024-00636-2)
Supplement: Supplementary file 3 — Supplementary Data [file 43856_2024_636_MOESM3_ESM.pdf]

Figure 2 **Hepatic Artery Flow (ml / 100g / min)**

Time (Hours)	A	B	C	D	1	2	3	4
0.5	30.65819861	22.83057851	24.9799197	12.89473684	30.27613412	14.99005964	8.0472103	9.360189573
1	32.62124711	15.44421488	21.3654618	15.57894737	30.8678501	17.77335984	7.027896996	23.16350711
2	37.3556582	20.09297521	28.6746988	19.42105263	39.74358974	6.998011928	23.81974249	26.48104265
3	40.76212471	23.86363636	30.5220884	21.05263158	35.8974359	8.548707753	21.78111588	30.15402844
4	41.51270208	19.4214876	21.4457831	23.10526316	35.8974359	7.435387674	27.14592275	31.6943128
5	38.79907621	19.93801653	27.9518072	22.73684211	45.36489152	3.697813121	29.13090129	30.92417062
6	33.7182448	21.43595041	31.8875502	22.68421053	40.43392505	3.817097416	28.96995708	29.56161137
7	32.33256351	29.1322314	32.2088353	23.63157895	42.40631164	3.499005964	28.54077253	28.08056872
8	30.31177829	31.9214876	32.4497992	24.21052632	47.63313609	3.260437376	29.02360515	26.9549763
9	33.19861432	33.21280992	33.0923695	23.63157895	45.06903353	3.220675944	30.15021459	26.65876777
10	35.1039261	28.40909091	25.62249	23.84210526	40.9270217	3.10139165	27.7360515	30.21327014
11	31.1778291	29.08057851	37.4297189	23.47368421	42.40631164	3.339960239	27.78969957	27.13270142
12	36.83602771	29.02892562	29.7991968	22.94736842	44.9704142	4.413518887	282.9399142	22.39336493
13	34.18013857	29.64876033	43.373494	24.05263158	41.91321499	3.737574553	27.30686695	29.44312796
14	32.10161663	29.02892562	33.4136546	24.57894737	39.84220907	3.578528827	27.5751073	29.97630332
15	28.69515012	27.68595041	42.248996	24.57894737	26.13412229	5.64612326	26.23390558	27.19194313
16	31.69745958	28.51239669	41.7670683	25.05263158	12.32741617	9.065606362	27.25321888	27.72511848
17	30.65819861	27.1177686	43.7751004	25.21052632	49.80276134	2.743538767	26.7167382	27.54739336
18	27.88683603	29.23553719	30.5220884	25.47368421	42.70216963	2.624254473	25.16094421	28.02132701
19	28.11778291	26.60123967	25.7028112	25.52631579	40.13806706	21.47117296	22.74678112	28.13981043
20	26.32794457	26.13636364	42.9718876	25.94736842	42.89940828	22.62425447	24.19527897	26.83649289
21	26.38568129	27.63429752	44.1767068	24.78947368	6.114398422	22.38568588	24.1416309	26.30331754
22	26.15473441	32.12809917	16.1445783	24.10526316	8.382642998	23.69781312	23.22961373	24.76303318
23	25.2886836	29.80371901	17.4297189	24	11.83431953	22.18687873	26.39484979	24.34834123
24	23.09468822	29.28719008	50.4417671	22.89473684	9.566074951		29.50643777	26.18483412
25	24.53810624	29.8553719	47.3092369	23.31578947	37.08086785	27.15705765	15.02145923	23.93364929
26	26.90531178	29.8553719	29.3172691	23.78947368	37.17948718	20.07952286	28.91630901	32.81990521
27	30.60046189	39.5661157	48.1927711	24.05263158	30.76923077	22.70377734	26.77038627	24.34834123
28	27.42494226	36.98347107	46.9879518	23.84210526	41.81459566	21.31212724	26.7167382	27.13270142
29	26.50115473	5.268595041	50.4417671	24.42105263	37.5739645	19.3638171	26.8776824	24.34834123
30	30.02309469	35.07231405	46.5060241	23.36842105	32.84023669	18.01192843	29.39914163	24.94075829
31	29.09930716	24.53512397	36.4658635	25	30.8678501	19.64214712	28.54077253	26.48104265
32	31.06235566	45.60950413	47.9518072	24.63157895	24.75345168	18.96620278	28.70171674	25.82938389
33	33.48729792	25.10330579	48.8353414	24.57894737	34.2209073	19.40357853	29.02360515	20.31990521
34	32.15935335	21.28099174	47.3092369	24.89473684	40.82840237	21.82902584	27.14592275	24.94075829
35	30.8891455	31.45661157	46.2650602	24.42105263	33.234714	23.61829026	18.77682403	23.992891
36	31.40877598	20.04132231	37.6706827	24.89473684	34.2209073	21.78926441	28.54077253	26.30331754
37	31.40877598	14.25619835	33.0120482	25.21052632	27.81065089	11.21272366	28.43347639	26.12559242
38	31.58198614	14.77272727	37.4297189	25.05263158	31.36094675	24.45328032	28.11158798	25.29620853
39	31.23556582	19.73140496	35.502008	25.42105263	28.30374753	19.80119284	26.8776824	24.82227488
40	29.33025404	38.48140496	34.4578313	25.36842105	32.14990138	23.85685885	27.36051502	23.81516588

41	29.90762125	21.33264463	44.497992	25.52631579	28.99408284	24.49304175	28.64806867	25.29620853
42	29.56120092	21.95247934	23.6144578	24.52631579	26.23274162	29.06560636	29.29184549	26.18483412
43	29.96535797	28.66735537	26.8273092	24.84210526	23.96449704		28.86266094	22.03791469
44	31.35103926	21.43595041	24.0963855	24.89473684	32.05128205	21.98807157	26.8776824	28.02132701
45	29.04157044	19.93801653	30.1204819	25.26315789	37.5739645	20.55666004	19.25965665	25.41469194
46	30.60046189	24.89669421	44.8995984	26.15789474	29.78303748	19.96023857	18.88412017	26.18483412
47	30.25404157	38.32644628	41.7670683	25.63157895	29.48717949	17.61431412	25.26824034	27.60663507
48	28.11778291	26.49793388	40.1606426	25.15789474	30.57199211	13.1610338	29.18454936	26.77725118
49	27.59815242	40.23760331	43.2931727	26.10526316	35.50295858	8.986083499	26.98497854	34.24170616
50	28.17551963	42.40702479	43.2931727	26	41.51873767	8.946322068	27.5751073	22.86729858
51	24.65357968	40.13429752	42.4899598	26.05263158	35.79881657	15.70576541	28.21888412	25.47393365
52	25.05773672	44.78305785	44.6586345	25.94736842	38.95463511	12.08747515	30.57939914	25.82938389
53	29.04157044	45.40289256	44.8995984	25.94736842	37.77120316	15.8250497	30.472103	31.39810427
54	32.39030023	40.23760331	44.6586345	25.10526316	32.93885602	14.31411531	28.54077253	25.35545024
55	28.40646651	46.64256198	33.6546185	25.73684211	34.02366864	15.62624254	31.38412017	25.11848341
56	25	43.80165289	37.751004	25.78947368	33.82642998	15.34791252	30.15021459	26.36255924
57	27.59815242	48.08884298	37.3493976	25.68421053	31.5581854	9.622266402	30.3111588	21.38625592
58	24.76905312	43.75	30.9236948	25.10526316	33.62919132	12.80318091	31.27682403	28.02132701
59	29.44572748	46.22933884	21.686747	24.57894737	31.45956607	11.53081511	30.84763948	23.51895735
60	31.12009238	46.12603306	36.5461847	24.84210526	31.36094675	12.16699801	23.1223176	24.28909953
61	29.67667436	44.21487603	34.4578313	25.89473684	34.51676529	20.71570577	30.3111588	21.68246445
62	29.15704388	44.26652893	40.7228916	25.89473684	31.16370809	29.14512922		29.91706161
63	27.19399538	42.56198347	37.4297189	26.73684211	31.16370809	17.29622266	28.75536481	26.42180095
64	27.82909931	44.83471074	30.9236948	25.52631579	31.45956607	23.10139165	27.5751073	26.48104265
65	27.19399538	45.8677686	26.9076305	23.63157895	29.98027613	25.16898608	30.57939914	28.79146919
66	27.71362587	43.49173554	30.4417671	22.10526316	9.368836292	26.04373757	26.18025751	29.44312796
67	27.19399538	41.89049587	33.1726908	21.63157895	16.96252465	17.93240557	31.65236052	30.21327014
68	26.73210162	43.95661157	30.2008032	25.47368421	33.72781065	21.15308151	28.32618026	29.91706161
69	24.01847575	44.21487603	31.4859438	23.73684211	29.58579882	21.63021869	27.78969957	28.08056872
70	23.90300231	42.30371901	30.2811245	23.57894737	28.30374753	9.30417495	30.57939914	15.04739336
71	23.21016166	45.04132231	24.497992	23.26315789	10.84812623	23.89662028	28.16523605	24.70379147
72	25.80831409	41.32231405	28.9156627	16.52631579	37.86982249	28.50894632	29.18454936	26.59952607
73	26.27020785	44.73140496	27.5502008	23	31.5581854	28.50894632	28.54077253	28.31753555
74	25.05773672	44.31818182	25.060241	23.42105263	43.78698225	28.70775348	32.29613734	28.43601896
75	23.72979215	41.37396694	18.5542169	22.42105263	37.67258383	17.45526839	28.11158798	32.22748815
76	21.13163972	43.49173554	22.7309237	21.78947368	34.61538462	20.35785288	29.18454936	28.25829384
77	25.86605081	43.23347107	20.8835341	21.21052632	39.74358974	27.51491054	30.79399142	21.80094787
78	21.82448037	45.71280992	24.4176707	26.63157895	41.42011834	26.40159046	30.68669528	24.11137441
79	28.75288684	46.5392562	26.184739	24.68421053	35.79881657	27.87276342	33.85193133	24.76303318
80	20.4965358	40.13429752	28.1927711	17.15789474	35.30571992	28.07157058	28.48712446	26.30331754
81	26.03926097	41.52892562	27.7108434	17.73684211	36.78500986	25.64612326	31.27682403	21.56398104
82	22.11316397	3.82231405	23.6144578	17.73684211	41.91321499	19.3638171	29.77467811	26.8957346
83	25.92378753	41.6322314	27.8714859	15.63157895	44.08284024	27.95228628	28.75536481	25
84	21.30484988	39.66942149	28.1124498	22.52631579	29.09270217	28.03180915	28.21888412	26.83649289

85	24.19168591	40.39256198	36.3052209	22.21052632	32.74161736	25.80516899	30.25751073	29.38388626
86	24.82678984	40.08264463	28.1124498	23.89473684	34.12228797	27.67395626	30.74034335	34.5971564
87	23.78752887	39.10123967	37.4297189	23.42105263	35.50295858	25.12922465	27.7360515	28.61374408
88	24.82678984	43.0785124		19.21052632	44.37869822	25.00994036	30.68669528	31.6943128
89	26.55889145	41.27066116		20.10526316	37.37672584	25.16898608	30.68669528	26.8957346
	25.11547344	39.66942149			32.24852071	26.08349901	27.30686695	28.31753555
	24.07621247	42.20041322			29.8816568	25.68588469	18.88412017	30.45023697
	19.86143187	40.2892562			40.43392505	30.61630219	24.46351931	27.48815166
	18.01385681	38.8946281			33.82642998	29.86083499	27.84334764	26.8957346
	12.99076212	38.63636364			41.42011834	27.71371769	25.64377682	25.05924171
	10.79676674	40.70247934			39.44773176	28.9860835	27.89699571	24.52606635
	11.43187067	40.34090909			39.34911243	30.01988072	29.39914163	25.82938389
	10.85450346	39.5661157			31.75542406	31.57057654	32.1888412	35.84123223
	8.487297921	39.46280992			46.6469428	24.37375746	32.40343348	26.36255924
	8.140877598	36.72520661			44.08284024	28.70775348	30.36480687	24.82227488
	5.773672055	37.44834711			36.88362919	29.94035785	30.3111588	26.54028436
	5.658198614				35.70019724	28.38966203	30.472103	30.86492891
					44.9704142	30.13916501	30.20386266	29.50236967
					40.43392505	27.95228628	30.79399142	25.05924171
					50.69033531	28.3499006	30.57939914	30.03554502
					53.3530572	30.53677932	27.95064378	24.70379147
					30.8678501	28.42942346	28.5944206	25.53317536
					48.3234714	29.34393638	30.63304721	31.63507109
					28.40236686	30.09940358	28.11158798	29.56161137
					41.81459566	29.46322068	31.00858369	28.31753555
					37.5739645	29.54274354	32.027897	31.10189573
					48.3234714	29.34393638	28.96995708	27.78436019
					47.43589744	29.82107356	31.65236052	27.54739336
					47.04142012	30.09940358	25	28.55450237
					31.36094675	29.46322068	25.75107296	30.27251185
					44.47731755		24.57081545	30.62796209
					43.58974359	31.09343936	22.53218884	30.39099526
					40.9270217	30.13916501	27.03862661	30.03554502
					32.14990138	30.49701789	26.44849785	31.6943128
					37.77120316	30.21868787	28.05793991	27.42890995
					34.61538462	30.45725646	31.86695279	22.27488152
						30.29821074	28.00429185	30.86492891
						30.49701789	31.3304721	32.87914692
						31.05367793	29.61373391	31.51658768
						30.73558648	32.13519313	28.31753555
						31.72962227	30.472103	26.12559242
						29.94035785	30.20386266	30.21327014
						28.8667992	31.49141631	27.01421801
						30.53677932	22.2639485	24.76303318

	28.70171674	23.1042654
30.29821074	30.09656652	32.40521327
30.97415507	31.00858369	33.17535545
31.29224652	30.52575107	34.47867299
30.89463221	25.16094421	32.40521327
31.1332008	29.4527897	32.40521327
30.89463221	30.472103	29.85781991
32.32604374	26.8776824	28.73222749
31.61033797	23.39055794	27.72511848
29.90059642	28.21888412	28.02132701
29.94035785	29.39914163	29.38388626
32.20675944	30.79399142	28.25829384
31.61033797	27.84334764	28.13981043
32.48508946	28.75536481	28.67298578
31.80914513	31.81330472	27.78436019
32.96222664	30.36480687	27.48815166
33.00198807	31.81330472	26.65876777
30.77534791	31.92060086	24.46682464
32.80318091	32.99356223	28.96919431
32.56461233	25.26824034	19.78672986
30.33797217	33.31545064	25.82938389
32.64413519	33.58369099	26.007109
30.97415507	33.20815451	30.92417062
31.0139165	33.74463519	34.6563981
30.29821074	33.58369099	33.29383886
31.88866799	33.69098712	31.63507109
31.09343936	32.93991416	30.56872038
33.08151093	34.49570815	25.05924171
	34.44206009	32.64218009
	34.12017167	30.8056872
		21.68246445
	31.43776824	22.98578199
	32.67167382	29.73933649
	31.11587983	30.92417062
	31.00858369	24.88151659
	29.39914163	28.37677725
	29.56008584	29.62085308
	29.72103004	28.8507109
	29.88197425	27.72511848
	32.34978541	28.49526066
	32.1888412	
	31.11587983	
	30.90128755	
	33.63733906	

32.29613734
31.86695279
31.75965665
31.11587983
30.95493562
32.56437768
31.75965665
31.06223176
31.81330472
30.472103
29.93562232

5
7.685858021
8.216880939
14.81274455
18.8373393
20.1509223
20.1509223
20.96143097
22.83398547
23.25321409
24.28731135
22.91783119
24.67859139
25.12576859
25.18166574
25.54499721
25.06987144
23.61654556
23.84013415
24.70653997
24.81833426
23.64449413
23.33705981
23.19731694
24.03577418
23.67244271
23.75628843
23.56064841
24.37115707
23.67244271
23.95192845
24.03577418
24.98602571
26.215763
24.65064282
23.33705981
25.09782001
24.81833426
20.73784237
25.01397429
23.08552264
22.470654

20.12297373

23.72833985
23.84013415
24.37115707
22.77808832
23.08552264
24.76243712
23.58859698
24.37115707
25.01397429
25.40525433
25.15371716
23.95192845
19.67579653
24.14756847
22.72219117
21.29681386
24.70653997
23.28116266
20.31861375
23.11347121
24.45500279
21.9116825
23.00167691
22.63834544
19.64784796
22.38680827
20.79373952
21.49245388
21.18501956
22.16321968
23.02962549
26.1039687
23.14141979
23.47680268
23.00167691
19.36836221
22.52655115
26.07602012
21.52040246
15.67915036
24.14756847
21.18501956

22.55449972
20.51425377
24.67859139
20.82168809
24.2314142
20.79373952
25.32140861
24.53884852
22.75013974
21.99552823
21.29681386
23.02962549
24.51089994
23.8960313
23.39295696
23.02962549
22.61039687
23.95192845
25.23756288
24.06372275
23.95192845
24.79038569
23.08552264
23.61654556
20.93348239
20.73784237
20.73784237
20.98937954
23.25321409
22.88988262
24.67859139
21.15707099
23.36500838
20.65399665
20.34656233
23.92397988
23.19731694
22.55449972
23.86808273
24.76243712
21.71604248
24.79038569
21.57629961
21.82783678

18.05477921
24.76243712
22.88988262
22.38680827
22.07937395
21.63219676
22.07937395
20.45835662
21.54835103
23.33705981
25.06987144
21.15707099
22.94577977
23.36500838
24.03577418
19.89938513
21.18501956
24.31525992
23.70039128
24.67859139
24.48295137
24.06372275
22.3588597
24.65064282
24.59474567
23.02962549
24.2314142
22.19116825
19.28451649
23.39295696
24.62269424
24.65064282
23.47680268
23.33705981
24.0078256
23.81218558
23.92397988
24.67859139
24.09167132
23.58859698

Figure 2 **Hepatic Artery Resistance (mmHg/ml/min)**

Time (Hours)	A	B	C	D	1	2	3	4	5
0.5	0.11	0.2	0.23	0.3	0.21	0.23	0.57	0.54	0.29
1	0.1	0.32	0.2	0.24	0.22	0.18	0.67	0.24	0.32
2	0.09	0.23	0.15	0.21	0.13	0.46	0.2	0.19	0.18
3	0.08	0.22	0.14	0.2	0.13	0.42	0.22	0.17	0.14
4	0.08	0.24	0.21	0.18	0.23	0.46	0.17	0.17	0.13
5	0.09	0.24	0.16	0.18	0.14	1.03	0.17	0.16	0.12
6	0.1	0.23	0.13	0.18	0.15	1.07	0.17	0.17	0.12
7	0.1	0.17	0.14	0.18	0.19	1.05	0.16	0.18	0.11
8	0.11	0.15	0.12	0.18	0.17	1.22	0.17	0.18	0.11
9	0.12	0.12	0.12	0.18	0.16	1.17	0.16	0.18	0.11
10	0.11	0.12	0.29	0.18	0.18	1.25	0.17	0.17	0.11
11		0.12	0.12	0.18	0.18	1.12	0.17	0.18	0.1
12	0.15	0.1	0.15	0.18	0.18	1.07	0.17	0.23	0.1
13	0.11	0.1	0.1	0.17	0.19	1.03	0.18	0.17	0.1
14	0.11	0.09	0.13	0.17	0.18	0.99	0.17	0.17	0.1
15	0.1	0.08	0.1	0.17	0.3	0.72	0.17	0.17	0.1
16	0.12	0.08	0.1	0.17	0.88	0.42	0.18	0.17	0.1
17	0.12	0.07	0.1	0.17	0.13	1.33	0.17	0.17	0.1
18	0.1	0.06	0.14	0.17	0.13	1.41	0.18	0.17	0.1
19	0.12	0.07	0.2	0.16	0.14	0.14	0.21	0.17	0.1
20	0.13	0.07	0.09	0.17	0.12	0.14	0.19	0.17	0.1
21	0.14	0.06	0.09	0.17	1.01	0.14	0.2	0.18	0.11
22	0.13	0.06	0.29	0.16	0.9	0.13	0.22	0.17	0.1
23	0.14	0.06	0.25	0.16	0.91	0.15	0.19	0.2	0.1
24	0.15	0.07	0.14	0.19	0.4		0.16	0.17	0.1
25	0.17	0.06	0.08	0.17	0.23	0.12	0.32	0.19	0.1
26	0.16	0.07	0.14	0.16	0.23	0.19	0.16	0.13	0.1
27	0.14	0.05	0.09	0.16	0.28	0.15	0.18	0.19	0.1
28	0.12	0.05	0.09	0.16	0.21	0.17	0.17	0.19	0.1
29	0.13	0.64	0.09	0.17	0.24	0.18	0.17	0.2	0.1
30	0.15	0.07	0.09	0.16	0.25	0.19	0.16	0.18	0.1
31	0.13	0.12	0.11	0.16	0.29	0.17	0.16	0.18	0.1
32	0.13	0.05	0.09	0.16	0.28	0.19	0.16	0.17	0.09
33	0.13	0.08	0.09	0.15	0.2	0.19	0.16	0.23	0.1
34	0.12	0.22	0.09	0.15	0.18	0.16	0.17	0.19	0.11
35	0.13	0.1	0.09	0.16	0.21	0.14	0.28	0.21	0.1
36	0.12	0.12	0.12	0.16	0.2	0.16	0.17	0.18	0.1
37	0.11	0.22	0.13	0.15	0.24	0.28	0.17	0.19	0.12
38	0.11	0.2	0.12	0.15	0.19	0.16	0.17	0.19	0.1
39	0.11	0.18	0.13	0.14	0.21	0.17	0.17	0.2	0.11
40	0.11	0.06	0.12	0.15	0.2	0.16	0.18	0.21	0.11

41	0.12	0.15	0.09	0.15	0.22	0.15	0.16	0.21	0.12
42	0.11	0.13	0.27	0.16	0.26	0.12	0.16	0.16	
43	0.12	0.08	0.28	0.16	0.31		0.17	0.38	0.1
44	0.11	0.11	0.32	0.15	0.27	0.13	0.17	0.18	0.1
45	0.11	0.12	0.22	0.14	0.23	0.13	0.29	0.2	0.1
46	0.11	0.09	0.15	0.15	0.22	0.15	0.24	0.19	0.11
47	0.11	0.05	0.1	0.15	0.24	0.15	0.19	0.18	0.11
48	0.11	0.06	0.11	0.15	0.27	0.22	0.17	0.19	0.11
49	0.11	0.05	0.1	0.15	0.22	0.37	0.17	0.13	0.09
50	0.11	0.04	0.1	0.15	0.19	0.42	0.17	0.22	0.09
51	0.12	0.04	0.1	0.16	0.2	0.21	0.16	0.2	0.09
52	0.14	0.04	0.1	0.15	0.17	0.28	0.17	0.19	0.09
53	0.18	0.04	0.09	0.15	0.18	0.22	0.16	0.16	0.09
54	0.13	0.04	0.1	0.15	0.2	0.23	0.16	0.18	0.09
55	0.11	0.04	0.14	0.15	0.21	0.21	0.15	0.19	0.11
56	0.12	0.04	0.11	0.15	0.22	0.22	0.15	0.18	0.09
57	0.14	0.04	0.11	0.15	0.25	0.36	0.15	0.22	0.09
58	0.14	0.04	0.14	0.15	0.23	0.27	0.15	0.18	0.1
59	0.16	0.04	0.21	0.15	0.23	0.3	0.15	0.22	0.09
60	0.15	0.04	0.11	0.15	0.26	0.31	0.21	0.21	0.09
61	0.12	0.04	0.12	0.15	0.22	0.18	0.14	0.23	0.12
62	0.12	0.04	0.11	0.15	0.25	0.11		0.17	0.11
63	0.11	0.04	0.12	0.15	0.23	0.22	0.15	0.2	0.1
64	0.12	0.04	0.15	0.15	0.23	0.16	0.15	0.2	0.12
65	0.12	0.04	0.19	0.15	0.27	0.14	0.15	0.19	0.11
66	0.13	0.04	0.17	0.16	1.15	0.13	0.18	0.18	0.11
67	0.12	0.04	0.14	0.21	0.53	0.19	0.15	0.15	0.14
68	0.12	0.05	0.15	0.17	0.25	0.16	0.15	0.13	0.11
69	0.13	0.04	0.16	0.17	0.26	0.16	0.16	0.17	0.12
70	0.14	0.04	0.16	0.16	0.27	0.39	0.14	0.34	0.12
71	0.15	0.04	0.16	0.17	1.13	0.15	0.16	0.17	0.12
72	0.14	0.05	0.16	0.27	0.22	0.12	0.15	0.18	0.12
73	0.14	0.05	0.16	0.17	0.24	0.11	0.15	0.17	0.11
74	0.14	0.05	0.19	0.16	0.16	0.11	0.14	0.17	0.1
75	0.15	0.05	0.28	0.18	0.19	0.2	0.16	0.14	0.1
76	0.19	0.04	0.23	0.19	0.19	0.15	0.15	0.16	0.11
77	0.21	0.04	0.25	0.23	0.17	0.12	0.14	0.22	0.11
78	0.17	0.04	0.22	0.18	0.18	0.12	0.14	0.21	0.13
79	0.21	0.04	0.18	0.16	0.23	0.11	0.12	0.2	0.11
80	0.17	0.05	0.18	0.29	0.22	0.11	0.17	0.2	0.09
81	0.2	0.05	0.18	0.26	0.19	0.12	0.14	0.21	0.12
82	0.16	0.05	0.23	0.24	0.16	0.17	0.14	0.18	0.16
83	0.17	0.05	0.18	0.31	0.16	0.12	0.15	0.21	0.1
84	0.15	0.05	0.18	0.17	0.22	0.11	0.15	0.2	0.12

85	0.17	0.05	0.14	0.18	0.2	0.12	0.15	0.17	0.12
86	0.17	0.05	0.19	0.19	0.19	0.12	0.15	0.13	0.11
87	0.16	0.05	0.14	0.19	0.19	0.13	0.15	0.19	0.11
88	0.18	0.05		0.23	0.13	0.14	0.14	0.16	0.13
89	0.18	0.05		0.22	0.13	0.14	0.14	0.17	0.11
90	0.18	0.05			0.17	0.13	0.15	0.15	0.13
91	0.18	0.05			0.25	0.12	0.22	0.14	0.1
92	0.19	0.05			0.16	0.09	0.18	0.15	0.11
93	0.26	0.05			0.19	0.09	0.14	0.16	0.11
94	0.3	0.05			0.15	0.09	0.17	0.17	0.12
95	0.45	0.05			0.14	0.09	0.16	0.17	0.13
96	0.54	0.05			0.2	0.09	0.14	0.18	0.11
97	0.54	0.05			0.19	0.08	0.13	0.12	0.11
98	0.58	0.05			0.12	0.11	0.13	0.15	0.1
99	0.59	0.05			0.12	0.09	0.14	0.19	0.1
100	0.54	0.05			0.13	0.08	0.04	0.18	0.1
	0.56				0.14	0.08	0.14	0.15	0.12
	0.57				0.1	0.08	0.13	0.15	0.11
					0.18	0.08	0.13	0.18	0.1
					0.1	0.08	0.13	0.16	0.11
					0.1	0.08	0.14	0.2	0.1
					0.17	0.09	0.14	0.19	0.09
					0.1	0.08	0.13	0.16	0.09
					0.29	0.08	0.15	0.15	0.11
					0.17	0.08	0.13	0.15	0.13
					0.18	0.09	0.13	0.15	0.13
					0.12	0.09	0.15	0.17	0.13
					0.11	0.08	0.13	0.17	0.13
					0.11	0.08	0.16	0.16	0.12
					0.15	0.09	0.16	0.15	0.12
					0.1		0.18	0.15	0.11
					0.11	0.08	0.21	0.15	0.11
					0.12	0.08	0.18	0.15	0.11
					0.17	0.08	0.17	0.14	0.12
					0.16	0.08	0.13	0.17	0.12
					0.19	0.08	0.13	0.23	0.11
						0.08	0.15	0.16	0.12
						0.08	0.13	0.14	0.12
						0.08	0.14	0.14	0.1
						0.08	0.13	0.15	0.1
						0.08	0.14	0.17	0.12
						0.08	0.14	0.15	0.1
						0.08	0.132	0.17	0.12
						0.08	0.26	0.18	0.12

	0.14	0.2	0.15
0.08	0.15	0.15	0.09
0.08	0.14	0.14	0.12
0.08	0.14	0.13	0.11
0.08	0.25	0.13	0.12
0.08	0.13	0.14	0.12
0.08	0.14	0.13	0.13
0.08	0.15	0.14	0.14
0.08	0.18	0.14	0.13
0.08	0.15	0.15	0.11
0.08	0.14	0.14	0.11
0.07	0.14	0.13	0.13
0.08	0.15	0.13	0.12
0.08	0.15	0.13	0.1
0.08	0.13	0.13	0.09
0.08	0.13	0.14	0.12
0.08	0.13	0.15	0.13
0.08	0.16	0.18	0.09
0.08	0.13	0.18	0.1
0.07	0.17	0.24	0.09
0.08	0.12	0.18	0.1
0.08	0.12	0.19	0.09
0.08	0.13	0.16	0.09
0.08	0.13	0.14	0.1
0.07	0.12	0.14	0.09
0.07	0.12	0.14	0.09
0.08	0.12	0.15	0.09
0.07	0.12	0.18	0.11
	0.12	0.13	0.14
	0.12	0.13	0.09
		0.23	0.09
	0.12	0.23	0.1
	0.12	0.15	0.1
	0.12	0.13	0.1
	0.12	0.19	0.09
	0.13	0.12	0.1
	0.13	0.12	0.1
	0.13	0.13	0.09
	0.13	0.14	0.09
	0.12	0.13	0.09
	0.12		
	0.12		
	0.12		
	0.12		

0.12
0.12
0.12
0.13
0.12
0.12
0.12
0.12
0.12
0.12
0.13

Figure 2 **Portal Vein Flow (ml / 100g / min)**

Time (Hours)	A	B	C	D	1	2	3	4
0.5	68.70669746	75.92975207	97.188755	68.94736842	93.68836292	49.30417495	64.3776824	72.27488152
1	69.86143187	75.41322314	112.449799	75.26315789	71.00591716	68.38966203	75.10729614	67.53554502
2	71.01616628	85.22727273	119.678715	82.10526316	96.6469428	73.55864811	79.93562232	79.97630332
3	71.01616628	77.47933884	117.269076	79.47368421	85.79881657	79.12524851	84.7639485	91.23222749
4	75.63510393	76.96280992	111.646586	72.63157895	86.78500986	74.75149105	88.5193133	94.78672986
5	75.63510393	76.44628099	117.269076	72.63157895	93.68836292	71.96819085	78.32618026	87.44075829
6	77.36720554	76.44628099	127.710843	72.10526316	101.5779093	73.55864811	79.93562232	85.90047393
7	79.67667436	84.19421488	128.514056	75.78947368	86.78500986	73.55864811	80.472103	81.16113744
8	78.52193995	83.16115702	126.907631	73.15789474	104.5364892	73.55864811	80.472103	80.56872038
9	74.48036952	81.09504132	124.497992	72.10526316	97.63313609	73.1610338	79.93562232	81.7535545
10	76.78983834	73.86363636	127.710843	72.63157895	97.63313609	71.17296223	82.08154506	81.7535545
11	72.7482679	71.79752066	126.907631	74.21052632	100.591716	69.98011928	81.54506438	84.12322275
12	73.3256351	71.28099174	135.742972	71.05263158	85.79881657	68.38966203	84.7639485	84.12322275
13	74.48036952	75.41322314	133.333333	69.47368421	85.79881657	68.38966203	86.37339056	85.90047393
14	77.94457275	77.47933884	135.742972	70	88.75739645	71.57057654	85.83690987	86.492891
15	79.09930716	78.51239669	135.742972	71.05263158	94.67455621	72.36580517	85.83690987	86.492891
16	78.52193995	78.51239669	139.759036	72.63157895	86.78500986	77.93240557	87.44635193	87.67772512
17	79.09930716	76.96280992	139.759036	72.63157895	113.4122288	79.52286282	0.086909871	86.492891
18	74.48036952	72.83057851	139.759036	73.15789474	85.79881657	76.34194831	80.472103	85.90047393
19	75.05773672	72.31404959	126.104418	72.63157895	85.79881657	69.58250497	78.86266094	86.492891
20	76.78983834	71.28099174	130.923695	71.57894737	89.74358974	65.20874751	77.78969957	87.08530806
21	77.94457275	72.31404959	130.120482	72.63157895	88.75739645	69.18489066	78.86266094	85.30805687
22	79.09930716	70.76446281	130.120482	73.15789474	109.4674556	76.73956262	79.93562232	85.30805687
23	79.09930716	72.31404959	127.710843	78.42105263	111.4398422	77.53479125	78.86266094	84.12322275
24	79.67667436	72.31404959	124.497992	78.42105263	107.495069		76.7167382	90.04739336
25	78.52193995	69.21487603	130.923695	76.31578947	109.4674556	71.96819085	78.86266094	88.86255924
26	71.01616628	71.28099174	129.317269	72.63157895	115.3846154	74.75149105	85.30042918	85.90047393
27	79.09930716	70.76446281	121.285141	72.63157895	110.4536489	76.34194831	77.78969957	87.08530806
28	83.7182448	70.76446281	122.088353	77.36842105	99.60552268	77.13717694	78.32618026	90.04739336
29	84.87297921	73.86363636	119.678715	73.68421053	107.495069	77.53479125	85.83690987	87.67772512
30	86.02771363	82.6446281	116.465863	73.15789474	109.4674556	75.944334	77.78969957	89.4549763
31	84.87297921	88.84297521	121.285141	73.68421053	102.5641026	74.35387674	77.25321888	88.86255924
32	86.60508083	79.54545455	120.481928	73.68421053	103.5502959	78.33001988	77.25321888	91.23222749
33	79.67667436	80.5785124	118.072289	74.73684211	99.60552268	79.12524851	78.86266094	87.67772512
34	77.36720554	73.86363636	86.746988	74.21052632	101.5779093	73.1610338	85.30042918	84.71563981
35	78.52193995	77.47933884	107.630522	74.21052632	97.63313609	77.93240557	88.5193133	84.12322275
36	81.98614319	73.86363636	122.088353	72.63157895	94.67455621	79.52286282	86.37339056	82.93838863
37	83.1408776	77.47933884	95.5823293	72.10526316	95.66074951	67.99204771	86.90987124	84.12322275
38	81.98614319	73.86363636	90.7630522	72.10526316	89.74358974	71.57057654	86.90987124	85.30805687
39	79.09930716	73.86363636	92.3694779	71.05263158	88.75739645	81.11332008	79.39914163	85.90047393
40	79.09930716	73.34710744	77.9116466	74.21052632	85.79881657	71.57057654	78.86266094	79.97630332

41	72.7482679	73.34710744	79.5180723	74.21052632	87.77120316	79.92047714	79.93562232	82.93838863
42	71.01616628	69.73140496	44.9799197	73.15789474	90.72978304	71.17296223	79.93562232	85.30805687
43	70.43879908	67.66528926	68.2730924	72.63157895	86.78500986		80.472103	84.12322275
44	71.59353349	67.66528926	84.3373494	73.15789474	97.63313609	76.34194831	81.00858369	84.71563981
45	72.7482679	70.24793388	74.6987952	74.73684211	100.591716	77.13717694	83.15450644	85.30805687
46	73.90300231	68.18181818	94.7791165	74.21052632	105.5226824	75.944334	80.472103	85.30805687
47	72.17090069	70.24793388	97.188755	73.68421053	105.5226824	73.55864811	85.83690987	85.90047393
48	71.01616628	67.14876033	58.6345382	76.84210526	106.5088757	71.17296223	84.22746781	85.90047393
49	71.01616628	66.6322314	92.3694779	73.15789474	91.71597633	67.99204771	83.15450644	88.86255924
50		65.59917355	89.9598394	98.94736842	92.70216963	64.41351889	81.54506438	84.12322275
51	68.70669746	69.73140496	93.1726908	104.2105263	80.8678501	64.01590457	80.472103	90.04739336
52	83.7182448	73.34710744	92.3694779	105.2631579	108.4812623	65.60636183	81.00858369	86.492891
53	82.56351039	72.83057851	89.1566265	104.7368421	93.68836292	76.34194831	79.93562232	88.86255924
54	84.87297921	73.86363636	50.6024096	105.7894737	94.67455621	66.00397614	80.472103	87.67772512
55	84.29561201	75.41322314	39.3574297	106.8421053	91.71597633	64.41351889	82.08154506	88.27014218
56	86.02771363	75.41322314	86.746988	111.0526316	92.70216963	75.54671968	82.08154506	87.08530806
57	83.7182448	74.89669421	89.9598394	112.6315789	94.67455621	70.3777336	80.472103	88.86255924
58	81.98614319	76.44628099	65.8634538	113.1578947	92.70216963	66.79920477	82.08154506	85.90047393
59	81.40877598	75.41322314	85.1405622	113.6842105	104.5364892	68.78727634	82.08154506	85.90047393
60	78.52193995	74.89669421	85.1405622	113.6842105	106.5088757	67.99204771	83.15450644	87.67772512
61	77.94457275	74.89669421	85.9437751	114.7368421	101.5779093	73.55864811	81.54506438	83.53080569
62	76.78983834	74.38016529	69.0763052	120.5263158	104.5364892	74.75149105		84.71563981
63	75.63510393	73.86363636	92.3694779	86.31578947	104.5364892	72.36580517	78.32618026	87.08530806
64	74.48036952	77.99586777	86.746988	93.15789474	103.5502959	78.72763419	79.39914163	87.08530806
65	73.3256351	75.92975207	77.1084337	95.78947368	106.5088757	76.73956262	77.78969957	88.27014218
66	73.90300231	75.92975207	78.7148594	98.42105263	100.591716	75.944334	78.32618026	88.86255924
67	71.59353349	75.41322314	92.3694779	96.84210526	101.5779093	75.14910537	77.25321888	90.63981043
68	72.17090069	75.92975207	75.502008	97.36842105	104.5364892	75.54671968	74.57081545	93.60189573
69	70.43879908	76.44628099	84.3373494	97.36842105	88.75739645	75.14910537	84.7639485	93.60189573
70	71.01616628	74.38016529	77.9116466	97.89473684	86.78500986	70.3777336	84.7639485	93.60189573
71	71.59353349	72.83057851	84.3373494	70	103.5502959	68.78727634	84.7639485	91.82464455
72	78.52193995	72.83057851	85.9437751	81.57894737	104.5364892	71.96819085	82.08154506	88.86255924
73	77.94457275	71.28099174	84.3373494	92.63157895	104.5364892	66.40159046	82.61802575	84.12322275
74	75.05773672	72.31404959	81.124498	95.26315789	89.74358974	69.18489066	83.69098712	90.63981043
75	66.97459584	71.79752066	76.3052209	94.73684211	89.74358974	69.98011928	90.12875536	91.23222749
76	69.28406467	71.79752066	86.746988	62.10526316	83.82642998	71.96819085	78.32618026	87.67772512
77	77.36720554	72.83057851	86.746988	73.15789474	83.82642998	72.36580517	75.64377682	84.71563981
78	72.7482679	73.34710744	86.746988	78.42105263	81.85404339	71.96819085	86.37339056	84.71563981
79	77.36720554	73.86363636	98.7951807	81.57894737	85.79881657	71.17296223	84.7639485	84.12322275
80	68.70669746	73.86363636	109.236948	72.10526316	84.81262327	66.79920477	85.83690987	88.27014218
81	76.78983834	73.86363636	85.9437751	67.36842105	80.8678501	71.96819085	85.30042918	85.90047393
82	75.05773672	73.34710744	87.5502008	78.94736842	83.82642998	72.76341948	86.37339056	85.30805687
83	75.63510393	70.76446281	92.3694779	88.42105263	102.5641026	73.55864811	85.83690987	83.53080569
84	75.63510393	70.76446281	77.1084337	82.63157895	96.6469428	70.77534791	86.37339056	83.53080569

85	75.63510393	68.69834711	72.2891566	85.26315789	101.5779093	73.1610338	86.90987124	81.7535545
86	73.90300231	69.73140496	97.9919679	97.36842105	100.591716	67.99204771	86.37339056	83.53080569
87	83.7182448	66.6322314	94.7791165	83.15789474	102.5641026	74.35387674	86.37339056	84.12322275
88	82.56351039	64.04958678		57.36842105	98.61932939	65.60636183	84.7639485	84.12322275
89	80.83140878	67.66528926		79.47368421	101.5779093	74.35387674	84.22746781	87.67772512
90	80.25404157	65.08264463			102.5641026	74.35387674	85.83690987	87.67772512
91	79.09930716	59.91735537			95.66074951	74.75149105	78.86266094	87.67772512
92	81.98614319	75.92975207			93.68836292	66.79920477	83.15450644	86.492891
93	83.7182448	72.31404959			98.61932939	64.01590457	85.30042918	88.27014218
94	85.45034642	72.31404959			92.70216963	70.77534791	86.37339056	88.86255924
95	85.45034642	70.76446281			95.66074951	69.18489066	85.83690987	89.4549763
96	75.05773672	69.73140496			82.84023669	69.58250497	85.30042918	90.04739336
97	73.3256351	68.69834711			88.75739645	67.5944334	86.90987124	89.4549763
98	68.12933025	67.04545455			90.72978304	68.38966203	89.05579399	88.86255924
99	51.9630485	66.11570248			88.75739645	69.18489066	84.22746781	90.04739336
100	69.86143187	67.14876033			98.61932939	70.3777336	85.83690987	88.86255924
101	71.59353349				96.6469428	67.99204771	80.472103	89.4549763
102					89.74358974	67.5944334	80.472103	87.08530806
103					76.92307692	62.82306163	81.54506438	88.27014218
104					88.75739645	63.61829026	83.15450644	83.53080569
105					89.74358974	69.58250497	84.7639485	83.53080569
106					87.77120316	74.35387674	84.22746781	87.67772512
107					81.85404339	68.38966203	83.69098712	86.492891
108					88.75739645	71.96819085	83.69098712	87.08530806
109					97.63313609	69.58250497	83.69098712	79.97630332
110					84.81262327	74.75149105	84.22746781	78.79146919
111					87.77120316	69.98011928	81.54506438	87.08530806
112					85.79881657	76.34194831	82.61802575	88.86255924
113					91.71597633	71.17296223	82.08154506	88.27014218
114					95.66074951	74.35387674	82.61802575	88.86255924
115					101.5779093		81.00858369	87.67772512
116					97.63313609	71.96819085	83.69098712	90.63981043
117					85.79881657	76.34194831	84.7639485	91.82464455
118					83.82642998	74.75149105	83.15450644	84.71563981
119					77.90927022	68.78727634	82.08154506	94.78672986
120					91.71597633	75.14910537	84.22746781	85.30805687
121						65.20874751	85.83690987	93.00947867
122						63.22067594	84.7639485	93.00947867
123						66.00397614	85.83690987	95.37914692
124						67.5944334	85.83690987	94.1943128
125						65.60636183	85.83690987	91.23222749
126						62.42544732	85.30042918	91.23222749
127						62.42544732	84.22746781	90.63981043
128						61.23260437	84.22746781	81.16113744

129		85.30042918	87.08530806
130	66.79920477	84.7639485	85.30805687
131	67.5944334	85.30042918	87.08530806
132	67.19681909	85.30042918	88.27014218
133	70.3777336	84.22746781	90.63981043
134	67.5944334	83.69098712	82.93838863
135	68.78727634	85.30042918	80.56872038
136	68.78727634	82.08154506	77.60663507
137	66.00397614	86.90987124	83.53080569
138	66.40159046	86.90987124	95.37914692
139	67.99204771	87.44635193	82.34597156
140	61.23260437	88.5193133	82.93838863
141	73.95626243	87.98283262	81.16113744
142	63.22067594	88.5193133	85.90047393
143	66.40159046	87.98283262	91.23222749
144	65.60636183	88.5193133	91.23222749
145	65.20874751	88.5193133	84.71563981
146	67.5944334	89.05579399	87.67772512
147	67.99204771	89.05579399	88.86255924
148	68.38966203	87.98283262	88.27014218
149	63.22067594	87.44635193	84.12322275
150	63.61829026	88.5193133	93.00947867
151	65.60636183	89.59227468	94.1943128
152	67.19681909	89.59227468	84.71563981
153	72.36580517	89.05579399	86.492891
154	77.13717694	89.59227468	85.90047393
155	76.34194831	89.05579399	87.08530806
156	66.79920477	90.12875536	88.27014218
157		90.12875536	83.53080569
158		90.66523605	85.90047393
159			91.23222749
160		76.18025751	80.56872038
161		76.18025751	79.97630332
162		90.12875536	79.38388626
163		91.20171674	88.86255924
164		86.37339056	84.71563981
165		87.44635193	92.41706161
166		87.44635193	92.41706161
167		87.44635193	92.41706161
168		87.44635193	93.60189573
169		87.98283262	
170		87.98283262	
171		86.37339056	
172		87.44635193	

173	87.44635193
174	87.98283262
175	86.90987124
176	89.05579399
177	89.59227468
178	88.5193133
179	87.98283262
180	87.98283262
181	87.44635193
182	86.37339056
183	86.37339056

5
6.707657909
33.2588038
42.48183343
46.39463387
49.46897708
51.70486305
55.05869201
56.73560648
57.57406372
61.20737842
61.76634992
64.00223589
64.28172163
65.39966462
62.04583566
58.69200671
60.08943544
62.8842929
61.20737842
60.08943544
56.73560648
57.01509223
62.8842929
62.60480715
62.8842929
63.44326439
63.44326439
61.48686417
61.76634992
61.48686417
60.64840693
61.76634992
61.76634992
59.2509782
59.2509782
59.2509782
60.08943544
60.92789268
60.36892119
60.64840693
60.92789268

60.64840693

62.04583566
62.32532141
64.56120738
63.72275014
64.84069312
61.76634992
60.36892119
57.57406372
58.41252096
60.36892119
60.92789268
61.20737842
60.92789268
61.48686417
60.36892119
59.2509782
62.8842929
57.01509223
58.41252096
56.17663499
56.45612074
61.48686417
62.04583566
56.45612074
64.00223589
55.89714925
58.13303522
60.36892119
62.8842929
63.72275014
68.47400783
57.01509223
61.48686417
62.60480715
64.28172163
55.33817775
56.73560648
62.60480715
57.01509223
55.89714925
55.89714925
58.13303522

59.2509782
65.95863611
59.2509782
58.97149245
58.13303522
57.85354947
60.64840693
60.36892119
62.04583566
57.85354947
57.57406372
59.53046395
58.41252096
59.2509782
57.29457798
52.82280604
62.60480715
65.67915036
65.67915036
63.44326439
65.67915036
59.53046395
58.13303522
65.95863611
70.43040805
64.00223589
56.17663499
65.39966462
63.16377865
65.39966462
59.53046395
55.89714925
57.85354947
62.60480715
63.16377865
63.72275014
56.73560648
55.89714925
58.41252096
56.73560648
58.13303522
58.69200671
57.29457798
57.01509223

59.80994969
62.04583566
59.2509782
56.17663499
62.04583566
65.12017887
64.00223589
61.20737842
61.20737842
61.20737842
65.67915036
68.19452208
67.35606484
59.53046395
58.69200671
66.5176076
72.38680827
61.76634992
55.89714925
51.42537731
57.85354947
60.08943544
55.89714925
59.80994969
59.2509782
58.69200671
61.48686417
57.29457798
63.16377865
53.10229178
58.69200671
64.56120738
56.45612074
63.44326439
65.95863611
64.56120738
64.56120738
60.36892119
56.73560648
70.7098938

Figure 2 **Portal Vein Resistance (mmHg/ml/min)**

Time (Hours)	A	B	C	D	1	2	3	4	5
0.5	7.26	5.28	5.97	6.58	9.31	9.34	8.52	9.12	51.76
1	7.04	5.56	4.24	5.67	9.9	5.86	5.89	9.39	10.35
2	6.96	5.9	3.34	5.63	7.83	4.68	6.08	8.09	9.06
3	6.96	5.92	3.4	4.67	7.03	4.84	6.22	6.59	8.3
4	6.16	5.5	4.07	4.42	9.01	4.56	6.1	6.83	7.48
5	6.26	5.4	4.05	5	6.15	4.39	5.57	6.18	7.07
6	6.6	5.51	3.64	5	6.31	4.25	5.97	6.47	6.94
7	6.74	4.95	3.72	4.81	5.78	4.13	5.43	6.51	6.73
8	5.97	5.11	3.22	4.93	5.14	4.23	5.43	7.15	6.6
9	6.98	5.13	3.75	5.09	5.21	4.3	5.42	7.14	6.28
10	6.66	5.44	3.76	4.97	5.14	4.21	5.7	7.11	6.22
11		5.07	3.66	4.31	5.63	4.33	5.71	6.36	6.04
12	6.41	5.63	3.36	4.63	6.22	4.35	5.06	6.78	5.69
13	6.94	4.78	3.51	5.23	6.33	3.57	5.07	6.7	5.9
14	6.92	5.26	3.4	5.18	6.23	3.88	5.37	6.52	5.84
15	5.99	5.13	3.46	5.11	5.88	4.65	5.02	6.75	6.02
16	6.52	5.06	3.32	4.89	6.43	4.52	4.96	6.2	6.18
17	6.5	4.35	2.95	5.08	6.75	4.3	5.38	6.23	5.43
18	5.87	4.89	3.33	4.8	5.84	4.42	5.28	6.27	5.47
19	5.58	4.84	3.72	4.97	5.55	5.09	5.13	6.17	5.59
20	5.61	4.91	3.52	4.92	6.29	4.72	5.36	6.17	6.29
21	5.36	4.94	3.58	5.01	3.96	4.76	5.45	6.4	5.85
22	5.39	4.85	3.12	4.96	3.37	4.55	5.34	6.28	5.78
23	5.24	4.5	3.68	4.7	2.35	4.5	5.53	7.01	5.81
24	5.6	4.85	3.75	4.57	3.48		5.52	6.51	6.14
25	5.27	5.01	3.57	4.67	3.34	4.84	4.72	7.04	5.78
26	5.77	4.97	3.62	4.77	3.31	4.22	5.07	5.89	5.87
27	5.02	4.83	3.34	4.98	2.68	4.19	5.35	7.28	5.96
28		5.01	3.43	4.36	3.11	4.43	4.86	6.85	6.01
29		5.54	3.36	4.84	2.77	4.42	5.46	7.12	5.91
30	5.51	4.96	4.08	4.84	2.64	4.16	5.42	6.71	5.98
31	5.38	4.6	3.34	4.91	3.55	4.62	5.34	7.19	5.9
32	5.65	4.56	3.32	4.87	3.72	4.5	5.43	7.01	6.25
33	5.31	4.99	4.05	4.27	3.81	4.45	5.29	6.12	6.35
34	5.73	5.06	5.01	4.75	3.82	5.17	5.5	6.82	6.2
35	5.81	5.24	4.37	4.78	3.74	4.49	4.83	7.08	6.53
36	5.6	5.57	3.7	4.8	3.74	4.43	5.28	6.99	6.47
37	5.54	4.69	4.31	4.82	3.85	4.31	5.37	7	5.98
38	5.46	4.89	4.49	4.76	4.15	4.48	4.87	6.75	5.97
39	5.08	5.56	5.07	4.95	4.05	4.25	5.27	6.22	6.05
40	5.77	5.6	5.4	4.34	3.87	4.43	5.18	7.09	6.29

41	5.73	5.59	5.8	4.22	4.26	4.03	5.35	6.43	6.27
42	4.9	5.96	11.27	4.32	4.27	4.35	5.04	6.76	
43	4.91	5.98	11.4	4.78	4.46		5.11	6.8	6.27
44	5.05	5.35	9.47	4.91	2.98	4.56	5.17	6.94	6.19
45	5.53	5.54	6.94	4.2	3.68	4.46	4.9	6.21	5.92
46	5.52	5.54	5.9	4.32	2.85	4.6	5.17	6.83	6.04
47	4.72	5.92	5.04	4.37	3.52	4.62	4.84	6.15	5.99
48	5.38	6.16	7.72	4.57	2.88	4.82	4.52	6.28	5.51
49	5.55	6.14	5.99	3.76	3.45	5.06	4.9	6.14	5.99
50	5	5.8	5.94	3.07	4.17	5.29	5.16	6.73	5.19
51		6.11	5.91	2.51	3.74	4.54	5.19	5.98	6.1
52	7.43	4.91	5.35	2.51	4.46	4.54	4.84	6.19	5.14
53	6.13	5.6	5.48	2.5	4.06	4.56	5.17	5.97	5.84
54	5.5	4.86	9.78	2.94	3.24	4.98	4.73	6.63	5.88
55	4.9	4.81	18.02	2.88	3.99	4.9	5.01	6.6	5.94
56	4.76	5.4	10	2.64	3.94	4.69	4.66	6.71	5.83
57	5.32	5.43	9.09	2.66	4.03	5.69	4.66	6.09	5.17
58	5.5	4.78	12.36	2.65	3.45	5.07	5.19	6.54	5.97
59	5.41	5.36	12.24	2.63	4.54	4.98	4.66	6.24	5.31
60	5.2	5.41	10	2.59	4.47	4.25	5.19	6.62	6.32
61	5.86	5.38	10.29	2.47	4.55	4.35	4.72	6.41	6.13
62	5.84	5.4	9.36	2.39	3.88	4.24		6.82	5.99
63	6	5.5	8.55	2.92	3.92	4.61	4.83	6.73	6.26
64	6.01	5.72	8.76	2.22	3.99	4.62	5.31	6.21	6.1
65	6.02	5.38	10.24	2.62	3.81	4.58	5.01	6.02	6.19
66	5.59	4.84	9.12	2.6	4.18	4.63	5.46	6.52	5.97
67	5.54	4.86	8.56	2.66	4	4.66	4.82	6.48	5.81
68	5.68	4.84	10.35	2.67	4.53	4.58	5.18	5.73	6.32
69	6.23	4.93	9.56	2.61	4.22	4.65	5.64	5.69	6.02
70	5.71	5.37	9.93	2.36	4.33	4.05	5.22	5.7	6.3
71	6.41	5	8.74	3.01	3.99	4.5	5.68	6.29	6.23
72	5.79	5.59	9.1	2.77	4.66	4.73	5.06	5.54	5.81
73	5.79	5.7	10.03	2.7	4.63	4.44	5.07	5.91	5.62
74	6.18	5.63	10.67	2.65	4.31	4.5	4.65	6.44	5.58
75	5.86	5.03	10.2	2.38	3.4	4.17	4.78	6.16	5.73
76	5.9	5.09	9.13	5.78	3.6	4.2	4.77	5.99	5.66
77	5.25	5.49	9.13	3.6	4.59	3.97	4.81	6.16	5.55
78	5.81	4.95	9.86	3.2	3.64	3.85	4.83	6.15	6.3
79	6.06	5.47	8.67	3.14	4.2	4.31	4.97	5.59	5.84
80	5.8	5.4	7.91	6.12	4.43	4.36	4.92	5.98	4.96
81	5.36	5.39	8.12	4.91	3.59	4.02	4.39	5.53	5.48
82	5.79	5.52	7.22	5.41	4.57	4.16	4.91	5.63	6.38
83	5.44	5.22	6.11	5.28	4.51	4.17	4.92	6.3	6.41
84	5.86	5.23	6.23	4.97	4.29	4.27	4.92	6.26	6.09

85	6.09	5.84	6.81	4.34	3.96	4.23	4.87	5.78	5.88
86	5.61	5.81	5.51	4.24	4.56	3.57	4.86	6.27	5.48
87	6.07	5.68	5.62	4.6	3.77	4.61	4.9	6.18	5.4
88	5.29	5.73		7.19	4.79	4.54	4.5	6.17	5.89
89	5.41	5.62		6.52	4.62	4.53	4.88	5.61	5.9
90	5.6	5.64			3.92	4.61	4.92	5.46	6.14
91	5.52	6			4.81	4.35	5.51	5.56	5.87
92	5.58	6.26			5.03	4.53	4.46	5.41	5.7
93	4.85	6.9			4.85	4.62	4.42	5.3	5.82
94	4.76	7.03			5.21	4.28	4.45	5.31	6.08
95	4.77	6.69			4.1	4.51	4.36	5.32	5.9
96	4.76	6.72			4.83	4.14	4.41	5.26	5.8
97	5.23	6.74			5.2	4.24	4.86	5.18	5.25
98	4.99	7.71			5.54	4.5	4.69	5.82	5.93
99	5.65	7.18			5.13	4.52	4.88	5.28	5.5
100	7.71	7.09			4.76	4.31	4.54	5.93	7.12
101	7.14				4.95	4.43	5.13	5.34	5.64
102	6.49				5.55	4.42	4.68	5.99	5.52
103					5.91	4.77	4.73	5.38	5.13
104					5.17	4.77	5	6.22	5.39
105					5.24	4.92	4.92	6.1	5.45
106					5.21	4.65	4.57	5.97	5.23
107					5.59	4.53	4.53	6.06	5.26
108					6.72	4.54	4.99	5.44	5.24
109					6.85	4.28	4.55	5.25	5.07
110					8.16	4.61	4.49	5.33	5.62
111					8.67	4.9	4.52	5.93	5.34
112					8.15	4.21	4.71	5.91	5.13
113					7.45	4.74	4.72	5.42	5.65
114					8.14	4.44	5.14	5.37	5.51
115					8.61		5.17	5.41	5.17
116					8.07	4.01	5.07	5.76	5.82
117					10.1	4.57	4.99	5.7	5.71
118					10.7	4.47	5.06	5.09	5.41
119					13.68	4.9	5.12	5.55	5.56
120					13.02	4.61	5.06	6.05	5.3
121						4.26	4.96	5.29	5.53
122						5.54	4.5	5.48	5.48
123						5.24	4.47	4.99	5.79
124						5.03	4.85	5.04	5.85
125						5.23	4.54	5.18	5.3
126						5.4	4.45	5.18	5.55
127						5.49	4.46	5.84	5.75
128						5.46	4.99	6.48	5.84

129		4.41	5.92	5.54
130	5.76	4.42	5.46	5.24
131	5.51	4.39	5.96	5.39
132	5.62	4.48	5.89	5.83
133	5.3	4.46	5.76	6.22
134	5.43	5.05	4.89	5.87
135	6.27	4.94	5.26	5.43
136	5.56	5.09	5.51	5.5
137	5.76	4.75	5.36	5.75
138	6.26	4.39	5.69	5.75
139	6.68	4.33	5.13	5.19
140	5.78	4.82	5.43	5.19
141	5.88	4.33	5.05	4.99
142	6.79	4.82	4.9	5.69
143	6.02	4.3	5.81	5.46
144	6.24	4.26	4.9	5.38
145	6.37	4.23	4.89	4.75
146	6.06	4.76	4.76	4.62
147	5.86	4.85	4.85	5.09
148	5.45	4.73	5.33	5.51
149	6.81	4.35	5.81	4.86
150	6.51	4.72	5.23	5.01
151	6.5	4.72	5.49	5.12
152	6.21	4.65	4.92	5.39
153	5.25	4.28	4.88	5.27
154	5.47	4.7	5.18	5.63
155	5.56	4.7	5.41	5.06
156	6.21	4.17	4.87	5.24
157		4.18	5.57	4.8
158		4.63	4.51	5.21
159			4.6	5.39
160		4.33	4.46	4.83
161		4.84	4.61	5.77
162		4.11	4.65	5.12
163		4.15	4.69	5.35
164		4.42	5.59	4.08
165		4.29	4.65	4.76
166		4.37	4.63	4.6
167		4.25	4.57	5.03
168		4.34	5.06	4.24
169		4.83		
170		4.3		
171		4.56		
172		4.43		

173	4.33
174	4.26
175	4.39
176	4.27
177	4.72
178	4.43
179	4.35
180	4.32
181	4.34
182	4.65
183	4.52

Figure 2 **Total Flow (ml / 100g / min)**

Time (Hours)	A	B	C	D	1	2	3	4
0.5	99.36489607	98.7603306	122.168675	81.84210526	123.964497	64.29423459	72.4248927	81.63507109
1	102.482679	90.857438	133.815261	90.84210526	101.8737673	86.16302187	82.13519313	90.69905213
2	108.3718245	105.320248	148.353414	101.5263158	136.3905325	80.55666004	103.7553648	106.457346
3	111.778291	101.342975	147.791165	100.5263158	121.6962525	87.67395626	106.5450644	121.3862559
4	117.147806	96.3842975	133.092369	95.73684211	122.6824458	82.18687873	115.6652361	126.4810427
5	114.4341801	96.3842975	145.220884	95.36842105	139.0532544	75.66600398	107.4570815	118.3649289
6	111.0854503	97.8822314	159.598394	94.78947368	142.0118343	77.37574553	108.9055794	115.4620853
7	112.0092379	113.326446	160.722892	99.42105263	129.1913215	77.05765408	109.0128755	109.2417062
8	108.8337182	115.082645	159.35743	97.36842105	152.1696252	76.81908549	109.4957082	107.5236967
9	107.6789838	114.307851	157.590361	95.73684211	142.7021696	76.38170974	110.0858369	108.4123223
10	111.8937644	102.272727	153.333333	96.47368421	138.5601578	74.27435388	109.8175966	111.9668246
11	103.926097	100.878099	164.337349	97.68421053	142.9980276	73.32007952	109.3347639	111.2559242
12	110.1616628	100.309917	165.542169	94	130.7692308	72.80318091	367.7038627	106.5165877
13	108.6605081	105.061983	176.706827	93.52631579	127.7120316	72.12723658	113.6802575	115.3436019
14	110.0461894	106.508264	169.156627	94.57894737	128.5996055	75.14910537	113.4120172	116.4691943
15	107.7944573	106.198347	177.991968	95.63157895	120.8086785	78.01192843	112.0708155	113.6848341
16	110.2193995	107.024793	181.526104	97.68421053	99.11242604	86.99801193	114.6995708	115.4028436
17	109.7575058	104.080579	183.534137	97.84210526	163.2149901	82.26640159	26.80364807	114.0402844
18	102.3672055	102.066116	170.281124	98.63157895	128.5009862	78.96620278	105.6330472	113.9218009
19	103.1755196	98.9152893	151.807229	98.15789474	125.9368836	91.05367793	101.6094421	114.6327014
20	103.1177829	97.4173554	173.895582	97.52631579	132.642998	87.83300199	101.9849785	113.9218009
21	104.330254	99.9483471	174.297189	97.42105263	94.87179487	91.57057654	103.0042918	111.6113744
22	105.2540416	102.892562	146.26506	97.26315789	117.8500986	100.4373757	103.1652361	110.07109
23	104.3879908	102.117769	145.140562	102.4210526	123.2741617	99.72166998	105.2575107	108.471564
24	102.7713626	101.60124	174.939759	101.3157895	117.061144		106.223176	116.2322275
25	103.0600462	99.0702479	178.232932	99.63157895	146.5483235	99.12524851	93.88412017	112.7962085
26	97.92147806	101.136364	158.634538	96.42105263	152.5641026	94.83101392	114.2167382	118.7203791
27	109.6997691	110.330579	169.477912	96.68421053	141.2228797	99.04572565	104.5600858	111.4336493
28	111.1431871	107.747934	169.076305	101.2105263	141.4201183	98.44930417	105.0429185	117.1800948
29	111.3741339	79.1322314	170.120482	98.10526316	145.0690335	96.89860835	112.7145923	112.0260664
30	116.0508083	117.716942	162.971888	96.52631579	142.3076923	93.95626243	107.1888412	114.3957346
31	113.9722864	113.378099	157.751004	98.68421053	133.4319527	93.99602386	105.7939914	115.3436019
32	117.6674365	125.154959	168.433735	98.31578947	128.3037475	97.29622266	105.9549356	117.0616114
33	113.1639723	105.681818	166.907631	99.31578947	133.82643	98.52882704	107.8862661	107.9976303
34	109.5265589	95.1446281	134.056225	99.10526316	142.4063116	94.99005964	112.4463519	109.6563981
35	109.4110855	108.93595	153.895582	98.63157895	130.8678501	101.5506958	107.2961373	108.1161137
36	113.3949192	93.9049587	159.759036	97.52631579	128.8954635	101.3121272	114.9141631	109.2417062
37	114.5496536	91.7355372	128.594378	97.31578947	123.4714004	79.20477137	115.3433476	110.2488152
38	113.5681293	88.6363636	128.192771	97.15789474	121.1045365	96.02385686	115.0214592	110.6042654
39	110.334873	93.5950413	127.871486	96.47368421	117.061144	100.9145129	106.276824	110.7227488
40	108.4295612	111.828512	112.369478	99.57894737	117.9487179	95.42743539	106.223176	103.7914692

41	102.6558891	94.6797521	124.016064	99.73684211	116.765286	104.4135189	108.583691	108.2345972
42	100.5773672	91.6838843	68.5943775	97.68421053	116.9625247	100.2385686	109.2274678	111.492891
43	100.404157	96.3326446	95.1004016	97.47368421	110.7495069		109.3347639	106.1611374
44	102.9445727	89.1012397	108.433735	98.05263158	129.6844181	98.33001988	107.8862661	112.7369668
45	101.7898383	90.1859504	104.819277	100	138.1656805	97.69383698	102.4141631	110.7227488
46	104.5034642	93.0785124	139.678715	100.3684211	135.3057199	95.90457256	99.35622318	111.492891
47	102.4249423	108.57438	138.955823	99.31578947	135.0098619	91.17296223	111.1051502	113.507109
48	99.13394919	93.6466942	98.7951807	102	137.0808679	84.33399602	113.4120172	112.6777251
49	98.61431871	106.869835	135.662651	99.26315789	127.2189349	76.97813121	110.139485	123.1042654
50		108.006198	133.253012	124.9473684	134.2209073	73.35984095	109.1201717	106.9905213
51	93.36027714	109.865702	135.662651	130.2631579	116.6666667	79.72166998	108.6909871	115.521327
52	108.7759815	118.130165	137.028112	131.2105263	147.4358974	77.69383698	111.5879828	112.3222749
53	111.6050808	118.233471	134.056225	130.6842105	131.4595661	92.16699801	110.4077253	120.2606635
54	117.2632794	114.10124	95.2610442	130.8947368	127.6134122	80.31809145	109.0128755	113.0331754
55	112.7020785	122.055785	73.0120482	132.5789474	125.739645	80.03976143	113.4656652	113.3886256
56	111.0277136	119.214876	124.497992	136.8421053	126.5285996	90.89463221	112.2317597	113.4478673
57	111.3163972	122.985537	127.309237	138.3157895	126.2327416	80	110.7832618	110.2488152
58	106.7551963	120.196281	96.7871486	138.2631579	126.3313609	79.60238569	113.3583691	113.9218009
59	110.8545035	121.642562	106.827309	138.2631579	135.9960552	80.31809145	112.9291845	109.4194313
60	109.6420323	121.022727	121.686747	138.5263158	137.8698225	80.15904573	106.276824	111.9668246
61	107.6212471	119.11157	120.401606	140.6315789	136.0946746	94.27435388	111.8562232	105.2132701
62	105.9468822	118.646694	109.799197	146.4210526	135.7001972	103.8966203		114.6327014
63	102.8290993	116.42562	129.799197	113.0526316	135.7001972	89.66202783	107.0815451	113.507109
64	102.3094688	122.830579	117.670683	118.6842105	135.0098619	101.8290258	106.9742489	113.5663507
65	100.5196305	121.797521	104.016064	119.4210526	136.4891519	101.9085487	108.3690987	117.0616114
66	101.6166282	119.421488	109.156627	120.5263158	109.9605523	101.9880716	104.5064378	118.3056872
67	98.78752887	117.303719	125.542169	118.4736842	118.5404339	93.08151093	108.9055794	120.8530806
68	98.90300231	119.886364	105.702811	122.8421053	138.2642998	96.69980119	102.8969957	123.5189573
69	94.45727483	120.661157	115.823293	121.1052632	118.3431953	96.77932406	112.5536481	121.6824645
70	94.91916859	116.683884	108.192771	121.4736842	115.0887574	79.68190855	115.3433476	108.6492891
71	94.80369515	117.871901	108.835341	93.26315789	114.3984221	92.68389662	112.9291845	116.528436
72	104.330254	114.152893	114.859438	98.10526316	142.4063116	100.4771372	111.2660944	115.4620853
73	104.2147806	116.012397	111.88755	115.6315789	136.0946746	94.91053678	111.1587983	112.4407583
74	100.1154734	116.632231	106.184739	118.6842105	133.530572	97.89264414	115.9871245	119.0758294
75	90.70438799	113.171488	94.8594378	117.1578947	127.4161736	87.43538767	118.2403433	123.4597156
76	90.41570439	115.289256	109.477912	83.89473684	118.4418146	92.32604374	107.5107296	115.936019
77	103.2332564	116.06405	107.630522	94.36842105	123.5700197	99.88071571	106.4377682	106.5165877
78	94.57274827	119.059917	111.164659	105.0526316	123.2741617	98.36978131	117.0600858	108.8270142
79	106.1200924	120.402893	124.97992	106.2631579	121.5976331	99.04572565	118.6158798	108.8862559
80	89.20323326	113.997934	137.429719	89.26315789	120.1183432	94.87077535	114.3240343	114.5734597
81	102.8290993	115.392562	113.654618	85.10526316	117.65286	97.61431412	116.5772532	107.464455
82	97.17090069	77.1694215	111.164659	96.68421053	125.739645	92.12723658	116.1480687	112.2037915
83	101.5588915	112.396694	120.240964	104.0526316	146.6469428	101.5109344	114.5922747	108.5308057
84	96.93995381	110.433884	105.220884	105.1578947	125.739645	98.80715706	114.5922747	110.3672986

85	99.82678984	109.090909	108.594378	107.4736842	134.3195266	98.96620278	117.167382	111.1374408
86	98.72979215	109.81405	126.104418	121.2631579	134.7140039	95.66600398	117.1137339	118.1279621
87	107.5057737	105.733471	132.208835	106.5789474	138.0670611	99.48310139	114.1094421	112.7369668
88	107.3903002	107.128099		76.57894737	142.9980276	90.61630219	115.4506438	115.8175355
89	107.3903002	108.93595		99.57894737	138.9546351	99.52286282	114.9141631	114.5734597
90	105.369515	104.752066			134.8126233	100.4373757	113.1437768	115.9952607
91	103.1755196	102.117769			125.5424063	100.4373757	97.74678112	118.1279621
92	101.8475751	116.219008			134.122288	97.41550696	107.6180258	113.9810427
93	101.7321016	111.208678			132.4457594	93.87673956	113.1437768	115.1658768
94	98.44110855	110.950413			134.122288	98.48906561	112.0171674	113.9218009
95	96.24711316	111.466942			135.1084813	98.17097416	113.7339056	113.9810427
96	86.48960739	110.072314			122.1893491	99.60238569	114.6995708	115.8767773
97	84.18013857	108.264463			120.5128205	99.16500994	119.0987124	125.2962085
98	76.61662818	106.508264			137.3767258	92.76341948	121.4592275	115.2251185
99	60.1039261	102.840909			132.8402367	97.89264414	114.5922747	114.8696682
100	75.63510393	104.597107			135.5029586	100.3180915	116.1480687	115.4028436
101	77.2517321				132.34714	96.38170974	110.944206	120.3199052
102					134.7140039	97.73359841	110.6759657	116.5876777
103					117.357002	90.77534791	112.3390558	113.3293839
104					139.4477318	91.96819085	113.7339056	113.5663507
105					143.0966469	100.1192843	112.7145923	108.2345972
106					118.6390533	102.7833002	112.8218884	113.2109005
107					130.1775148	97.73359841	114.3240343	118.1279621
108					117.1597633	102.0675944	111.8025751	116.6469194
109					139.4477318	99.04572565	114.6995708	108.2938389
110					122.3865878	104.2942346	116.2553648	109.8933649
111					136.0946746	99.32405567	110.5150215	114.8696682
112					133.234714	106.1630219	114.2703863	116.4099526
113					138.7573964	101.2723658	107.0815451	116.8246445
114					127.0216963	103.8170974	108.3690987	119.1350711
115					146.0552268		105.5793991	118.3056872
116					141.2228797	103.0616302	106.223176	121.0308057
117					126.7258383	106.4811133	111.8025751	121.8601896
118					115.9763314	105.2485089	109.6030043	116.4099526
119					115.6804734	99.00596421	110.139485	122.2156398
120					126.3313609	105.6063618	116.0944206	107.5829384
121						95.50695825	113.8412017	123.8744076
122						93.71769384	116.0944206	125.8886256
123						97.05765408	115.4506438	126.8957346
124						98.33001988	117.972103	122.5118483
125						97.3359841	116.3090129	117.3578199
126						92.36580517	115.5042918	121.4454976
127						91.29224652	115.7188841	117.6540284
128						91.7693837	106.4914163	105.9241706

129		114.0021459	110.1895735
130	97.09741551	114.860515	117.7132701
131	98.56858847	116.3090129	120.2606635
132	98.48906561	115.8261803	122.7488152
133	101.2723658	109.388412	123.0450237
134	98.72763419	113.1437768	115.3436019
135	99.68190855	115.7725322	110.4265403
136	101.1133201	108.9592275	106.3388626
137	97.61431412	110.3004292	111.2559242
138	96.30218688	115.1287554	123.4004739
139	97.93240557	116.8454936	111.7298578
140	93.43936382	119.3133047	111.1966825
141	105.5666004	115.8261803	109.3009479
142	95.70576541	117.2746781	114.5734597
143	98.21073559	119.7961373	119.0165877
144	98.56858847	118.8841202	118.7203791
145	98.21073559	120.332618	111.3744076
146	98.36978131	120.9763948	112.1445498
147	100.7952286	122.0493562	117.8317536
148	100.9542744	113.251073	108.056872
149	93.55864811	120.7618026	109.9526066
150	96.26242545	122.1030043	119.0165877
151	96.5805169	122.8004292	125.1184834
152	98.21073559	123.3369099	119.3720379
153	102.6640159	122.639485	119.7867299
154	109.0258449	123.2832618	117.535545
155	107.4353877	121.9957082	117.6540284
156	99.88071571	124.6244635	113.3293839
157		124.5708155	116.1729858
158		124.7854077	116.7061611
159			112.9146919
160		107.6180258	103.5545024
161		108.8519313	109.7156398
162		121.2446352	110.3080569
163		122.2103004	113.7440758
164		115.7725322	113.0924171
165		117.0064378	122.0379147
166		117.167382	121.2677725
167		117.3283262	120.1421801
168		119.7961373	122.0971564
169		120.1716738	
170		119.0987124	
171		117.2746781	
172		121.083691	

173	119.7424893
174	119.8497854
175	118.6695279
176	120.1716738
177	120.5472103
178	121.083691
179	119.7424893
180	119.0450644
181	119.2596567
182	116.8454936
183	116.3090129

5
14.39351593
41.47568474
57.29457798
65.23197317
69.61989939
71.85578535
76.02012297
79.56959195
80.82727781
85.49468977
84.68418111
88.68082728
89.40749022
90.58133035
87.59083287
83.76187814
83.70598099
86.72442705
85.91391839
84.9077697
80.38010061
80.35215204
86.08160984
86.64058133
86.55673561
87.19955282
87.0039128
85.85802124
85.43879262
85.43879262
84.68418111
86.75237563
87.98211291
83.90162102
82.58803801
84.34879821
84.9077697
81.66573505
85.38289547
83.73392957
83.39854667

80.77138066

85.77417552

86.16545556

88.93236445

86.50083846

87.92621576

86.52878703

83.95751817

81.94522079

83.42649525

85.77417552

86.08160984

85.15930688

80.60368921

85.63443264

83.09111235

80.54779206

87.59083287

80.29625489

78.73113471

79.2901062

80.91112353

83.39854667

85.04751258

79.09446618

83.65008385

78.28395752

78.92677473

81.86137507

84.06931247

85.88596982

91.50363331

83.11906093

84.62828396

86.08160984

87.28339855

74.70653997

79.26215763

88.68082728

78.53549469

71.57629961

80.04471772

79.31805478

81.80547792
86.47288988
83.92956959
79.79318055
82.36444941
78.64728899
85.96981554
84.9077697
84.79597541
79.8490777
78.87087759
82.56008944
82.92342091
83.1470095
80.68753494
75.85243153
85.21520402
89.63107881
90.91671325
87.50698714
89.63107881
84.32084964
81.21855785
89.57518167
91.36389044
84.74007826
76.91447736
86.38904416
86.41699273
88.28954723
84.20905534
77.05422023
81.21855785
83.2588038
83.51034097
87.64673002
79.93292342
78.45164897
82.28060369
81.4980436
79.8490777
83.4823924
78.87087759
78.84292901

77.8647289
86.80827278
82.14086082
78.56344326
84.12520961
86.75237563
86.08160984
81.66573505
82.75572946
84.54443823
90.7490218
89.35159307
90.30184461
82.89547233
82.72778088
86.41699273
93.57182784
86.08160984
79.59754053
76.1039687
82.33650084
84.15315819
78.25600894
84.46059251
83.84572387
81.7216322
85.71827837
79.48574623
82.44829514
76.49524874
83.31470095
89.2118502
79.93292342
86.7803242
89.96646171
88.37339296
88.48518726
85.04751258
80.82727781
94.29849078

Figure 3 **Methaemaglobin (%)**

Time (Hours)	A	B	C	D	1	2	3	4
0	0.7	0.6	0.5	0.5	0.5	0.5	1.1	0.5
1	0.6	0.7	0.6	0.5	0.5	0.7	0.7	0.6
2	0.5	0.6	0.6	0.5	0.7	0.7	0.7	0.7
3	0.6	0.6	0.8	0.5	0.7	0.7	0.7	0.7
4	0.5	0.6	0.7	0.6	0.7	0.8	0.7	0.8
5	0.5	0.6	0.7	0.6	0.6	0.8	0.6	0.8
6	0.6	0.6	0.6	0.6	0.8	0.8	0.6	0.8
7	0.6	0.7	1.4	0.5	0.7	0.7	0.5	0.7
8	0.6	0.5	1.2	0.6	0.6	0.7	0.6	0.7
9	0.7	0.6	1.2	0.5	0.7	0.6	0.7	0.6
10	0.6	0.7	1.1	0.6	0.8	0.6	0.5	0.7
11		0.7	1	0.5	0.6	0.6	0.6	0.6
12	0.5	0.6	1	0.6	0.8	0.7	0.5	0.5
13	0.5	0.7	1.1	0.6	0.8	0.7	0.5	0.6
14	0.5		1	0.5	0.8	0.5	0.5	0.5
15		0.2	1.2	0.6	0.9	0.6	0.6	0.6
16	0.5	0.6	1.1	0.5	0.7	0.6	0.5	0.6
17	0.7	0.6	1.1	0.6	0.6	0.6	0.5	0.6
18	0.6	0.5	1.2	0.5	0.6	0.5	0.6	0.6
19	0.5	0.6	1.2	0.6	0.8	0.7	0.5	0.5
20	0.5	0.6	1.3	0.6	0.7	0.7	0.6	0.5
21	0.4	0.6	1.3	0.6	0.7	0.7	0.5	0.4
22	0.6	0.7	1.4	0.5	0.7	0.7	0.4	0.5
23	0.8	0.6	1.4	0.4	0.7	0.7	0.4	0.4
24	0.9	0.8	1.4	0.5	0.6	0.8	0.5	0.6
25	1	0.6	1.4	0.4	0.7	0.6	0.5	0.5
26	1.1	0.7	1.5	0.5	0.8	0.6	0.5	0.5
27	1.3	0.5	1.5	0.5	0.7	0.7	0.6	0.6
28	1.3	0.8	1.6	0.5	0.8	0.7	0.5	0.6
29	1.3	0.7	1.7	0.6	0.7	0.7	0.5	0.6
30	1.3	0.9	1.9	0.5	0.7	0.9	0.6	0.5
31	1.3	0.8	2	0.6	0.7	0.7	0.6	0.5
32	1.7	0.8	2.1	0.5	0.7	0.7	0.5	0.6
33	1.6	0.7	2.4	0.4	0.7	0.8	0.5	0.5
34	2.2	0.9	2.6	0.6	0.9	0.7	0.5	0.4
35	2.2	0.8	2.9	0.5	0.9	0.7	0.6	0.6
36	2.3	0.7	3.1	0.4	0.7	0.8	0.6	0.6
37	2.5	0.8	3.3	0.6	0.8	0.7	0.5	0.5
38	2.6	0.9	3.7	0.4	0.9	0.6	0.5	0.5
39	2.8	0.7	4.1	0.5	0.7	0.7	0.6	0.6
40	2.4	0.9	4.5	0.4	1	0.8	0.6	0.6

41	3	0.9	4.9	0.6	0.9	0.6	0.6	0.6
42	4.3	0.9	5.6	0.6	0.9	0.8	0.5	0.6
43	4	0.9	6.6	0.6	0.9	0.8	0.6	0.5
44	5.2	0.9	7.1	0.5	0.8	0.8	0.5	0.5
45	5.9	1	6.8	0.5	0.9	0.7	0.5	0.5
46	5.4	0.9	7.1	0.5	0.9	0.7	0.6	0.5
47	9.6	1	7.1	0.5	0.9	0.7	0.6	0.5
48	7.8	1	7.5	0.5	0.9	1	0.5	0.6
49	8.4	1.1	7.8	0.4	1	0.8	0.6	0.5
50	11.5	1	8.6	0.4	0.9	0.7	0.5	0.5
51	14.3	1.1	9.1	0.4	1	0.6	0.6	0.4
52	4	1.2	9.4	0.3	1.1	0.6	0.5	0.6
53	4.3	1.5	9.7	0	0.9	0.8	0.6	0.5
54	4.6	1.2	10.2	0.4	1	0.7	0.6	0.6
55	4.6	1.2	10.1	0.4	1	0.8	0.4	0.5
56	4.8	1.2	9.8	0.4	0.9	0.8	0.6	0.5
57	4.9	1.3	9.9	0.5	1	0.8	0.5	0.5
58	5	1.3	10.4	0.5	1	0.9	0.6	0.4
59	5.2	1.3	10.9	0.6	0.9	0.8	0.5	0.5
60	5.4	1.3	9.4	0.5	1	0.7	0.6	0.6
61	5.7	1.5	9.6	0.5	1.1	0.8	0.5	0.6
62	5.7	1.3	5.9	0.5	1	0.9	0.5	0.5
63	5.8	1.5	6.9	0.5	1	0.7	0.5	0.5
64	6.1	1.5	9.2	0.4	1	0.8	0.4	0.6
65	5.5	1.7	12.1	0.3	1	0.8	0.5	0.5
66	5.6	1.7	15.6	0.5	1	0.8	0.5	0.6
67	6.7	1.7	19.8	0.4	1	0.9	0.6	0.6
68	6.8	1.7	23.2	0.6	0.9	0.8	0.5	0.5
69	7.1	1.8	27.6	0.3	0.9	0.8	0.6	0.6
70	6.9	2	31.9	0.4	1	0.9	0.6	0.5
71	8.6	2	35.3	0.4	1.1	0.7	0.6	0.6
72	8.6	2.1	37.3	0.5	0.9	0.8	0.5	0.5
73	8.9	2.4	41.1	0.5	0.9	0.7	0.5	0.5
74	9.3	2.4	42.8	0.5	1	0.6	0.5	0.5
75	6	2.5	45	0.6	0.9	0.9	0.5	0.5
76	6.6	2.8	42.8	0.6	1	0.7	0.8	0.5
77	6.9	2.4	39.5	0.5	0.9	0.7	1.6	0.5
78	7.3	2.3		0.5	1	0.8	0.6	0.5
79	7.8	2.2	34.6	0.7	1	0.8	0.5	0.6
80	8.1	2.3	33.1	0.8	0.8	0.8	0.5	0.5
81	8.6	2.3	32.6	0.9	1	0.9	0.5	0.5
82	8.8	2.4	32.4	1	1	0.7	0.6	0.5
83	8.9	2.6	33.2	1.2	0.8	0.8	0.5	0.4
84	10	2.6	32.9	1.4	0.9	0.8	0.5	0.6

85	10.5	2.8	32.6	2	0.8	0.7	0.5	0.5
86	11	2.9	32.4	2.7	1	0.8	0.5	0.5
87	11.5	3.2	31.4	4.6	1	0.7	0.5	0.6
88	10.7	3.4		9.3	1	0.7	0.5	0.6
89	10.9	3.5		16.5	0.9	0.7	0.5	0.5
90	12.6	3.6		28	1	0.7	0.4	0.5
91	14.7	3.8			0.9	0.7	0.4	0.6
92	15.8	4.2			1	0.6	0.5	0.5
93	16.1	4.3			0.9	0.6	0.6	0.5
94	17.9	4.6			0.9	0.7	0.6	0.5
95	19.2	4.7			0.9	0.7	0.5	0.6
96	20.8	5.1			0.9	0.6	0.6	0.6
97	25.3	5.4			1	0.7	0.5	0.5
98	28.5	5.5			1	0.5	0.5	0.4
99	33.5	5.8			0.9	0.5	0.5	0.5
100	36.7	6.1			1.1	0.8	0.6	0.4
101	17.8				1	0.6	0.6	0.5
102	18				1	0.6	0.7	0.6
103					1	0.7	0.6	0.5
104					0.9	0.7	0.7	0.5
105					1	0.7	0.6	0.6
106					1	0.7	0.7	0.6
107					1	0.7	0.5	0.5
108					0.9	0.8	0.6	0.5
109					0.9	0.8	0.6	0.6
110					1.3	0.7	0.5	0.5
111					1	0.7	0.5	0.6
112					0.9	0.8	0.6	0.5
113					0.8	0.7	0.6	0.6
114					0.8	0.7	0.4	0.7
115					0.8	0.7	0.6	0.7
116					0.8	0.7	0.5	0.4
117					0.7	0.7	0.5	0.6
118					0.6	0.6	0.6	0.6
119					0.7	0.7	0.5	0.5
120					0.7	0.6	0.6	0.5
121					0.6	0.7	0.6	0.5
122							0.5	0.5
123							0.5	0.6
124							0.5	0.4
125							0.6	0.5
126							0.5	0.4
127							0.5	0.4
128							0.5	0.5

129		0.6	0.5
130		0.5	0.3
131		0.5	0.4
132		0.6	0.4
133		0.5	0.4
134		0.6	0.4
135		0.6	0.5
136		0.5	0.4
137		0.5	0.5
138		0.6	0.5
139		0.6	0.6
140	0.7	0.6	0.5
141		0.6	0.4
142	0.7	0.6	0.5
143	0.7	0.5	0.5
144	0.7	0.5	0.6
145	0.7	0.5	0.6
146	0.7	0.4	0.4
147	0.7	0.6	0.5
148	0.8	0.6	0.4
149	0.7	0.6	0.5
150	0.8	0.5	0.5
151		0.6	0.5
152	0.8	0.6	0.7
153		0.6	0.7
154	0.8	0.5	0.5
155		0.5	0.4
156	0.9	0.5	0.5
157	0.8	0.5	0.5
158	0.8	0.5	0.6
159		0.5	0.5
160	0.9	0.4	0.6
161	0.9	0.5	0.5
162	0.9	0.6	0.6
163	0.9	0.5	0.6
164	1	0.6	0.6
165	1.1	0.7	0.4
166	1.1	0.5	0.5
167	1.2	0.6	0.4
168	1.3	0.5	0.5
169	1.5	0.6	
170	1.9	0.6	
171	2	0.5	
172	2.2	0.6	

173	2.4	0.6
174	2.8	0.6
175	3.2	0.6
176	3.4	0.6
177	4	0.6
178	4.6	0.6
179	4.2	0.6
180		0.6
181		0.7
182		0.7
183		0.7
184		0.6

5
0.5
0.8
0.9
0.9
0.8
1
0.9
1
0.9
0.9
0.7
0.7
0.6
0.7
0.5
0.7
0.6
0.6
0.6
0.7
0.8
0.7
0.7
0.8
0.7
0.8
0.8
0.8
0.8
0.9
1
0.9
1
1
0.9
0.9
0.9
1
0.9
1
1.1

1

1.1

1

1

1

1

1.1

1.2

1.2

1.1

1.2

1.2

1.1

1.1

1.2

1

1.1

1.1

1.2

1.1

1.1

1.1

1.1

1.1

1.1

1.1

1

1.2

1.1

1.1

1.1

1.1

1

1

1

1.1

1

1

1.1

1

1

1

1.1

0.9

0.9
1
0.9
0.9
0.9
0.9
1
0.9
1
0.8
0.9
1
0.9
0.9
0.8
0.9
0.9
0.9
0.9
1
0.9
1
1
1.1
1
1
1
1
1.1
1

1.1
1
1
1
1.1
1
0.9
1.1
1
1
1
1

1
1
1
1
1.2
1.1
1
1
1
1
1
1
0.9
1
1
0.8
1
1
0.9
1
1
0.8
1
0.9
1
1
0.9
1
0.9
1
1
1
1
1
1
1
1
1
0.9
1

Figure 4 Lactate (mmol/L)

Time (Hours)	A	B	C	D	1	2	3	4	5
0	20	9.86	7.93	7.55	6.62	9.67	20	8.81	9.71
0.25					6.77	13.9	12.59		13.59
0.33						11.29		8.44	
0.5					5.38		9.45	6.78	14.12
1	6.81	6.32	2.8	5.02	4.06	13.48	5.14	6.32	
1.5						10.67	2.85		13.49
2	1.78	0.32	1.31	0.89	2.66	8.9	2.44	2.01	13.87
2.5								2.1	
3	1.5	0	0.61	1.36	1.97	3.77	1.58	2.28	12.41
3.5								2.54	
4	1.76	1.07	1.37	2.09	1.77	3.75	1.35	2.47	9.71
5	1.79	1.24	1.9	1.35	1.95	3.98	1.12	1.95	7.04
6	1.67	1.51	2.06	0.91	1.43	2.92	0.83	1.94	4.63
7	1.34	1.38	2.14	0.93	0.51	1.82	0.65	1.61	3.11
7.5									2.55
8	1.21	1.46	1.81	0.75	1.29	1.11	0.6	1.06	2.33
9	1.06	1.47	1.95	0.84	2.93	0.83	0.68	0.94	1.7
10	0.98	1.41	1.57	0.8	0.9	0.75	0.38	0.72	1.49
11		1.15	1.52	1	1.44	1.31	0.96	1.23	1.85
12	1.18	1.28	1.34	0.61	1.99	1.73	1.52	0.95	1.86
13	1.04	1.47	1.67	0.78	0	1.88	1.82	1.96	1.71
14	1.22	0.83	1.3	0.82	0	1.7	2.12	1.99	1.71
15		0.81	0.98	0.82	0.35	1.59	2.1	1.99	1.84
16	0.81	1.28	1.11	1.21	1.16	1.67	1.71	2	1.94
17	0.79	1.58	0.9	1.01	1.26	1.75	1.16	2.27	1.85
18	0.8	1.28	0.93	1.51	3.5	1.78	0.97	1.89	2.09
19	0.57	2.49	1.04	2.06	2.81	1.68	1.13	1.54	2.12
20	0.63	2.81	0.95	2.25	2.66	1.76	1.54	1.52	2.98
21	0.66	3.28	0.96	2.23	0.69	1.86	1.97	1.61	1.91
22	0.64	2.3	0.94	2.17	1.37	1.94	2.26	1.06	1.18
23	0.68	2.78	1.17	1.3	1.2	1.85	2.51	2.9	1.37
24	0.71	2.61	0.62	1.64	1.14	2.24	2.14	0.79	1.44
24.0833							17.07	8.26	
24.25								10.93	14.96
24.5					6.48		8.2	6.98	12.08
24.75					10.9				
25	0.38	3.25	6.76	1.38	6.21	1.64	2.32	2.57	9.58
25.333									
25.5						10.54	0.75	0.96	8.32
25.75						4.53			
26	0.45	3.39	4.28	0	1.76	3.88	1.22	0.78	6.96

26.5						3.55			
27	0.54	3.37	1.73	0.27	0.39	1.37	1.91	2.43	5.79
28	0.58	1.68	1.28	0.46	0	1.73	2.28	2	4.79
28.5									
29	1.84	5.81	1.14	0.74	0	2.36	1.07	1.91	4.23
30	0	5.09	1.33	0.9	0.29	2.2	2.08	2.39	3.97
30.5									3.69
31	0.38	3.12	2.08	0	0.63	1.91	1.94	3.72	3.43
32	0	3.09	1.05	0.94	0.86	2.38	1.95	1.93	2.29
33	0.44	2.62	0.93	0.85	1.18	2.82	1.88	1.36	2.32
34	0	1.98	1	1.55	1.21	3.74	2.68	1.43	2.14
35	0	1.81	1.51	1.57	1.5	3.17	2.23	1.68	2.06
36	0	1.9	1.86	2.1	1.65	1.51	2.27	2.27	2.17
37	0.32	1.95	1.72	1.83	1.96	0.59	2.62	2.36	2.03
38	0.46	2.07	1.79	2	1.66	0.6	2.76	1.91	1.49
39	0.5	2.85	1.73	2.27	2.04	1.45	2.46	1.95	2.58
40	1.19	2.56	1.19	2.08	1.45	1.67	0.82	1.99	2.69
41	0.94	2.6	1.56	3.7	1.61	2	1.61	2.39	2.82
42	0	2.3	0.94	2.55	1.73	1.63	2.06	2.89	2.61
43	0.41	1.94	0.96	2.51	2.36	1.52	1.77	2.62	2.55
44	0.47	2.06	1.04	2.37	0.67	1.39	2.35	2.28	2.54
45	0.57	2.03	2.03	2.15	1.32	1.43	2.9	1.94	3.39
46		1.92	2.1	1.28	1.18	1.86	3.13	2.31	3.56
47	0.61	1.93	2.3	1.08	1.66	0.64	2.48	2.33	3.26
48	0.7	2.1	2.66	1.54	1.14	2.37	2.4	2.39	3.08
48.1667								12.04	
48.25					6.82		18.73		11.09
48.42					10.49				
48.5							13.73	9.21	9.65
48.75					6.77				
49	0.3	2.05	5.66	6.5	3.97	2.23	8.47	5.33	5.57
49.33						6			
49.5					2.29	8.98	4.57		2.91
50	0.47	2.45	1.04	3.4	1.11	4.2	2.91	2.02	0.98
50.25								1.6	
50.5							1.56		
51	0.56	2.49	1.33	1.81	1.74	1.51	0.7	0.91	1.11
52	11.45	3.39	2.24	1.26	2.5	1.73	0.45	0.91	1.75
53	11.12	6.01	1.01	1.38	2.19	1.69	0.84	1.82	2.44
54	1.27	5.91	1.9	1.41	2.24	1.94	1.78	2.69	1.86
54.5									
55	0.56	7.22	3.12	0.89	2.23	1.89	1.93	3.62	1.85
56	0.59	8.22	1.62	1.43	1.62	2.13	2.05	2.46	1.15
57	0.7	8.87	2.14	1.04	1.64	2.53	2.47	1.65	1.96

58	0.98	9.33	2.8	1.64	2.87	2.44	2.16	2.1	2.52
59	1.04	10.11	2.34	0.92	2.43	2.29	3.1	3.22	2.39
60	1.26	10.78	2.45	1.01	1.83	1.94	2.94	2.96	2.54
61	1.25	11.2	2.56	1.02	2	1.65	2.79	2.12	2.48
62	1.38	11.33	6.26	1.12	2.07	0.74	2.35	1.75	2.44
62.5						0.94			
63	1.48	11.81	6.01	1.9	1.97	1	2.79	1.65	2.33
64	1.48	12.3	5.89	2.19	2.08	1.62	2.89	1.72	2.43
65		12.45	6.11	1.9	0.9	2.37	1.59	2.2	2.58
66	2.49	12.84	6.72	2.09	2.11	2.45	2.02	2.4	2.74
67	2.63	13.43	6.84	2.49	2.48	2.73	2.43	3.45	3.06
68	2.61	14.11	7.08	3.21	3.41	2.36	2.24	3.1	3.3
68.5									
69	2.56	14.71	8.25	3.45	2.53	2.26	2.6	2.47	3.47
70	2.76	15.42	9.49	4.24	1.89	1.54	2.45	1.92	3.68
71	3.16	15.94	10.86	5.71	1.35	1.73	2.61	1.69	3.32
72	3.12	16.43	11.65	6.58	1.21	2.23	2.19	2.06	3.24
72.083					6.02				
72.1667					7.51			9	
72.25							10.3	13.14	13.12
72.5					4.95		6.94	9.76	11.87
73	3.33	16.54	12.6	7.63	2.57	1.78	3.7	6.27	8.07
73.5					0.48	6.21	2.36		4
73.75						10.39			
74	3.88	16.86	16.18	8.18	1.37	6.1	1.68	2.71	1.47
74.5						2.89		1.96	
75	5.3	16.98	15.33	8.65	1.96	2.17	1.07	1.96	1.52
76	6.53	17.39	12.72	10.02	2.71	1.81	1.26	2.84	1.94
77	6.62	16.68	9.63	10.42	2.23	3.1	2.22	2.41	2.87
78	6.77	17.26		11.23	2.36	2.5	2.42	1.82	2.05
79	7.2	18.37	4.77	10.45	2.4	1.36	1.79	1.9	2.37
80	8.06	19.04	3.27	10.02	1.57	0.81	1.78	1.95	2.31
81	9.04	19.99	2.12	12.8	4.34	0.87	1.84	1.91	2.48
82	9.93	20	0.73	13.66	1.9	1.1	2.31	1.51	2.56
83	10.77	20	1.29	14.3	1.63	1.49	1.8	1.65	2.66
83.5					2.14				
84	11.39	20	0.66	15.91	1.17	1.72	2.22	1.71	3.01
85	13.54	20	5.22	17.56	1.88	2.76	3.6	2.39	
86	15.81	20	2.86	17.85	2.2	1.3	3.23	2.32	3.42
86.5						1.89			
87	17.57	20	2.16	18.88	1.92	1.58	2.62	2.08	
88	17.02	20		18.72	2.35	3.97	2.11	1.77	3.47
89	17.59	20		19.25	2.47	0.54	1.72	2.22	3.37
90	19.04	20		20	2.03	0.62	2.01	2.64	3.14

91	20	20	2.22	1.1	0.96	3.08	3.26
92	20	20	1.62	1.59	3.03	2.87	3.29
93	20	20	1.11	1.97	2.85	2.71	3.55
94	20	20	3.18	2.19	2.82	2.57	3.48
95	20	20	1.99	2.28	2.87	2.41	3.46
96	20	20	5.08	1.12	2.81	2.33	3.51
96.0833					11.07	8.91	
96.1667						12.92	
96.25			7.53		9.27		14.33
96.5			6.54		6.79	10.24	12.26
96.833			5.58				
97	20	20	3.29	2.58	4.23	7.38	9.24
97.25				12.75			
97.5			4.1	10.39	2.05		7.08
98	20	20	2.03	6.32	1.57	4.64	5.7
98.5			2.57			4.41	
99	20	20	1.69	3.85	1.33	4.02	3.52
99.5			1.69	2.76		3.4	
100	20	20	1.28	2.02	1.55	3.19	1.15
101	20		2.35	1.42	1.82	3.46	1.23
101.5						3.26	
102	20		2.55	1.3	2.19	2.94	1.41
102.5						2.28	
103			2.31	2.68	1.96	1.96	1.53
104			1.58	2.72	2.15	1.44	1.76
105			1.02	2.37	3.01	1.42	1.84
106			1.15	2.29	3.71	1.85	1.06
107			1.53	1.89	2.77	2.47	0.51
108			1.33	1.73	1.3	2.7	1.03
109			1.73	2.1	1.07	2.48	1.27
110			1.78	1.65	1.01	2.08	1.13
111			2.29	1.74	1.58	1.96	1.87
112			2.72	1.75	1.67	1.99	2.05
113			2.96	1.64	1.38	2.29	2.1
114			3.07	1.7	1.75	2.49	2.17
115			3.07	1.65	2.8	3.07	2.12
116			2.84	1.87	3.4	2.49	
117			2.8	2.11	2.67	0.55	2.2
117.25						0.34	
118			3.04	2.08	2.45	0.43	2.36
118.0833						13.94	
118.5						4.08	
119			3.2	2.18	2.14	1.6	2.41
120			3.18	1.89	2.18	0.93	2.59

120.083			10.38		
120.25			9.26		12.37
120.5			8.19		10.02
121	4.86	2.17	6.45	1.1	5.66
121.25		7.23			
121.5			4.19		4.74
121.75		5.24			
122		4.8	2.99	1.85	4.32
122.5		3.58	2.32		
123		2.99	1.96	1.64	2.49
124		2.63	1.12	1.19	0.69
125		2.51	1.24	1.28	0.81
126		2.62	1.47	1.54	1.61
127		2.82	2.36	1.53	2.09
128		3.23	2.25	1.52	1.57
129		3.78	2.34	1.01	1.64
130		4.18	2.37	0.58	1.69
131		3.22	2.38	2.37	1.67
131.5		2.56			
132		2.02	2.23	2.76	1.74
132.5		2.24			
133		2.29	1.99	1.61	1.88
133.5		1.68			
134		1.39	1.83	0.53	1.9
135		2.35	2.26	2.31	2.15
136		2.43	2.4	1.57	1.9
136.5		2.84			
137		3.2	2.44	1.27	1.99
138		3.43	2.87	2.07	1.17
139		3.39	3.12	1.96	1.44
140		4.01	2.02	1.39	1.68
141		3.97	1.63	1.39	1.41
142		4.07	2.02	2.02	1.83
143		4.11	2.08	3.2	2.06
144		4.56	2.59	2.57	2.07
144.1				13.27	
144.16			13.5		
144.5			10.13	6.08	
144.75					11.03
145		6.13	6.89	1.86	7.41
145.033				1.38	
145.25				0.34	
145.5					5.53
146		6.57	3.01	1.04	4.19

146.5		1.76		
147	5.63	1.61	1.8	3.19
148	5.3	1.25	2.69	2.76
149	7.5	1.35	1.41	2.41
150	7.15	1.84	1.41	1.88
151	7.17	2.63	1.89	1.61
152	7.16	2.2	1.76	2.55
153	7.03	2.56	0.96	1.83
154	7.4	2.42	1.91	1.87
155	8.86	2.14	0.67	2.17
156	18.43	2.13	2.39	2.02
157		1.25	1.73	2.11
158		0.77	1.24	1.98
159		2.45	0.54	2.13
160		2.83	0.36	2.47
161		3.41	0.33	2.55
162		2.44	1.97	2.41
163		2.23	0	2.7
164		2.36	1.32	2.78
165		1.97	1.51	2.21
165.0833			1.52	
165.5				9.54
166		2.53	3.14	5
166.0833			13.8	
166.5			3.11	4.34
166.75			0.66	
167		2.41	0.66	4.17
168		2.4	1.22	4.54
168.083		10.83		
168.5		8.05		
169		5.86		
170		3.64		
170.5		3.12		
171		2.95		
171.5		2.62		
172		2.49		
172.5		2.24		
173		2.1		
173.5		1.87		
174		1.63		
175		2.04		
176		1.76		
177		1.73		
178		2.01		

179	2.29
180	1.96
181	1.68
182	2.4
183	2.43
184	2.42

Figure 5 **pH**

Time (Hours)	1	2	3	4	5	6	7	8	9
0	7.413	6.824	6.695	7.676	7.224	7.411	7.187	6.503	6.986
1	7.211	7.261	7.125	7.704	7.334	7.558	7.07	7.273	7.279
2	7.383	7.33	7.215	7.63	7.339	7.562	7.243	7.348	7.346
3	7.373	7.317	7.156	7.693	7.519	7.517	7.249	7.298	7.281
4	7.351	7.263	7.123	7.826	7.604	7.706	7.181	7.385	7.223
5	7.33	7.27	7.135	7.881	7.544	7.74	7.2	7.433	7.209
6	7.289	7.276	7.133	7.918	7.492	7.731	7.191	7.456	7.37
7	7.244	7.275	7.11	7.884	7.455	7.631	7.18	7.483	7.554
8	7.202	7.266	7.105	7.874	7.439	7.545	7.151	7.481	7.59
9	7.175	7.178	7.092	7.821	7.433	7.655	7.19	7.511	7.573
10	7.122	7.241	7.063	7.874	7.416	7.503	7.302	7.498	7.453
11	7.078		7.066	7.86	7.43	7.445	7.354	7.476	7.425
12	7.031	7.221	7.03	7.838	7.428	7.307	7.336	7.529	7.367
13	6.944	7.18	6.991	7.781	7.401	7.321	7.342	7.568	7.399
14	7.508	7.151	7.976	7.826	7.391	7.255	7.372	7.583	7.423
15	6.767		6.924	7.84	7.398	7.253	7.349	7.586	7.442
16	6.936	7.132	6.755	7.862	7.43	7.639	7.359	7.508	7.457
17	6.908	7.117	7.053	7.859	7.421	7.793	7.414	7.379	7.462
18	7.014	7.102	6.707	7.891	7.446	7.833	7.404	7.279	7.478
19	6.995	7.134	7.13	7.892	7.47	7.828	7.358	7.353	7.486
20	7.008	7.118	7.123	7.88	7.501	7.848	7.382	7.429	7.471
21	7.028	7.127	7.069	7.934	7.515	7.719	7.403	7.463	7.468
22	7.041	7.123	7.108	7.897	7.499	7.691	7.416	7.491	7.469
23	7.026	7.107	7.086	7.85	7.473	7.777	7.427	7.549	7.418
24	7.054	7.083	7.032	7.841	7.469	7.662	7.39	7.455	7.413
25	7.086	7.061	7.027	8	7.545	7.683	7.428	7.52	7.526
26	7.114	7.067	6.9	7.954	7.406	7.608	7.518	7.253	7.342
27	7.117	7.072	7.116	8	7.33	7.44	7.472	7.375	7.258
28	7.062	7.116	7.044	8	7.335	7.333	7.477	7.398	7.407
29	7.066	7.08	6.841	8	7.31	7.498	7.476	7.392	7.471
30	7.074	7.233	6.931	8	7.338	7.549	7.46	7.343	7.483
31	7.017	7.182	7.007	8	7.331	7.433	7.465	7.374	7.433
32	7.654	7.152	6.952	8	7.368	7.423	7.432	7.394	7.407
33	7.714	7.124	7.009	8	7.361	7.46	7.409	7.382	7.402
34	7.641	7.082	6.974	8	7.392	7.483	7.381	7.317	7.411
35	7.528	7.186	6.973	8	7.323	7.56	7.329	7.364	7.447
36	7.457	7.173	6.997	8	7.313	7.565	7.399	7.357	7.439
37	7.399	7.162	6.827	8	7.27	7.642	7.432	7.401	7.437
38	7.369	7.133	7.008	8	7.278	7.606	7.449	7.436	7.43
39	7.318	7.12	6.943	7.974	7.277	7.567	7.486	7.429	7.466
40	7.269	7.118	7.094	7.922	7.454	7.563	7.476	7.397	7.491

41	7.229	7.098	7.005	7.833	7.441	7.527	7.504	7.351	7.504
42	7.227	7.048	7	7.611	7.423	8	7.388	7.407	7.502
43	7.179	7.032	6.999	7.301	7.41	7.505	7.35	7.409	7.497
44	7.15	7.064	7.038	7.198	7.402	7.456	7.319	7.406	7.5
45	7.156	7.058	7.039	7.525	7.368	7.476	7.335	7.413	7.489
46	7.139	7.032	7.012	7.543	7.366	7.467	7.345	7.45	7.485
47	7.101	7.077	7.037	7.59	7.369	7.465	7.337	7.396	7.457
48	7.118	7.079	6.982	7.604	7.404	7.442	7.286	7.416	7.455
49	7.126	7.036	6.989	7.647	7.45	7.572	7.412	7.486	7.538
50	7.073	7.049	7.081	7.468	7.43	7.469	7.566	7.409	7.463
51	7.111	7.039	6.997	7.439	7.428	7.388	7.463	7.35	7.361
52	7.042	6.665	7	7.391	7.408	7.334	7.434	7.332	7.313
53	6.993	6.732	6.893	7.348	7.441	7.324	7.455	7.344	7.277
54	7.014	7.055	6.961	7.358	7.422	7.413	7.46	7.353	7.401
55		7.146	6.987	7.293	7.433	7.426	7.411	7.362	7.43
56		7.115	7.025	7.417	7.396	7.444	7.414	7.373	7.43
57		7.091	7.003	7.442	7.378	7.486	7.555	7.408	7.443
58		7.159	6.977	7.434	7.393	7.443	7.611	7.347	7.433
59		7.134	7.01	7.386	7.392	7.45	7.624	7.355	7.369
60		7.104	7.004	7.291	7.392	7.465	7.611	7.393	7.358
61		7.083	6.987	7.399	7.397	7.461	7.365	7.39	7.367
62		7.144	7.044	7.124	7.404	7.475	7.259	7.393	7.362
63		7.123	6.999	7.198	7.377	7.473	7.436	7.491	7.372
64		7.11	7.013	7.289	7.366	7.485	7.45	7.482	7.399
65		7.069	7.01	7.294	7.393	7.457	7.451	7.431	7.4
66		7.085	6.969	7.303	7.395	7.467	7.412	7.434	7.395
67		7.058	6.99	7.325	7.39	7.48	7.376	7.442	7.366
68		7.062	7.027	7.355	7.342	7.456	7.361	7.439	7.36
69		7.056	7.029	7.335	7.359	7.461	7.233	7.392	7.37
70		7.037	7.008	7.346	7.332	7.448	7.325	7.409	7.35
71		7.037	6.968	7.357	7.278	7.44	7.327	7.435	7.392
72		7.047	7.001	7.347	7.392	7.417	7.307	7.466	7.436
73		7.084	6.997	7.354	7.267	7.485	7.315	7.48	7.426
74		7.113	7.008	7.291	7.309	7.477	7.559	7.416	7.345
75		6.974	6.986	7.093	7.364	7.457	7.508	7.382	7.261
76		7.013	6.969	6.947	7.345	7.409	7.481	7.353	7.328
77		7.072	7.107	6.906	7.374	7.392	7.372	7.326	7.357
78		7.109	7.039		7.332	7.418	7.353	7.321	7.355
79		7.058	6.994	6.934	7.379	7.446	7.345	7.342	7.351
80		7.072	6.981	6.93	7.421	7.422	7.39	7.369	7.334
81		7.089	6.971	6.929	7.348	7.39	7.411	7.387	7.333
82		7.086	6.978	6.918	7.327	7.362	7.476	7.386	7.363
83		7.111	6.972	6.787	7.231	7.329	7.484	7.41	7.368
84		7.115	7.037	6.878	7.183	7.35	7.497	7.385	7.359

85	7.062	6.996	6.884	7.148	7.353	7.5	7.38	7.34
86	7.024	7.078	6.822	7.125	7.371	7.48	7.398	7.333
87	7.025	7.006	7.018	7.158	7.381	7.497	7.417	7.351
88	7.011	6.962		7.129	7.374	7.282	7.382	7.358
89	6.997	7		7.113	7.368	7.33	7.392	7.372
90	7.006	6.986		6.885	7.353	7.34	7.396	7.368
91	6.935	7.015			7.309	7.374	7.386	7.347
92	6.917	6.966			7.312	7.417	7.344	7.347
93	6.9	6.993			7.284	7.435	7.394	7.338
94	6.87	6.989			7.53	7.441	7.391	7.326
95	6.793	6.986			7.571	7.436	7.391	7.32
96	6.717	6.991			7.499	7.412	7.393	7.313
97	6.538	6.992			7.386	7.455	7.439	7.421
98	6.606	6.964			7.332	7.521	7.428	7.435
99	6.382	6.971			7.304	7.48	7.36	7.409
100	6.39	6.941			7.27	7.483	7.356	7.427
101	6.564				7.396	7.444	7.359	7.396
102	6.555				7.412	7.363	7.33	7.377
103					7.376	7.325	7.374	7.385
104					7.376	7.304	7.4	7.344
105					7.364	7.265	7.408	7.346
106					7.369	7.242	7.384	7.339
107					7.318	7.243	7.368	7.349
108					7.345	7.226	7.364	7.321
109					7.327	7.339	7.354	7.312
110					7.313	7.359	7.364	7.312
111					7.249	7.373	7.375	7.323
112					7.22	7.397	7.393	7.338
113					7.207	7.386	7.4	7.336
114					7.357	7.392	7.377	7.315
115					7.345	7.378	7.418	7.294
116					7.308	7.389	7.403	7.299
117					7.261	7.406	7.42	7.275
118					7.315	7.378	7.408	7.241
119					7.325	7.367	7.409	7.539
120					7.279	7.369	7.394	7.363
121					7.288	7.368	7.476	7.338
122						7.39	7.467	7.321
123						7.397	7.425	7.346
124						7.369	7.387	7.385
125						7.349	7.376	7.405
126						7.318	7.368	7.414
127						7.276	7.37	7.422
128						7.337	7.39	7.412

129	7.331	7.387	7.44
130	7.328	7.382	7.502
131	7.332	7.376	7.375
132	7.367	7.386	7.361
133	7.34	7.365	7.432
134	7.365	7.365	7.438
135	7.369	7.377	7.348
136	7.388	7.376	7.345
137	7.359	7.39	7.376
138	7.356	7.39	7.323
139	7.376	7.345	7.329
140	7.344	7.352	7.311
141	7.325	7.335	7.347
142	7.321	7.303	7.349
143	7.319	7.322	7.422
144	7.307	7.348	7.37
145		7.357	7.514
146	7.236	7.35	7.217
147	7.18	7.311	7.247
148	7.264	7.329	7.421
149	7.21	7.338	7.532
150	7.24	7.336	7.549
151	7.254	7.346	7.515
152	7.235	7.342	7.475
153	7.24	7.363	7.49
154	7.266	7.349	7.5
155	7.259	7.353	7.482
156	6.664	7.344	7.447
157		7.317	7.446
158		7.28	7.442
159		7.289	7.401
160		7.295	7.4
161		7.405	7.386
162		7.437	7.384
163		7.416	7.394
164		7.389	7.421
165		7.404	7.428
166		7.406	7.365
167		7.428	7.473
168		7.425	7.396

10
7.096
6.921
6.846
6.848
6.899
6.915
6.907
6.894
6.921
7.122
7.257
7.332
7.362
7.378
7.391
7.398
7.397
7.411
7.418
7.427
7.39
7.417
7.428
7.431
7.416
7.438
7.428
7.413
7.384
7.354
7.34
7.329
7.298
7.343
7.365
7.39
7.397
7.401
7.415
7.392
7.394

7.397
7.409
7.43
7.424
7.386
7.423
7.434
7.442
7.521
7.508
7.34
7.26
7.326
7.341
7.359
7.367
7.359
7.345
7.332
7.349
7.351
7.319
7.338
7.339
7.341
7.334
7.326
7.328
7.3
7.294
7.35
7.37
7.458
7.484
7.378
7.377
7.429
7.426
7.42
7.4
7.399
7.401
7.407
7.422

7.412
7.43
7.424
7.438
7.451
7.425
7.41
7.398
7.397
7.414
7.43
7.488
7.529
7.506
7.495
7.409
7.404
7.415
7.452
7.424
7.439
7.393
7.4
7.438
7.443
7.445
7.445
7.481
7.483
7.467

7.428
7.413
7.413
7.413
7.549
7.554
7.536
7.423
7.427
7.452
7.477
7.482

7.483
7.482
7.492
7.478
7.449
7.455
7.44
7.458
7.46
7.48
7.482
7.464
7.47
7.487
7.467
7.472
7.524
7.523
7.493
7.503
7.493
7.492
7.483
7.411
7.443
7.45
7.433
7.373
7.378
7.365
7.365
7.375
7.352
7.352
7.353
7.326
7.361
7.42
7.408
7.348

Figure 5 **Sodium (mmol/L)**

Time	1	A	B	C	D	1	2	3	4
0		123.3	137.7	142	145.9	145.6	144	113	135.2
1	135.1	143	121	131.6	128.3	134.5	119.8	119.8	120.1
2	137.3	146	125.7	136	131	136.5	119	120.7	121.9
3	139.8	148.1	125.7	136.9	135.2	137.2	120.6	122.5	125.3
4	140.2	151.3	126.2	138.3	136.6	139.3	124.8	128.9	126.6
5	142.8	151.6	126.6	138.9	138.2	138.9	125.8	133	127.3
6		153.2	127.2	138.7	137.7	139.3	126	135.5	130.1
7	144.3	155.4	128.1	140	138.9	139.1	126.7	138.1	132.6
8	144.8	155.6	129.1	140.6	139.7	140	126.1	140.6	134.6
9	146.5	148.1	130	140.8	140.6	146	129.7	142.4	136
10	146.4	157.6	132.3	141.3	141.3	147.8	131.5	141.2	138.4
11	146.5		131.7	139.8	142.1	149.9	133.3	142.6	141.4
12	148.1	159.1	130.9	139.3	142.2	148.3	134	145.3	141.5
13	150.1	167.1	132.3	141.2	141.4	148.2	135.2	147.4	143.9
14	203.4	165.3		141	142.7	146	135.8	148.3	146.3
15	146.5			139.9	143	147.3	136.3	149	148.5
16	153.2	168.3		139.1	144	149.6	137.1	148.2	149.7
17	154.2	168.5		138.5	144.3	145.5	137.2	149.7	151.3
18	156.5	169.1		139.8	145.9	146.6	137.5	149.2	152.4
19	160.9	168	154.7	140.4	147.4	145.8	138.1	145.9	152.1
20	164.9	169.8	150.7	139.9	149.2	148.1	138.2	148.8	151.9
21	167.5	171.6		139.9	150.7	144.7	138.6	150.9	150.3
22	167.3	170.9		139.2	151.3	143.7	139.2	153.1	150.3
23	166.8	174.1	148	139.3	149.3	144.3	139.5	152.7	14.94
24	168.7	175.1	153.1	139.4	150.4	144.5	138.8	153.1	147
25	172.5	178.6	154.5	154	159.4	156.7	139.6	166.2	155.5
26	173.5	180.6	138.8	158.9	155.1	161.2	146	164.3	152.7
27	178.6	176.6	158.1	153.8	151.3	158.3	143.9	161.1	153
28	177.3	182.7	157.5	148.5	150.2	155.4	143.3	158.5	150.6
29	180.1	186.6	150.5	146.3	149.1	150.7	143	156.1	149.7
30	179.8	181.4	151.9	143.6	150	148.5	141.9	156.7	149.4
31	163.6	181.8	153.1	136	150.2	148	140.4	155.6	150.2
32	169	187.1	154.4	140.9	154	150.9	139.2	156.4	148.1
33	169.4	187.4	156.7	140.5	152.8	153	139	156.4	145.6
34	170.3	191.5	157	141.1	153.3	153.2	138.6	153.4	144.8
35	171.7	193.3	154.6	140.6	153.5	155.2	140.2	154.4	146.2
36	172.1	194.7	156.6	140.9	154.6	155.4	138.4	153.9	146.5
37	171.6	195.9	127.2	142	154.2	157.4	138.9	155.6	147.7
38	172.4	197.6	159.7	141.8	153.3	156.9	137.9	154.4	146.8
39	171.9	198	159.9	142.2	152.1	155.9	140.1	154.3	148.5
40	172.5	198.4	168.6	142.8	150.8	154.8	140.9	151.8	148.5

41	173.2	198.6	162.8	143.2	148	153.3	141.8	150.6	149.5
42	173	204.6	164.2	143.7	145.2	153.1	143.3	152.1	149.9
43	173.8	204.5	164.5	143.4	143	153	145.4	151.6	149.5
44	173.5	203		144.1	141.5	149	145.3	154	148.8
45	173.9	205.4	166.3	145.3	139.9	149.1	145.7	154.2	148.4
46	172.5		166.5	146.7	139.5	148.1	146.9	152.7	148.3
47	174.6	206.6	168.4	148.9	139	147.8	147	150.7	146.8
48	177	206.6		150	139.4	147.1	146.9	149.7	146.1
49	179.7	210.4		159.2	146.8	154	147.6	165	153.8
50	180.4	209.9	169.5	157.2	147.2	153.6	155.6	166.4	155
51	182.5	209	171.8	154.6	147	152.2	155.6	161.2	151.8
52	184.6	169.3	172.3	153.2	146.1	151.4	154.5	154.4	148.3
53	180.8	171.3	158.3	149.6	146.5	151.3	151.9	152.1	147
54	183.3	174.8	162.2	147.2	146.5	151.3	151.2	153.7	146.6
55		177.9	163.4	144.5	145.4	150.7	149.9	152.9	148.2
56		178.2	164	141.3	143.8	149.9	150.4	154.7	147.2
57		179.5	166.6	141.3	142.5	150.6	149.1	156.8	145.9
58		177.6	167.3	141.4	142.9	149.9	148.3	152.6	145.1
59		183.3	168	139.6	141.2	148.5	148.1	154.8	144.1
60		185	167.2	138.8	141.2	152.4	147.2	155.2	143.6
61		185.6	168.4	138.3	141	150.9	148	154	142.9
62		188.9		136	141.4	151.8	146.7	154	140.3
63		189	170.5	136.2	141.6	152	145	152.6	141.4
64		189.3		140.3	142	152.3	144.1	152.1	141.6
65		187.4	172.9	136.1	140.5	150.9	143.2	147.7	141.7
66		189.5	172.4	136.5		152.1	141.9	150.2	141.1
67		190	174.5	135.9		152.1	141.8	150.1	142.9
68		191.8	177.3	136.3		153.5	139.6	150.7	142.4
69		195.1	177.9	136.3	138.6	152.9	137.9	150.2	141.9
70		194.9		135.5	139	152.3	137.9	149.7	140.6
71		196.5	178.3	135.5	138.7	152	138.1	149.7	141.1
72		197.5	176.8	135.9	138.6	150.1	141.1	151.9	141.3
73		198.9	176.4	133.7	131.4	155.8	140.9	160.6	151.4
74		201.6	179.6	132.9	133.9	157.6	152.5	161.3	152
75		176.1	179.2	134.6	136.5	155.8	153.8	154.7	150.6
76		174.8	181.8	134.4	137.2	154.3	154.8	149	147.2
77		177.6	167.6	135.8	137.7	154	144.6	146.6	144.5
78		179.8	168.5	134.6	135.5	156.4	144.4	146.9	142.9
79		180.8	167.6	135.1	137.5	157.7	142.3	145.2	140.7
80		183.4	170.2	137.7	137.1	156.5	139.8	146.5	142.4
81		185	170	138.7	136.9	155.8	140.5	147	141
82		186.3	171.8	139.1	137.2	151.4	141.1	147.6	141
83		189.7	172	139.2	137.7		142.2	148.3	140.1
84		188.8	175.7	140.5	138.1	148.3	143.5	146.5	139.8

85		190.9		148.7	137.8	148.3	146.9	150.3	139.3
86		194.5	184.3	152.1	137.6	148.9	144.6	150.1	138.3
87		196.4	178.9	156.6	137.7	150	144.1	150.2	138.5
88		198.1	177.4		137.5	150.5	145.7	148	139.3
89		198.7	177.3		138.5	150.1	143.6	147.3	139.9
90		198.3	179.3		138.2	149.5	143.5	146.2	139.7
91		202.5	185.6			149.3	145.7	145.4	140.3
92		202.6	184.5			148.2	147.9	145.6	139.2
93		203.9	187.6			148.1	149.5	147.2	138.4
94		205	182.7			146.5	151	146.4	138.1
95		205.8	187.7			146.5	150.8	146.7	138.3
96		209.2	184.9			144.9	149.4	145.9	138.1
97		214.3	187.2			148.3	150.3	152.2	145.3
98		201.1	189.2			148.9	162.3	152	147.1
99		222.9	189.5			148.9	162.7	147.7	146.3
100		231.5	189.1			147.5	163.3	145.9	146.7
101		182.1				146.1	16.8	145.5	147.1
102		194.5				144.3	150.7	145.3	146.6
103						142.3	147.9	145.1	146.2
104						141	146	146.5	143.5
105						139.8	144.4	145.8	141.6
106						140.3	142	147.4	141.4
107						139.7	141.3	145.3	140.7
108						140.4	140.9	143.1	139.8
109						142.2	141.3	143.3	138.6
110						141.2	140.5	143.1	139.3
111						141.4	140.1	144.2	138.6
112						141.1	143.4	145.2	137.4
113						143.1	141.1	144.9	138
114						139.8	141	144.6	137.9
115						139.2	140.9	145.8	138.3
116						138.6	142.2	146.5	137.2
117						136.9	142	147.1	139
118						136.5	141.8	144.9	138.5
119						136.3	142.7	145.7	148.8
120						135.6	141.3	145.5	146.2
121						134.2	141.8	154.6	144
122							147.3	154.9	143
123							147.4	155.1	142
124							148.1	149.6	141.1
125							147.9	147.8	141.6
126							148.4	147	141.7
127							148.6	145.9	142.3
128							147.6	146.3	140.3

129	148	146.7	140.3
130	149.1	146.2	140.9
131	148.5	146.5	143.3
132	147.7	146.3	143.3
133	148.2	145.9	140.9
134	146.5	144.6	141.1
135	145.4	145.3	142.5
136	143.9	145.3	142.4
137	141.9	146.2	142.2
138	141.3	145.9	140.9
139	140.9	145.4	142.6
140	140.5	144.2	144
141	139	143	143.2
142	138.5	143.3	143.7
143	138.5	144.2	146.2
144	138.7	146.5	146
145	137.8	156.6	153
146	138.4	157.2	151
147	138.8	154.6	149.6
148	138.6	150.5	146.2
149	137.8	147.7	143
150	137.6	147.3	142.6
151	137.8	146.7	144
152	137.9	146.5	144.5
153	138.6	147.1	145.4
154	138.3	146.6	144.7
155	137.8	146.2	144.7
156	136.1	146.2	147.3
157		144.5	145.8
158		142.8	146.1
159		144.3	141.6
160		145.7	143.1
161		145.4	143.3
162		144	144.5
163		143.1	143.1
164		142.9	147.9
165		142.2	151.1
166		142.2	152.3
167		143.5	158.6
168		143.2	159.8

5
136.2
96.7
102.9
105.4
107.5
108.8
110.8
112.4
115.1
121
125.5
128.3
131.8
134.6
137.7
138.8
139.6
140.1
141.4
140.6
140.8
140.8
140.3
141.4
139.3
149
149.4
147.5
147.4
146.6
146.3
145
144.3
141.9
140.3
138.4
139.7
138.2
138.3
138.2
138.1

137.7
137.6
135.8
135.7
136.4
135.7
136.5
135.4
141.8
141.5
141.6
140.9
139.4
137.5
137
136
135.8
136.6
135.8
139.3
138.2
139.1
138.7
137.5
136.9
136.3
136.3
135.3
136.7
135.6
134.4
135.5
141.6
142.2
142.2
140.1
140.2
138.8
137.3
136.3
135.4
134.1
133.7

136.4
138
144.3
144.2
144.5
143.7
142.7
140.5
138.1
137.3
137
135.2
136.8

139.7
137.5
138.3
138.3
146.1
146.7
146.9
145.9
142.9
142.8
142.2
141.5

141
140.7
140.3
139.3
139.4
139.2
139.4
139.1
139.5
138.2
138.3
139.6
140.1
141
125.7
133.7
143.5
146.1
146.1
147.3
147.9
145.2
143.4
141.6
139.9
141.3
141.4
140.2
139.6
140.1
139.6
139.6
138.4
138.8
138.8
139
137.5
145.2
145.6
146.1

Figure 5 Potassium (mmol/L)

Time	A	B	C	D	1	2	3	4	5
0	20	8.89	7.63	8.06	7.22	9.64	20	10.75	12.75
1	9.48	20	13.62	15.05	12.17	11.42	13.61	9.86	20
2	7.77	20	9.96	12.31	10.7	12	12	7.41	18.31
3	7.44	20	7	7.86	9.28	10.14	10.79	4.86	17.13
4	5.86	20	5.68	6.39	7.19	6.45	7.2	4.2	16.31
5	6.56	20	5.14	5.69	5.67	5.42	5.54	4.16	15.38
6	7.01	20	4.99	5.51	5	6.08	5.04	3.95	14.25
7	7.4	20	4.26	5.27	4.85	5.94	4.68	4.15	13.34
8	7.36	20	3.96	4.95	4.37	6.09	4.67	4.03	12.48
9	7.47	20	4.13	4.76	3.77	5.28	5.18	4.6	9.96
10	8.19	20	4.2	4.73	3.98	6.52	4.9	3.68	7.61
11		20	5.12	4.66	3.68	5.6	4.68	3.87	6.57
12	8.07	20	5.65	4.77	4.53	5.09	4.57	4.03	5.7
13	8.59	20	4.44	4.43	4.55	4.64	4.47	3.68	5.17
14	8.88	14.85	4.39	4.21	3.99	4.27	4.6	3.63	4.74
15			4.19	4.4	3.83	3.99	4.33	3.77	4.45
16	10.46		5.23	4.41	3.86	3.85	4.19	3.77	4.16
17	10.62		5.53	4.48	4.25	3.83	4.17	3.45	4
18	11.02		5.48	4.34	4.19	3.83	4.87	3.44	4.07
19	13.43	20	4.53	4.18	4.03	3.84	4.68	4	3.95
20	12.91	20	4.47	4.05	3.95	3.82	4.3	3.64	4.9
21	13.19		4.45	4.12	4.4	3.77	4.07	3.92	4.74
22	13.87	20	4.81	4.02	4.01	3.84	4.06	4.17	4.5
23	14.63	20	4.82	4.53	4.07	3.88	4.11	4.16	4.36
24	15.39	20	5.14	3.94	4.3	4.79	3.98	4.4	4.78
25	17.28	20	4.28	3.5	3.49	4.53	3.08	3.67	4.19
26	18.1	20	5.23	3.76	3.51	3.99	3.29	3.75	4.46
27	18.98	20	5.82	3.95	3.51	3.97	3.78	3.44	4.77
28	19.1	20	5.89	4	4.17	4.49	3.84	4.51	4.75
29	18.52	20	6.12	3.87	4.9	4.51	4.11	5.09	4.6
30		20	6.26	3.57	5.17	4.91	3.42	4.88	4.54
31		20	6.1	3.68	5.09	4.93	3.98	3.37	5.16
32	18.18	20	6.28	3.67	4.44	5.32	4.06	3.51	5.21
33	18.45	20	6.04	3.79	3.62	5.34	3.81	3.97	5.21
34	20	20	5.81	3.82	3.54	5.3	4.8	4.48	4.72
35	20	20	5.6	3.08	3.29	3.21	4.08	4.11	4.75
36	20	20	5.59	2.9	3.4	4.34	4.32	3.96	4.24
37	20	16.43	5.45	2.94	3.68	4.77	4.18	3.35	4.72
38	20	20	5.32	3.65	3.57	4.85	4.14	3.63	4.28
39	20	20	5.07	3.91	3.6	4.59	4.08	3.78	3.9
40	20	20	5.03	3.81	3.72	4.39	4.19	4.17	3.85

41	20	20	5.03	4.97	3.76	4.4	3.51	4.05	3.95
42	20	20	4.67	4.94	3.7	3.99	3.92	3.57	4.44
43	20	20	4.53	4.89	3.81	3.95	3.84	3.58	5
44	20	20	4.49	4.81	3.76	4.12	3.74	3.9	4.72
45	20	20	4.12	5.46	3.62	4.25	3.69	3.95	4.03
46	20	20	3.63	4.66	3.51	3.85	4.34	3.91	5.14
47	20	20	3.78	4.37	3.37	4.2	4.95	3.9	4.62
48	20	20	3.74	4.4	3.52	5.19	5.22	4.04	5.41
49	20	20	3.39	4.26	3.38	4.91	2.6	3.31	4.61
50	20	20	3.64	4.68	3.38	4.64	2.01	3.22	4.33
51	20	20	3.47	4.92	3.58	4.28	2.55	3.33	3.56
52	20	20	3.65	4.47	3.97	3.62	3.61	3.51	3.41
53	20	16.66	3.67	4.68	3.96	4.43	3.96	3.78	4.17
54	20	16.21	4.12	4.17	3.72	4.51	3.13	4.14	4.51
55	20	15.24	5.66	3.95	3.83	5.03	3.59	3.62	4.37
56	20	15.7	5.83	4.77	3.67	4.93	3.68	3.81	4.65
57	20	15.56	5.63	4.6	3.77	5.02	3.98	4.52	4.42
58	20	16.23	5.73	4.56	3.82	4.98	3.92	3.93	4.22
59	20	16.47	5.91	4.59	4.11	4.81	3.06	3.51	3.88
60	20	16.69	6.02	4.58	4.29	4.61	3.34	3.66	3.8
61	20	17.11	5.94	4.33	3.9	3.61	3.77	3.84	4.92
62	20	16.98	7.99	4.27	3.83	3.32	3.87	3.9	3.63
63	20	17.76	7	4.73	3.74	3.82	4.28	4.02	3.97
64	20	18.48	6.55	4.5	3.64	4.55	3.67	4.36	4.17
65	20	18.56	6.12	4.87	3.76	4.66	4.47	4.4	4.18
66	20	18.86	5.97		3.97	4.87	3.85	4.38	4.19
67	20	18.6	5.76		4.21	4.19	3.76	3.68	4.1
68	20	18.8	5.87		3.61	3.9	3.74	3.73	4.27
69	20	19.07	6.02	5.02	3.64	6.35	3.97	3.8	3.67
70	20	19.42	6.25	5.07	3.72	6.2	4.02	3.97	4.42
71	20	20	6.58	5.71	3.6	5.19	4.37	4.23	4.34
72	20	20	7.01	5.73	3.62	2.79	5.68	5	4.38
73	20	20	7.54	5.06	3.37	2.74	3.77	3.22	5.02
74	20	20	8.86	4.59	3.53	3.09	2.92	3.18	4.48
75	20	20	8.95	4.33	3.43	2.76	3.64	2.55	3.13
76	20	20	8.51	4.47	3.74	1.61	4.33	3.32	3.5
77	20	16.85	8.12	4.56	3.15	1.78	3.68	4.02	3.62
78	20	17.3	7.14	4.51	2.93	1.56	3.74	4.14	4.14
79	20	17.65	6.92	4.01	3.05	2.07	4.21	4.1	4.06
80	20	18.19	6.46	4.75	3.17	3.31	4.02	3.75	3.78
81	20	18.49	5.87	5.31	3.31	3.29	3.9	3.79	3.71
82	20	18.9	5.41	5.45	3.33	4.21	3.77	3.78	4.73
83	20	18.76	5.04	5.19		4.44	3.84	4.33	4.64
84	20	19.37	5.23	4.89	3.72	4.43	4.12	4.36	

85	20	19.82	5.75	4.38	3.8	3.88	2.82	3.75		
86	20	19.81	6.6	4.32	3.6	3.69	3.7	3.91		
87	20	20	7.58	4.54	3.65	3.65	3.62	4.01		
88	20	20		4.79	3.78	4.52	3.93	4.38		
89	20	20		4.75	3.8	4.84	4.09	4.41		
90	20	20		5.11	3.7	4.12	4.34	4.2		
91	20	20			3.46	3.21	4.52	4.04		
92	20	20			3.4	3.14	4.52	3.9		
93	20	20			3.42	2.81	4.52	3.87		
94	20	20			3.61	2.75	4.2	3.93		
95	20	20			3.65	2.91	4.37	4.03	4.75	
96	20	20			3.89	3.36	4.15	4.04	4.41	
97	20	20			3.62	3.52	3.94	3.63	3.74	
98	20	20			3.64	2.23	4.02	3.43	4.26	
99	20	20			3.33	1.81	3.7	3.58	4.84	
100	20	20			3.3	2.11	4.06	3.64	4.53	
101	20				3.63	2.58	3.63	3.33	4.05	
102	20				3.62	2.99	4.08	2.9	4.07	
103					4.15	4.01	4.1	3.32	4.38	
104					4.3	4.21	4.5	4.24	5.32	
105					4.21	3.93	4.66	4.23	4.16	
106					3.59	3.92	3.95	4.18	5.26	
107					3.52	3.83	3.78	4.15	3.84	
108					3.73	3.87	3.74	3.86		
109					4.28	3.91	3.9	3.98		
110					4.04	4.08	3.97	4		
111					3.92	4.16	3.79	4.14		
112					3.81	5.29	3.69	4.46		
113					4.43	3.91	3.72	4.28		
114					4.09	4.19	4.01	4.15		
115					4.86	3.87	3.85	4		
116					4.74	3.78	4.23	4.42		
117					5.95	4.13	4.22	3.25	4.43	
118					5.77	3.72	4.13	3.22	4.96	
119					6	3.85	4.02	3.14	4.67	
120					6.33	3.75	4.04	3.25	4.22	
121					7.27	4.1	3.61	3.91	4.22	
122						2.79	3.32	3.98	4.54	
123						2.98	2.76	4.47	5.23	
124						3.07	3.45	4.83	3.37	
125						3.13	3.25	4.76	4.22	
126						3.4	3.49	4.54	3.79	
127						2.95	3.54	4.3	3.95	
128						2.81	3.44	5.74	4.02	

129	2.65	3.71	5.4	3.87
130	3.07	3.46	5.53	4.12
131	2.49	3.44	3.3	4.11
132	2.74	3.62	3.48	4.07
133	2.81	3.51	4.46	5.45
134	2.99	3.64	4.15	5.1
135	3.37	3.76	3.48	4.63
136	3.65	3.93	3.74	4.27
137	3.71	4.23	4.29	4.2
138	3.97	4.11	4.64	4.88
139	4.2	3.72	3.94	5.36
140	3.8	3.94	3.37	4.55
141	3.99	4.02	4.09	3.45
142	3.95	3.46	3.69	4.53
143	4.08	4.16	4.4	4.67
144	4.15	4.41	3.69	4.83
145	4.4	1.99	3.63	2.47
146	4.1	1.93	2.44	1.91
147	4.28	2.23	2.93	1.79
148	4.34	2.8	4.69	1.81
149	6.19	3.29	5.28	1.85
150	5.81	3.4	5.1	2.72
151	5.33	3.45	4.24	3.19
152	4.64	3.74	3.8	3.92
153	4.15	3.66	4.17	5.14
154	4.32	3.75	3.98	4.2
155	4.65	4	3.85	4.03
156	10.16	3.73	3.48	4.1
157		3.69	3.31	4.06
158		3.82	3.32	3.56
159		3.47	4.37	3.56
160		3.4	4.12	3.62
161		3.6	3.62	3.48
162		4.27	3.98	3.54
163		3.77	3.86	4.18
164		4.61	3.1	3.91
165		4.21	2.73	5.04
166		4.03	3.51	3.59
167		4.15	3.22	3.49
168		3.92	3.45	3.47

Figure 5 Chloride (mmol/L)

Time	A	B	C	D	1	2	3	4	5
0	114.4	115.3	118	117.7	120	119.1	109.8	111.9	115.1
1	111.2	98.7	107.8	100.7	107.9	88.7	92.9	91.5	79.4
2	111	100.3	108.4	100.7	108.4	89.1	93.1	91	80.8
3	111.7	101.8	106.8	103.7	108.3	89	92.8	91.9	81.6
4	112.6	103.2	106.6	104.8	108.2	89.4	97	92.6	81.8
5	115.2	104.2	105.8	105.1	105.4	89.3	99.7	92	82.3
6	116.1	105.3	105.5	102.4	103.8	89.8	101.4	96	82.4
7	117.6	105.6	105.9	104.3	103.6	90.1	101.9	97.8	81.6
8	119.5	105.8	105.8	104.7	102.4	89.1	102.9	99.9	82.1
9	111.6	106.8	105.5	105.5	103.1	93.8	104	100.9	88
10	118.7	107.3	106.4	104.7	104	98.5	104.4	100.4	93.4
11		107.2	104.4	104.8	104.1	100.8	104.7	101.4	95.3
12	122	109.3	104.6	104.7	105.4	101.2	104.6	102.1	99.3
13	124.7	108.1	104.3	104.2	105	101.6	105.9	102.7	100
14	124.8	142.2	104.3	105.1	103.3	102.3	106.1	107.2	101.3
15			103.1	104.6	104.2	102.4	105.6	104.5	102.3
16	128.8		102.9	104.6	105.7	101.7	105.1	103.8	103
17	128.4		103	105	105.5	102.4	106.2	105.2	103.3
18	128.6		103.6	106.3	105.4	102.1	107	105.5	103.6
19	129.8	115.5	102.6	105.8	103.3	101.4	105.8	104.5	103.3
20	131.3	112.2	102.4	106.3	103.1	101.7	105.4	105.4	103.3
21	131.6		102.7	107.3	105.5	101.8	106	105.9	103.4
22	131.2	105	102.8	106.6	105.5	101.7	106.3	104.5	103.8
23	133	106.6	102.3	106.3	105.5	102	107.3	104.7	103.6
24	134.3	116.3	102	106.4	104.5	102.7	106.6	104.2	100.8
25	137.1	114.7	104.1	108.3	104	102.9	108.6	104.7	103.5
26	138.4	103.1	106.7	107.2	104.4	103.4	109.1	105.3	102.9
27	137.6	114.5	106.3	106.3	107.3	102.9	109.4	106.1	102
28	138.7	118.1	105.4	106.6	105.5	102.9	108.2	103.8	101.6
29	141.1	118.3	105	106.2	104.9	102.7	107.9	104.2	101.1
30	138.2	117.7	104.4	106.3	104	103.2	108.3	104.7	100
31	138.9	119.5	97.6	106	103.6	103	106.7	102.9	98
32	140.2	118.4	103.3	107.4	102.9	102.8	106.4	104.4	98.3
33	139.9	120.5	103.1	107	104.1	102.8	107	104.5	99.8
34	143.9	120	103.3	107.5	105.3	103	107.3	104.5	100.7
35	143.2	119.9	103.3	108.1	106.3	103.2	106.6	102.5	101.3
36	145	122.8	102.1	108.9	106.4	102.5	106.3	103.6	104.2
37	145	93.6	102.7	108.6	105.2	103	105.8	104.3	102.1
38	145.7	122.3	103.4	107.7	104.9	101.6	105.8	110.7	102.1
39	146.6	124.2	102.8	106.7	105.2	102.7	105.8	105.1	102.3
40	148.2	125.7	102.8	106	106.1	101.9	104.6	104.4	103.3

41	148.6	125.4	102.6	106.5	105.6	102.9	105.2	105.1	102.9
42	156	125.9	101.7	106.4	105.3	102.1	104.9	105.4	102.9
43	153.2	125.7	100.6	105	103.7	103.2	105.5	105.6	102.6
44	153.8	126.6	101.4	104.7	103.5	102.7	105.3	105.3	102.2
45	154.6	127	101.9	104.1	104.1	103.4	106	105.2	102.2
46		126.8	102	103.1	104.1	103.8	106	103.7	102.3
47	155	127.9	103.2	103.2	105.1	103.3	106.5	104.2	102.3
48	153.3	128.9	103.2	102.9	103.9	104.5	105.8	104.1	101.8
49	154.9	128.3	105.8	105	106.3	105.9	107.5	104.8	100.1
50	155.6	128.7	105.9	105.6	105.6	106.2	107.5	104.3	102.5
51	156.9	129.3	104.7	104.3	105.2	105.8	107.1	104.8	102.4
52	138	129.6	104.6	104.3	106	106.2	104	103.2	103
53	136.6	125.1	103.3	104	105.1	105.6	104.2	103.9	102.9
54	139.1	125.9	104.1	105.7	105.8	105.7	105.2	104	102.2
55	140	125.8	104.4	103.7	105.9	105.9	105.7	101.6	101.4
56	140.4	127.6	102.6	104.5	104.9	105.4	106.5	104.3	101.1
57	142.1	127.8	102.4	104.2	105.5	104.9	105.4	104	101.2
58	138.8	128.8	102.9	104.8	105.1	105.7	104.2	103.7	101.1
59	142.5	128.3	102.2	103.7	105.9	105.9	105.4	103.8	101.2
60	144.9	129.7	103.2	103.6	106.6	105	105.5	103	105.7
61	143.7	129.8	102.1	103.3	105.2	103.1	105.9	103.5	100.4
62	145.5	131.6	105.7	103.3	106.5	103.9	105.4	107.2	102.4
63	146.9	131.8	103.9	104.6	106.7	104.7	105.7	102.9	102.9
64	148.8	133.3	104.5	103.7	104.4	104	101.8	100.3	101.4
65	144.4	133.7	103.1	105	105.8	103.4	103.6	102.6	102.1
66	145	134.2	103.4		106.1	104.2	104.5	103	102.4
67	146	134.5	102.5		106.6	103.8	101.7	103	101.9
68	146.5	135.2	102.1		106.7	102.9	104.4	102.7	101.3
69	150.4	136.1	102.3	102.7	106.9	105.1	105.2	103.1	101.3
70	152.1	136.5	102.1	103.1	106.3	104.5	104.1	102.7	100.5
71	151.4	137.1	103.1	103.4	106.5	103	104.4	102.4	101.6
72	150.7	136.4	102.7	103.3	106.1	103.5	104.6	102.2	101
73	153.7	137.9	101.3	97.2	105.9	103.6	105.3	102.2	103.1
74	152.6	138.9	101.4	99.8	109	105.4	105.4	102.3	102.8
75	136.5	138.3	103.1	101.4	108.3	105	106.2	104.1	101.8
76	142.4	140	102	101.8	108.4	106.6	104.6	102.3	102
77	142.5	130.9	101.4	102.9	108.2	100.3	105.8	103	102.5
78	143.5	132	102.5	101.6	108.7	102	105.3	102.7	102.3
79	144	130.7	101.5	101.8	107.2	101.6	109.3	102.1	100.6
80	143.7	131.3	101	102.3	108.2	100.8	104	102.4	100.7
81	146.1	132	101.5	102.9	108.6	101.9	104.5	102.6	100.7
82	143.6	132.6	100.3	103.3	107.6	102.4	105.2	101.5	101.2
83	147	132	100.2	103.9		103.5	105.2	102	100.5
84	145.1	134	102.2	103.7	105.1	103.6	104.1	102.1	

85	146.4	134.3	103.3	103.5	105.8	103.4	103.1	100.2	
86	146.3	137.3	104.4	102.8	106.3	103.9	103.6		
87	142.8	135.3	105.7	103.3	106.6	103.8	104.4	102.1	
88	152.4	135.6		103.4	106.8	107.3	104.4	101.4	
89	152.1	134.7		104	106.7	105.6	103.8	102	
90	149.1	136		103.2	106.8	104.4	104.7	102.7	
91	149.5	138			106.4	104.2	103.1	102	
92	149.3	139			105.7	104.1	104	101.7	
93	149.9	139.3			105.7	105	105.4	101.7	
94	150	138.3			105.1	105.4	104.6	100.6	
95	149.8	141.4			105.8	104.1	104.1	101.3	104.6
96	150.6	139.5			104.8	104.2	104.5	101.5	104.1
97	144.5	140.9			106.1	105.2	105.2	102.3	100.8
98	147	140.5			106.3	107.1	105.4	102.7	104.5
99	149.8	141.1			105.9	106.1	105.3	102.2	104.6
100	152.4	140			105.7	106.8	104.3	101.8	103.9
101	131.5				105.4	106	104.6	102.2	102.8
102	139.1				104.8	105.2	105.1	103.1	103.2
103					104.5	105.8	105.6	101.6	102.1
104					104	104.8	105.2	102.3	101.9
105					103.7	103.4	104.6	102.8	102.6
106					103.6	103.1	103.7	102.8	102.5
107					104.1	102.7	104.1	101.5	102.5
108					103.7	102.4	103.8	102.6	
109					105.9	102.9	101.8	101.3	
110					104.5	101.5	103.9	101.2	
111					104.3	102.6	104.1		
112					104.1	105.2	103.8	101.1	
113					106.1	101.9	102.2	100.9	
114					104	101.8	103	101.4	
115					104.2	102.3	103	102	
116					104	102.4	105.3	101	
117					103.4	102.2	105.1	101.2	103
118					103.5	102.5	104.9	101.2	
119					104.2	102.1	105	102	104.4
120					103.6	102.7	104.9	101.9	104.5
121					103.8	102.2	106.3	99.7	98.7
122						104.2	106.5	101.7	103
123						102.7	106.3	102.2	105.4
124						103.9	104.9	101.5	105.3
125						103.7	104.8	101.7	103.7
126						104	105	102.3	102.7
127						104.1	104.5	102.1	99.5
128						103.6	105.8	101.5	101.6

129	103.3	105.7	103.2	103
130	105	100.6	99.5	103.4
131	104	105.3	102.9	103.3
132	103.5	105.2	103.4	102.7
133	103.2	104.9	102	102.2
134	102.6	104.1	106.5	103.1
135	103.1	104.6	102.6	103.3
136	103	103.8	103.4	103.3
137	102.6	104.1	102.4	103.2
138	102.5	104.6	103.4	103.7
139	102.4	106	101.8	103.4
140	101.8	104.3	102.9	103.2
141	102	103.2	103.3	102.8
142	101.6	101.1	103.8	103
143	101.6	102.8	103.9	98.9
144	101.6	104	104.5	98.3
145	101.9	106.6	105	100.6
146	102.1	105.8	105.2	102.3
147	101.5	107.2	104.5	102
148	102	106.5	104.3	101.4
149	102.8	104.9	102.7	102.6
150	102.8	105	102.8	103.2
151	102.5	107.1	99.3	102.4
152	102.2	103.9	102.9	103.4
153	102.3	104.9	102.6	104.8
154	102.1	102.5	101.4	104.1
155	102.4	103.4	102.5	103.9
156	103.2	103.8	103.1	103.3
157			103.1	102.6
158		104.1	103.1	102.9
159		104.1	103.9	102.7
160		104.1	102.1	102.1
161		103.3	102.8	103.7
162		103.6	103.3	101.8
163			102.3	102.7
164		103.7	102.8	102.6
165		104.1	103.6	102.8
166		103.5	104.3	104.3
167		103.4	103.6	103.6
168		104.1	105.1	101.8

Figure 5 Calcium (mmol/L)

Time (Hours)	A	B	C	D	1	2	3	4	5
0	0.406	0.955	0.835	0.778	0.698	0.661		0.137	0.817
1	0.449	0.632	0.425	0.315	0.724	0.647	0.572	0.662	0.433
2	0.387	0.754	0.48	0.357	0.945	0.731	0.746	0.883	0.59
3	0.547	0.889	1.516	0.654	1.176	0.728	0.946	0.928	0.758
4	0.847	1.014	1.157	0.86	1.224	0.907	1.262	1.166	0.864
5	1.202	1.041	1.173	1.042	1.332	0.885	1.324	1.153	0.883
6	1.292	1.057	1.233	1.148	1.414	0.858	1.344	1.205	0.941
7	1.277	1.037	1.242	1.243	1.462	0.832	1.356	1.277	0.999
8	1.254	1.036	1.239	1.28	1.477	0.801	1.361	1.351	0.976
9	1.146	1.042	1.24	1.302	1.384	0.916	1.344	1.386	1.007
10	1.211	0.997	1.202	1.334	1.434	1.061	1.372	1.449	1.004
11		1.078	1.206	1.34	1.416	1.161	1.373	1.405	0.95
12	1.214	0.994	1.151	1.346	1.297	1.23	1.327	1.443	0.988
13	1.173	1.037	1.081	1.355	1.393	1.295	1.312	1.382	0.937
14	1.145	0.195	1.19	1.382	1.406	1.327	1.308	1.354	0.968
15			1.281	1.345	1.358	1.35	1.315	1.352	0.909
16	1.099		1.326	1.336	1.21	1.381	1.32	1.342	0.895
17	1.093		1.346	1.357	1.268	1.364	1.346	1.331	0.89
18	1.094		1.358	1.323	1.27	1.379	1.328	1.304	0.877
19	1.03	0.628	1.39	1.32	1.322	1.412	1.321	1.313	0.861
20	1.025	1.178	1.4	1.303	1.35	1.394	1.294	1.315	0.946
21	0.999		1.405	1.294	1.171	1.384	1.279	1.312	0.876
22	0.963	1.008	1.378	1.298	1.234	1.388	1.285	1.333	0.863
23	0.956	1.569	1.392	1.321	1.264	1.384	1.263	1.334	0.876
24	0.952	0.471	1.297	1.326	1.329	1.352	1.309	1.386	0.799
25	0.906	0.806	1.063	1.215	1.163	1.363	1.157	1.235	0.636
26	0.905	0.579	0.924	1.293	1.18	1.244	1.197	1.282	0.539
27	1.003	1.17	1.02	1.358	0.983	1.233	1.222	1.274	0.573
28	1.032	0.624	1.11	1.365	1.081	1.269	1.245	1.293	0.554
29	0.98	0.533	1.164	1.361	1.124	1.318	1.282	1.287	0.559
30	0.81	0.668	1.225	1.353	1.182	1.35	1.291	1.298	0.564
31	0.814	0.599	1.235	1.357	1.106	1.368	1.271	1.344	0.525
32	0.855	0.674	1.297	1.317	1.181	1.381	1.298	1.379	0.528
33	0.854	0.717	1.326	1.328	1.19	1.388	1.315	1.391	0.569
34	0.859	0.754	1.331	1.328	1.229	1.397	1.3	1.379	0.757
35	0.839	0.748	1.343	1.358	1.214	1.452	1.317	1.383	0.8
36	0.831	0.755	1.351	1.278	1.225	1.438	1.286	1.367	0.816
37	0.82	0.654	1.354	1.29	1.229	1.438	1.32	1.374	1.023
38	0.83	0.801	1.347	1.353	1.262	1.46	1.309	1.375	1.004
39	0.834	0.738	1.367	1.345	1.299	1.415	1.339	1.363	1.032
40	0.809	0.649	1.392	1.305	1.312	1.416	1.38	1.357	1.044

41	0.831	0.759	1.401	1.292	1.335	1.379	1.398	1.329	1.004
42	0.809	0.725	1.451	1.358	1.353	1.401	1.341	1.335	1.016
43	0.822	0.703	1.53	1.374	1.344	1.389	1.335	1.338	1.014
44	0.804	0.679	1.548	1.39	1.407	1.393	1.317	1.327	1.01
45	0.822	0.663	1.391	1.44	1.395	1.361	1.318	1.331	1.048
46		0.651	1.377	1.465	1.401	1.347	1.285	1.344	1.075
47	1.09	0.66	1.349	1.445	1.376	1.364	1.326	1.368	1.076
48	1.145	0.624	1.328	1.415	1.405	1.303	1.339	1.371	1.115
49	1.244	0.637	1.215	1.275	1.261	1.279	1.17	1.241	0.937
50	1.222	0.858	1.303	1.3	1.298	1.17	1.207	1.271	0.868
51	1.204	0.625	1.345	1.303	1.345	1.177	1.264	1.317	0.976
52	0.789	0.589	1.358	1.28	1.327	1.243	1.347	1.383	1.074
53	0.895	0.558	1.396	1.244	1.326	1.265	1.355	1.391	1.073
54	0.987	0.541	1.398	1.225	1.304	1.279	1.359	1.348	1.049
55	0.954	0.496	1.398	1.296	1.314	1.281	1.324	1.333	1.059
56	0.928	0.478	1.356	1.348	1.33	1.283	1.343	1.34	1.109
57	0.913	0.472	1.35	1.403	1.328	1.266	1.307	1.363	1.122
58	0.841	0.464	1.358	1.41	1.346	1.309	1.325	1.371	1.148
59	0.864	0.457	1.395	1.426	1.325	1.314	1.325	1.403	1.198
60	0.847	0.436	1.332	1.435	1.304	1.336	1.334	1.401	1.196
61	0.837	0.457	1.305	1.441	1.32	1.413	1.329	1.395	1.194
62	0.802	0.411	0.956	1.442	1.321	1.413	1.343	1.398	1.223
63	0.78	0.439	1.079	1.44	1.312	1.385	1.318	1.397	1.232
64	0.892	0.424	1.15	1.458	1.313	1.395	1.344	1.394	1.232
65	0.885	0.416	1.199	1.435	1.358	1.392	1.388	1.392	1.238
66	0.885	0.433	1.237	1.456	1.326	1.394	1.376	1.367	1.259
67	0.88	0.416	1.254	1.448	1.306	1.449	1.348	1.393	1.274
68	0.853	0.404	1.264	1.434	1.308	1.445	1.353	1.402	1.243
69	0.877	0.396	1.289	1.369	1.327	1.108	1.348	1.4	1.301
70	0.882	0.39	1.281	1.353	1.342	1.199	1.326	1.407	1.265
71	0.952	0.406	1.259	1.352	1.346	1.281	1.333	1.422	1.226
72	0.958	0.383	1.255	1.294	1.35	1.338	1.296	1.386	1.245
73	1.004	0.396	1.21	1.193	1.26	1.397	1.192	1.261	1.096
74	0.941	0.386	1.16	1.237	1.235	1.132	1.218	1.289	1.04
75	0.696	0.379	1.124	1.264	1.25	1.141	1.235	1.253	1.097
76	0.878	0.371	1.192	1.281	1.254	1.156	1.284	1.285	1.155
77	0.88	0.31	1.174	1.282	1.287	1.197	1.276	1.318	1.163
78	0.861	0.327	1.186	1.348	1.251	1.267	1.354	1.365	1.197
79	0.856	0.35	1.216	1.343	1.217	1.355	1.357	1.368	1.211
80	0.838	0.349	1.23	1.328	1.198	1.347	1.398	1.422	1.281
81	0.808	0.354	1.266	1.326	1.215	1.377	1.382	1.41	1.308
82	0.785	0.349	1.294	1.312	1.297	1.358	1.368	1.414	1.294
83	0.823	0.35	1.33	1.296		1.346	1.384	1.394	1.301
84	0.822	0.325	1.303	1.307	1.316	1.35	1.401	1.406	

85	0.858	0.35	1.205	1.284	1.341	1.338	1.374	1.425	
86	0.828	0.316	1.139	1.277	1.338	1.389	1.364	1.415	
87	0.796	0.348	1.058	1.283	1.328	1.38	1.383	1.424	
88	0.789	0.36		1.288	1.337	1.037	1.37	1.432	
89	0.794	0.339		1.301	1.333	1.143	1.395	1.417	
90	0.813	0.349		1.262	1.323	1.228	1.351	1.41	
91	1.021	0.32			1.352	1.276	1.422	1.429	
92	1.017	0.331			1.369	1.3	1.36	1.423	
93	1.043	0.317			1.374	1.297	1.346	1.436	
94	1.052	0.318			1.408	1.341	1.348	1.445	
95	1.155	0.312			1.319	1.327	1.396	1.457	1.289
96	1.233	0.318			1.368	1.404	1.392	1.448	1.337
97	1.415	0.321			1.303	1.311	1.299	1.291	1.162
98	1.214	0.328			1.336	1.165	1.292	1.3	1.103
99	1.446	0.314			1.332	1.202	1.369	1.292	1.062
100	1.478	0.324			1.376	1.18	1.383	1.265	1.043
101	1.092				1.362	1.284	1.406	1.269	1.118
102	1.086				1.371	1.371	1.375	1.249	1.166
103					1.394	1.352	1.371	1.252	1.189
104					1.421	1.371	1.374	1.31	1.17
105					1.438	1.427	1.365	1.324	1.258
106					1.45	1.417	1.398	1.344	1.341
107					1.461	1.424	1.417	1.363	1.352
108					1.442	1.435	1.43	1.369	
109					1.412	1.434	1.442	1.384	
110					1.409	1.42	1.445	1.42	
111					1.422	1.396	1.429	1.434	
112					1.419	1.401	1.384	1.408	
113					1.382	1.423	1.375	1.412	
114					1.386	1.422	1.386	1.413	
115					1.371	1.438	1.36	1.42	
116					1.395	1.442	1.302	1.425	
117					1.377	1.419	1.354	1.505	1.33
118					1.372	1.439	1.369	1.436	1.316
119					1.341	1.47	1.389	1.25	1.347
120					1.338	1.49	1.395	1.361	1.366
121					1.305	1.476	1.263	1.372	1.161
122						1.3445	1.273	1.372	1.089
123						1.364	1.274	1.367	1.132
124						1.346	1.335	1.363	1.245
125						1.333	1.39	1.377	1.255
126						1.315	1.411	1.383	1.246
127						1.311	1.353	1.41	1.226
128						1.277	1.365	1.371	1.256

129	1.267	1.394	1.37	1.274
130	1.262	1.421	1.377	1.308
131	1.25	1.414	1.422	1.325
132	1.241	1.398	1.409	1.357
133	1.227	1.408	1.371	1.317
134	1.222	1.433	1.403	1.314
135	1.292	1.404	1.409	1.355
136	1.334	1.407	1.412	1.412
137	1.371	1.394	1.413	1.432
138	1.391	1.389	1.359	1.413
139	1.408	1.367	1.39	1.398
140	1.441	1.397	1.408	1.436
141	1.433	1.438	1.382	1.441
142	1.443	1.3	1.376	1.351
143	1.451	1.317	1.28	
144	1.455	1.267	1.255	1.301
145	1.412	1.61	1.193	1.23
146	1.418	1.189	1.295	1.196
147	1.468	1.158	1.281	1.149
148	1.421	1.281	1.25	1.105
149	1.364	1.329	1.302	1.04
150	1.371	1.355	1.334	1.1
151	1.388	1.371	1.391	1.154
152	1.4	1.392	1.404	1.223
153	1.415	1.39	1.444	1.255
154	1.422	1.419	1.373	1.302
155	1.382	1.423	1.404	1.331
156	1.511	1.43	1.403	1.398
157		1.452	1.38	1.392
158		1.461	1.403	1.429
159		1.441	1.379	1.449
160		1.418	1.405	1.477
161		1.384	1.377	1.483
162		1.387	1.34	1.496
163		1.401	1.343	1.469
164		1.391	1.268	1.502
165		1.416	1.245	1.487
166		1.419	1.232	1.357
167		1.427	1.2	1.325
168		1.437	1.222	1.33

Figure 6 **Bile pH**

Time (Hours)	1	2	3	4	5
6	7.809		7.499	7.666	6.962
9				7.713	
12				7.801	
15			7.862	7.778	
16					7.663
18	7.875		7.722	7.757	7.932
21			7.779	7.789	7.761
24	8	7.503	7.788	7.88	7.688
27	8		7.66	7.843	
30	7.92		7.772	7.867	7.639
33			7.969	7.696	
36	8	7.577	7.88	7.76	7.568
39			7.822		7.647
42	8	7.548	7.903	7.841	7.485
45			7.819	7.777	7.418
48	8	7.566	7.912	7.675	7.613
51			8	7.756	
54	7.605		7.949	7.795	7.788
57			8	7.598	
59	7.649				
60		7.753		7.624	7.502
63	7.782		8	7.692	7.917
66		7.553	7.792	7.708	7.45
69			8	7.663	7.526
72	7.841	7.827	7.665	7.58	7.719
75			7.766	7.687	
78	7.927		7.644	7.663	7.61
81			7.748	7.658	
84	7.901	7.686	7.819	7.663	7.769
87			7.691	7.583	7.527
90		7.505	7.628	7.673	7.809
93		7.666	7.58	7.703	7.679
96	7.906	7.85	7.699	7.637	7.634
99			7.865	7.633	
102	7.655		7.835	7.671	7.579
105			7.711		
106				7.591	
108		7.61	7.825	7.684	7.696
111				7.635	7.631
112			7.716		
114	7.666	7.544	7.764	7.661	7.798

115				7.633	
117			7.743		7.516
120	7.591	7.972	7.795	7.694	7.631
123		7.654	7.892	7.742	
126		7.653	7.898	7.649	7.713
129		7.793	7.921	7.683	
132		7.308	8	7.693	7.701
135			7.94	7.616	7.955
138		7.42	8	7.708	7.579
141			8	7.693	
142					
144			8	7.634	7.54
146			7.84		
147			8	7.737	
150			8	7.714	7.722
153			7.99	7.736	
154					
156			8	7.75	7.706
159			8	7.619	7.696
162			8	7.733	7.924
165			7.976	7.677	7.766
168			8	7.672	7.774

Figure 6 Glucose (mmol/L)

Time (Hours)	Liver 1	Liver 2	Liver 3	Liver 4	Liver 5
6	5.57		40	20.14	40
9				3.57	
12				1.08	
15			1.9	1.13	
16					29.84
18	1.08		1.95	0.93	5.98
21			1.58	0.79	
24	0.66	1.34	1.55	0.82	2.33
27	0.68		1.47	0.87	
30	1.85		1.1	0.68	4.99
33			1.14	0.67	
36	1.22	2.35	0.66	0	3.81
39			0.56		5.18
42	0.71	1	0.56	0	0.95
45			1.02	0.51	1.06
48	0.67	0.94	0.62	0	0.62
51			0.55	0	
54	0		0.51	0	0.63
57			0.51	0	
59	0.68				
60		0.52		0	0.53
63	0.59		0.54	0	0.53
66		1.27	0.51	0	0.53
69			0.51	0	0.53
72	0.6	0.84	1.18	0	0.53
75			3.99	0	
78	0.7		2.34	0	0.56
81			1.39	0	
84	0.72	1.94	1.37	0	0.52
87			2.93	0	0.53
90		1.18	2.63	0	0.53
93		0.68	2.3	0	0.53
96	0.87	0.79	2.52	0	0.52
99			2.73	0	
102	0.86		1.53	0	0.55
105			2.18		
106				0	
108		0.65	1.74	0	0.53
111				0	0.67
112			1.65		
114	2.01	0	1.69	0	0.52

115				0	
117			1.36		0.52
120	1.29	0.53	1.08	0	0.78
123			1.12	0.94	
126			0.81	0.64	2.06
129			0.58	0.65	
132		0.51	0.53	0.58	0.52
135			0.52	0	0.53
138		0.76		0.58	0.52
141			0.58	0.55	0.95
142		0.51			
144		4.85	0.56	0.5	
146			0.58		
147			0.72	0.86	
150				0	0.6
153			0.62	0.57	
154		3.5			
156			0.62	0	0.53
159			0.61	0	0.53
162			0.54	0	0.53
165			0.54	0	0.53
168			0.54	0.51	0.91

Fgure 6 Bile Delta Glucose (mmol/L)

Time	Liver 1	Liver 2	Liver 3	Liver 4	Liver 5
6	1.28		-26.03	-2	0
24	3.15	4.32	5.32	6.72	3.87
30	24.54		5.41	7.89	0.38
36	5.84	4.21	6.41	6.95	0.1
42	4.83	4.33	6.6	7.77	4.14
48	5.18	3.93	8.41	7.49	10.72
54	6.38		4.41	6.06	3.96
60		5.6		7.21	2.64
66		7.5	6.64	7.46	6.03
72	4.64	8.99	6.42	3.59	3.87
78	5.8		4.11	7.05	2.45
84	5.12	4.36	3.31	6.28	4.98
90		3.48	4.29	6.48	4.66
96	3.14	4.64	4.75	6.07	4.56
102	3.1		5.09	4.73	13.32
108		5.73	4.7	6.42	6.98
114	4.03	4.33	5.36	6.44	2.97
120	5.01	5.11	5.94	37.06	4.93
126			5.3	17.49	17.34
132		1.11	7.39	5.33	5.16
138		4.69		7.74	8.76
144			10.09	6.06	
150				7.12	1.68
156			5.05	6.7	5.85
162			6.25	8.14	4.14
168			6.93	12.54	5.01

Figure 6 **Cumulative Bile Production (ml)**

Time (Hours)	Liver 1	Liver 2	Liver 3	Liver 4	Liver 5
0	0	0	0	0	0
6	25		52	20	7
9			117	55	
12	85		177	95	
16					28
17				150	
18	152		222		38
21				197	58
22			282		
24	197	65	302	221	73
26		130			
27			337	266	
29		190			
30	237		382	291	93
32		250			
33				336	
35		315			
36	270	347		376	115
38		397			
39			437		132
41				436	
42	316	432	482		
45		502	507	481	143
48	375	562	532	526	147
51			542	569	
52		622			
54	445		592	613	157
55		682			
57				657	
59	505				
60		737	647	702	169
63	560				179
64		797		762	
66		837	702		193
67	617				
68				812	
69					204
72	675	907	745	867	215
75			785	892	
76		967			
78	745		828	933	233

80		1027			
81			888	979	
84	810	1047		1019	249
86			953		
87				1074	263
88	870				
89		1107			
90		1127	1008		274
92	930				
93			1048	1088	285
95		1197			
96	977	1207	1078	1126	298
99			1118	1161	
100	1029	1267			
102	1039		1172	1201	328
104		1327			
105				1249	
107			1237		
108	1099	1347		1293	352
110		1407			
111					366
112		1457	1272	1353	
114	1149	1477	1297		377
115				1398	
116		1542			
117			1337	1418	388
119		1607			
120	1189		1372	1455	404
123		1667	1592	1477	
126		1727	1645	1504	444
129		1787		1532	
131			1700		
132		1842		1568	
135		1862			455
136		1882			
138		1932	1750	1618	462
141			1760	1662	
142					510
143		1992			
144		2062	1774	1692	523
144.5		2122			
145		2182			
146		2242	1786		
147			1791	1732	

148	2302			
149	2367			
150		1818	1771	543
151	2432			
152	2472			
153			1812	
154	2537			583
156			1841	611
158		1873		
159			1891	658
162		1915		685
165		1940	1946	707
168		1970	1996	723
171		1995		
174		2019		
182		2039		

Table 2 **ALT (U/L)**

Time (Hours)	A	B	C	D	1	2	3	4	5
0	0	0	0	0	0	0	0	0	0
1	545	6727	1402	1546	2447	1726	1570	915	11577
2	790	7137	1712	1699	2504	1925	1757	1092	11217
3	960	7123	1784	1868	2532	2118	1899	1176	11279
4	1055	7010	1877	1876	2511	2200	1873	1212	11406
5	1070	7397	1793	1936	2453	2317	1811	1221	11288
6	1090	7594	1828	1758	2399	2418	1799	1163	11064
8	1055								
9	1205	8417	2063						
11					2295				
12	1655	8463	2294	2130	2270	1846	2315	1117	10084
18	3235	6672	2516	2448	2185	2233	2609	1326	10642
21					1757				
23						3317			
24	4140	9009	2556	3005	1790	3356	3177	1534	10018
26					1806				
27					1431				
28		9671							
29		5063							
30	3280	4857	2470	3114	1466	3731	3508	1487	9385
31					1349				
36	4395	5075	2621	2962	1598	3496	3286	1351	9034
40				2978					
42	4885	4823	3982		1747	3267	3519	1500	8417
46									
47						2900			
48	4965	4821	2792	2739		2860	3071	1644	6647
51					1690				
52					1626				
53		2763							
54	4415	2783	3290	2691	1617	2619	2713	1572	7776
58					1578				
60	6370	2723	3368	2853	1509	2492	2511	1241	7271
62			2422						
66	7915	2779	2570	3149	1403	2528	2396	1425	6954
72	5395	2853	3460	2867	1343	1454	1953	1512	6587
74					1227				
75		2906							
76	2540				1166				
77		1726							
78	1800	1611	5564	4518	1152	1375	1814	1314	5732

83					1078				
84	6060	1629	8572	4616	1030	1190	1621	1194	5038
87			9014						
89				4668					
90	5420	1719		5335		705	1480	1204	4791
96	4800	1786			893	751	1231	1262	4010
100	6045	1819							
102	3080				836	740	934	1168	3211
108					783	737	848	1096	3148
114					615	697	844	1048	2858
120					223	746	672	967	2597
126						722	629	892	2281
132						765	693	798	2192
138						810	797	737	1999
144						870	663	617	1745
150						800	640	553	1678
152						798			
154						850			
156						862	623	520	1532
162							598	489	1523
168							575	429	1388
174							582		
180							646		
184							678		

Table 2 **AST (U/L)**

Time	A	B	C	D	1	2	3	4	5
0	0	0	0	7	0	0	0	0	0
1	720	15632	1422	1682	3181	1818	1854	1272	21928
2	1000	15689	1727	2039	3348	1956	1979	1604	19846
3	1185	16054	1828	2270	3264	2276	2103	1722	18961
4	1375	16334	1893	2459	3375	2295	2180	1786	17529
5	1500	16645	1931	2603	3474	2448	2134	1840	15922
6	1710	17462	2100	2734	3468	2748	2247	1900	14423
8	1785								
9	2140	24601	2702						
11					4154				
12	3030	28632	3185	4623	3968	3157	3599	2509	20800
18	5995	28754	4064	5601	4402	3673	4927	3641	29223
21					3470				
23						4716			
24	6505	50495	4536	6623	3713	4588	7246	4612	22821
26					3801				
27					2953				
28		53418							
29		26991							
30	6195	26518	4285	7455	2969	5464	8235	4526	6276
31					2788				
36	7120	29366	4625	7096	2956	4903	7326	4160	5781
38									
40				6557					
42	7850	31419	2286		3187	4487	7487	3787	4590
46									
47						3994			
48	8470	34699	4920	5402		3920	6452	3249	8189
51					3363				
52					3060				
53		19081							
54	8040	19850	5724	5445	3237	3617	5423	2708	7884
58					3289				
60	12250	20696	6234	5616	3475	3171	3965	2389	14904
62			4576						
66	15365	24385	2973	6777	3368	2740	2698	2008	16976
72	10490	27208	10860	6039	3197	1543	1955	1634	28573
74					3061				
75		28834							
76	6040				2824				
77		15631							

78	4550	14774	18338	2749	1321	1537	1189	27646
83				2600				
84	12865	15613		2455	1127	971	1088	24692
87								
90	12075	17159			597	631	885	22166
96	9305	18249		2030	517	499	740	17820
100	12400	18309						
102	7780			1958	457	324	607	14929
108				1934	420	261	515	13718
114				1999	428	216	435	12911
120				2560	456	252	326	11576
126					548	239	251	10362
132					624	235	213	9873
138					829	237	176	8990
144					1073	294	141	7646
150					1276	289	125	7302
152					1385			
154					1517			
156					1890	269	113	6635
162						232	96	6491
168						357	79	6113
174						422		
180						531		
184						671		

Table 2 **ALP (U/L)**

Time (Hours)	C	D	1	2	3	4	5
0	0	0	0	0	0	0	0
1	15	20	86	15	21	42	40
2	20	22	73	12	11	30	90
3	35	24	60	14	8	34	109
4	58	23	48	14	7	42	123
5	86	28	37	16	8	52	130
6	108	30	33	19	11	59	135
9	153						
11			28				
12	187	47	27	27	23	90	77
18	201	64	43	49	43	115	56
21			38				
23				128			
24	200	87	43	110	86	140	70
26			46				
27			35				
30	186	102	36	167	145	143	94
31			38				
36	196	100	41	167	137	143	121
40		108					
42	174		59	162	161	141	169
47				154			
48	212	101		145	152	152	197
51			41				
52			37				
54	262	108	39	142	251	143	248
58			41				
60	320	117	70	138	218	140	324
62	228						
66	484	134	43	134	234	139	432
72	972	105	40	93	632	149	528
74			46				
76			45				
78	1940	230	51	85	507	131	596
83			46				
84	4196	291	61	90	483	131	684
87	4658						
89		363					
90		392		57	406	123	803
96			91	63	341	135	808
102			144	67	247	128	852

108	176	70	252	125	967
114	136	75	232	120	1069
120	211	83	214	116	1111
126		106	209	108	1044
132		126	234	103	991
138		118	289	103	918
144		119	340	94	816
150		140	358	83	829
152		210			
154		280			
156		300	331	79	820
162			292	80	847
168			266	78	823
174			254		
180			344		
184			437		

Table 2 **GGT (U/L)**

Time (Hours)	B	C	D	1	2	3	4	5
0	0	0	0	0	0	0	0	0
1	47	50	0	31	16	7	15	26
2	50	81	17	34	20	5	19	36
3	55	132	27	31	32	5	34	39
4	70	201	32	30	36	6	54	43
5	91	253	37	31	43	5	70	45
6	117	310	40	36	55	8	82	49
9	220	440						
11				50				
12	300	501	65	50	110	10	114	86
18		539	99	70	137	18	118	158
21				65				
23					188			
24	630	545	141	76	175	26	111	224
26				78				
27				61				
28	714							
29	360							
30	356	500	178	65	183	56	100	294
31				62				
36	417	568	177	70	169	57	97	363
40			198					
42	443	434		96	165	74	95	460
47					156			
48	474	422	207		147	72	100	499
51				72				
52				65				
53	293							
54	314	480	255	67	144	149	96	547
58				69				
60	359	442	288	95	129	145	95	604
62		212						
66	434	220	317	66	126	149	96	649
72	491	246	195	68	95	479	101	669
74				67				
75	537							
76				66				
77	324							
78	316	278	378	71	89	390	90	656
83				73				
84	370	314	410	69	90	351	92	670

87		334						
89			407					
90	521		430		57	294	89	704
96	572			77	76	241	94	677
100	602							
102				111	80	178	90	659
108				135	83	180	89	690
114				164	92	168	87	724
120				196	95	155	85	737
126					100	149	80	673
132					104	170	82	635
138					105	219	90	569
144					109	244	82	503
150					113	268	73	525
152					128			
154					134			
156					129	255	68	537
162						239	69	569
168						202	75	559
174						190		
180						245		
184						292		

Table 2 **CRP (mg/L)**

Time	A	B	C	D	1	2	3	4	5
0	6.5	0	0	0	0	0	0	0	0
1	19.5	15.8	2.6	5.9	0	0	3.6	2.6	4.3
2	36	28.2	8.4	19.4	2.8	1.4	9.4	6.4	4.8
3	49	41.5	14.2	35.8	4.7	2.8	14.7	9.4	5.8
4	62	53.5	19.6	44.5	6.7	3.5	21.2	12.9	7.2
5	68	64.5	25.3	54.7	8.7	4.6	25.6	15.9	8.8
6	79	76.1	30.9	65.4	10.4	6.3	30.9	18.9	10.8
8	87								
9	102.5	112	57.5						
11					27.1				
12	128	137	85.7	140	29.7	19.2	64.4	41.5	27.7
18	160.5	143	129	165	47.3	43.3	71.1	55.9	47
21					41.6				
23						64.9			
24	188.5	200	160	185	47.1	61.8	69.3	58.6	59.4
26					51.5				
27					40.2				
28		213							
29		116							
30	184	113	178	167	42.4	80.6	68.7	53.6	70.5
31					39.9				
36	211.5	126	214	148	43	74.6	56	46.7	60.8
38									
40				143					
42	239.5	126	180.4		46.3	71.3	51.6	42.5	48.3
46									
47						67.1			
48	206	133	202	147		61.1	42.7	39.3	46.4
51					48.3				
52					45.8				
53		76.9							
54	119.5	81.4	208	170	47.5	56.9	39.2	34.9	41.1
58					51.9				
60	125	89.3	236	180	53.3	45.8	32.8	30.2	37.4
62			129						
66	130.5	99.8	115	198	57.3	37.3	31.8	27.9	34.3
72	91.5	107	125.4	139	62.6	21.8	33.4	26.7	32.8
74					58.8				
75		112							
76	51.5				57				
77		65.2							

78	40	61.3	113.8	231	62.8	24.2	36.2	22.4	31.9
83					66.6				
84	87.5	66.1	98	247	66	63.4	41	20	27.9
87			102						
89				237					
90	78	75		276		69.4	40.4	18.1	26.8
96	85.5	79.7			111	101	38.4	17.3	23.5
100	91.5	82.1							
102	55.5				128	143	30.6	16.3	23.2
108					120	172	28.8	14.3	20.5
114					142	233	28	12.9	20.9
120					155	270	24.4	11.4	18.8
126						313	21.6	10.4	19.7
132						348	24.2	9	20.5
138						340	28.1	8.4	33.1
144						257	31.4	8.2	42.1
150						278	33.6	8.8	67.9
152						236			
154						253			
156						275	48.7	10.6	109
162							81.1	12.3	147
168							124	15.1	198
174							176		
180							228		

Table 2 **Direct Bilurubin (mmol/L)**

Time	A	C	D	1	2	3	4	5
0	0	0	0	0	0	0	0	0
1	0	0	0	0	0	4	4	0
2	0	0	4	0	0	6	6	3
3	0	6	5	0	4	9	7	0
4	0	9	5	4	5	10	8	4
5	0	12	5	5	5	10	9	4
6	0	10	5	5	8	11	10	4
8	0							
9	0	16						
11				8				
12	20	27	0	8	19	7	7	7
18	30	42	0	11	26	4	0	13
21				13				
23					32			
24	40	63	0	12	19	5	0	18
26				11				
27				15				
30	75	59	0	19	8	0	0	32
31				16				
36	105	59	0	12	6	0	0	39
40			6					
42	165	60		28	8	3	0	54
47					8			
48	100	86	21		7	6	0	58
51				9				
52				15				
54	65	130	59	15	9	9	0	75
58				15				
60	95	84	103	17	13	0	0	90
62		76						
66	105	32	157	13	11	8	0	106
72	0	0	8	16	58	16	0	118
74				18				
76	0			17				
78	0	26	170	18	36	7	0	129
83				13				
84	40	20	205	15	36	8	0	133
87		0						
89			201					
90	0		217		58	7	0	169
96	0			21	94	8	0	172

100	0							
102	30			33	96	8	0	173
108				54	101	8	5	216
114				80	101	9	4	223
120				95	125	8	11	234
126					161	8	6	233
132					204	10	12	239
138					250	20	12	217
144					314	39	13	191
150					396	41	13	219
152					418			
154					429			
156					428	43	12	218
162						45	10	228
168						32	9	231
174						47		
180						85		
184						120		

Table 2 Total Bilirubin (mmol/L)

Time (Hours)	A	C	D	1	2	3	4	5
0	0	0	0	0	0	0	0	0
1	0	0	5	0	4	7	6	4
2	0	4	5	3	4	10	7	4
3	0	8	6	4	6	13	9	4
4	0	14	8	6	6	14	10	5
5	0	19	6	7	8	15	11	6
6	0	27	5	8	11	16	13	6
8	0							
9	20	47						
11				11				
12	55	65	0	9	23	10	9	10
18	80	108	0	16	31	6	0	18
21				18				
23					37			
24	100	156	0	29	25	9	0	24
26				32				
27				26				
30	140	201	0	30	12	5	0	44
31				26				
36	185	239	0	32	9	0	4	56
40			6					
42	270	194		71	14	5	0	75
47					13			
48	260	204	18		13	10	0	81
51				33				
52				24				
54	130	234	59	25	15	17	0	104
58				25				
60	155	178	109	43	20	3	0	128
62		106						
66	200	76	172	34	17	12	0	141
72	135	60	6	32	73	28	0	157
74				31				
76	70			26				
78	55	98	183	26	47	9	0	177
83				20				
84	165	76	234	17	58	11	0	191
87		72						
89			232					
90	145		266		102	10	0	229
96	155			24	146	13	0	234

100	160							
102	90			40	135	10	0	269
108				68	115	10	7	304
114				104	136	10	5	320
120				127	168	11	15	338
126					214	11	8	338
132					265	12	17	341
138					342	23	17	305
144					462	64	20	262
150					523	56	21	300
152					535			
154					587			
156					591	57	21	295
162						51	13	309
168						46	15	315
174						61		
180						111		
184						148		
